# Off‐Set Interactions of Ruthenium–bda Type Catalysts for Promoting Water‐Splitting Performance

**DOI:** 10.1002/anie.202101931

**Published:** 2021-05-19

**Authors:** Brian J. J. Timmer, Oleksandr Kravchenko, Tianqi Liu, Biaobiao Zhang, Licheng Sun

**Affiliations:** ^1^ Department of Chemistry School of Engineering Sciences in Chemistry, Biotechnology and Health KTH Royal Institute of Technology 10044 Stockholm Sweden; ^2^ State Key Laboratory of Fine Chemicals Institute of Artificial Photosynthesis DUT-KTH Joint Education and Research Centre on Molecular Devices Dalian University of Technology 116024 Dalian China; ^3^ Center of Artificial Photosynthesis for Solar Fuels School of Science Westlake University 310024 Hangzhou China

**Keywords:** homogeneous catalysis, kinetics, noncovalent interactions, ruthenium, water splitting

## Abstract

O−O bond formation with Ru(bda)L_2_‐type catalysts is well‐known to proceed through a bimolecular reaction pathway, limiting the potential application of these catalysts at low concentrations. Herein, we achieved high efficiencies with mononuclear catalysts, with TOFs of 460±32 s^−1^ at high catalyst loading and 31±3 s^−1^ at only 1 μM catalyst concentration, by simple structural considerations on the axial ligands. Kinetic and DFT studies show that introduction of an off‐set in the interaction between the two catalytic units reduces the kinetic barrier of the second‐order O−O bond formation, maintaining high catalytic activity even at low catalyst concentrations. The results herein furthermore suggest that π–π interactions may only play a minor role in the observed catalytic activity, and that asymmetry can also rationalize high activity observed for Ru(bda)(isoq)_2_ type catalysts and offer inspiration to overcome the limitations of 2nd order catalysis.

## Graphical abstract

Tuning the position of substituents on the axial ligand of the popular Ru(bda)L_2_ water‐splitting catalyst can enable catalysis at low concentrations with high turnover frequencies. Kinetic results indicate superiority of asymmetric catalysts and DFT studies show that this is through positioning of two catalytic units in parallel planes minimizing the X–π distance and optimizing stacking.

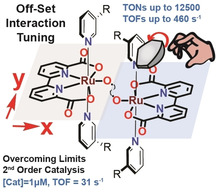

## Introduction

The global energy consumption cannot be sustained following our current trajectory. Increased accessibility to modern technology combined with the growing world population projected stringent limitations on our fossil fuel reserves. At the current pace, natural gas and oil reserves are predicted to last less than a single generation. The finite fossil fuels, together with the concurrent environmental problems associated with their consumption, place a great demand on the development of renewable carbon‐neutral or carbon‐free fuels.

Water, covering over 70 % of the Earth's surface, is a promising potential energy carrier for sustainable energy sources. Through water splitting this abundant resource can be converted into the high energy‐density fuel—hydrogen gas. This water splitting process proceeds through two half‐reactions, i.e., water oxidation (2 H_2_O→O_2_+4 H^+^+4 e^−^, *E* ^0^=1.23 V at pH 0) and subsequent proton reduction (2 H^+^+2 e^−^→H_2_, *E* ^0^=0.00 V at pH 0). Out of these two half‐reactions, water oxidation has been considered the most demanding as it includes a kinetically complex oxygen‐oxygen bond formation under the loss of four protons and four electrons.^[1]^

To facilitate and better understand the crucial oxygen‐oxygen bond formation, a wide variety of molecular catalysts has been developed over the last three decades.[^1b^, ^2^] For the ruthenium‐based catalysts, initial studies based on the design of the catalyst having two metallic centers in proximity of each other.^[3]^ Development progressed by the observation that water oxidation catalysis can also be achieved with mononuclear catalysts.[^3c^, ^4^] Finally, highly coordinated complexes bearing anionic ligands were developed to stabilize the high‐valent states of ruthenium.^[5]^ Currently, improvements on the catalytic activity are still being achieved by fine‐tuning of the direct coordination environment around the ruthenium center.^[6]^ To date the Ru(bda)L_2_ type catalysts (bda=2,2′‐bipyridine‐6,6′‐dicarboxylate) still remain amongst the most efficient molecular catalysts for oxygen‐oxygen bond formation.^[7]^ In addition, Nishibayashi, Sakata and co‐workers recently showed that Ru(bda)L_2_ complexes are also active in N−N bond formation with their work on oxidative conversion of ammonia into dinitrogen.^[8]^

**Figure 1:**
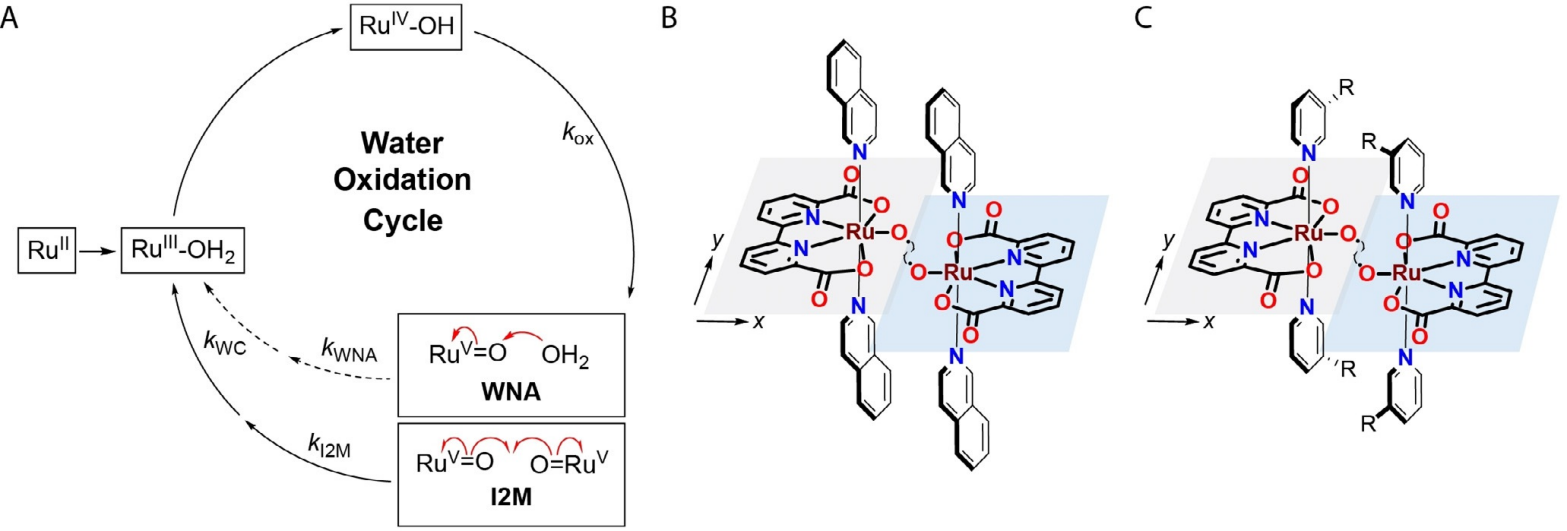
A) Water nucleophilic attack (WNA) vs. bimolecular radical–radical coupling (I2M) mechanism on Ru^II^‐catalysts. B) Graphical representation of the encounter complex of Ru(bda)(isoq)_2_ (isoq=isoquinoline) as calculated by Sun and co‐workers^[13]^ and C) hypothesized similar de‐symmetrization of the interaction by introduction of *meta*‐substitution pattern on the axial ligands.

The efficiency of the Ru(bda)L_2_ family of catalysts is dependent on the ability of two metal‐oxo (M=O) units to form the oxygen‐oxygen bond (I2M pathway, Figure 1 A), as compared to the water nucleophilic attack to the high valent M=O species (WNA pathway, Figure 1 A).^[9]^ High efficiency of the catalysts operating via the I2M pathway can be attributed to a second‐order dependence of reaction rate on the catalyst concentration. On one hand, this allows to reach high turnover frequencies at high catalyst concentrations. On the other hand, this limits the practical applications of the catalysts where at lower concentrations the monomolecular mechanism becomes dominant. This is in sharp contrast with, for example, iridium catalysts following the WNA pathway, where catalysis has been performed at concentrations as low as 0.5 μM.^[10]^

Understanding how to maintain rapid catalysis through the I2M pathway without requiring high catalyst concentrations has been of high interest. Many studies have been dedicated to investigating the electronic and secondary influence of the axial ligands on catalyst stability and reaction kinetics. The electronic effects of the axial ligands on oxygen evolution have initially been systematically investigated by the Sun group. From their studies it is apparent that introduction of electron withdrawing groups on the axial ligands improves the catalyst activity towards water oxidation, whereas the introduction of electron‐donating functionalities deterred catalysis.^[11]^ However, the presence or absence of electronic effects yet remains unclear as Murata and co‐workers clearly show that electron donating groups can present an advantage to catalysis as well.^[12]^ One aspect that arises from most of the ligand studies is that secondary effects facilitate the I2M pathway by minimizing the energy required to bring two catalytic units together, e*.g*., hydrophobic, π‐π‐stacking, dispersive and electrostatic interactions.^[14]^

Minimizing the energy required for the bimolecular O−O bond formation step is essential for achieving catalysis with Ru(bda)L_2_ at low concentrations. Multimeric catalysts have been developed as a strategy to overcome the limitation of the 2^nd^ order behavior, however, the often required complicated synthesis of these complexes is not ideal.^[15]^ Supramolecular approaches to achieve cooperative activation has proven to be a feasible and effective strategy.^[16]^ In supramolecular ruthenium complexes, H‐bonding networks are considered to play a large role in achieving more efficient catalysis.^[17]^ Whereas, confining the catalyst in cages^[18]^ and self‐assembled structures^[19]^ achieves higher local concentrations. For mono‐nuclear catalysts under homogeneous conditions, Concepcion and co‐workers elegantly show that the rate‐determining step can also be manipulated through introduction of ‐CF_3_ groups on the bda backbone. Through this modification the O‐O radical coupling is less determinant for the overall kinetics of the reaction and first order oxidation dependent behavior is extended towards the lower catalyst concentration regime.^[14c]^ As electronic changes in the backbone ligand play an insignificant role in catalysis,^[20]^ this highlights the important role catalyst‐catalyst and catalyst‐solvent interactions play.

Two key studies highlight the importance of geometrical arrangements and steric influences on catalysis. Using mixed axial ligand systems of imidazole derivatives and the smaller DMSO ligand, less hindered coupling between the terminal oxygen atoms was observed.^[21]^ This is not entirely unexpected, as the catalysis is believed to proceed through an encounter complex of two monomeric units. This encounter complex, calculated by Sun and co‐workers, shows a highly interesting feature: the two monomeric units are not symmetrically placed with respect to each other, but their geometrical arrangement shows a displacement along the *y* axis (Figure 1 B).^[13]^

The aim of this study was to investigate if introducing a directed off‐set in the interaction of two monomeric Ru(bda)L_2_ units can positively influence oxygen‐oxygen bond formation in the encounter complex. The focus was placed on simple and easily accessible structural analogues of the well‐studied Ru(bda)(pic)_2_ (pic=4‐picoline). Instead of having a symmetrical axial ligand, bearing the modification of the pyridine ligand on the *para*‐position as is seen in most studies, we decided to move the substitution to the *meta*‐position (Figure 1 C). This de‐symmetrization of the axial ligands was hypothesized to create a natural cavity between the two monomeric catalysts by avoiding R‐R steric repulsion, minimizing energy requirements for the dimerization step by allowing space for the 7‐coordinated oxygen to undergo the reaction. As dictated by Concepcion and co‐workers, this high degree of pre‐organization was expected to reduce the main entropic contributor to the free energy of activation.^[14d]^ Following this simple theory, we report here enhanced water oxidation activities relative to earlier published Ru(bda)L_2_ analogues with *para*‐substituents. Furthermore, we show that similar respectable turnover frequencies can be achieved (31±3 s^−1^) at catalyst concentrations two orders of magnitude lower (1 μM) than required for the corresponding **Ru(bda)(pic)_2_**. Combined with computational results, we further support that the variation in catalytic efficiency is most likely a result of enhanced secondary interactions, confirming that direct electronic effects of the axial ligand are largely inferior in bimolecular water oxidation.

## Results and Discussion

**Figure 2:**
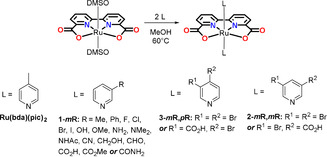
Preparation and the chemical structures of **Ru(bda)(pic)_2_** and 21 new catalysts used in this study.

Catalysts in this study were prepared according to slightly modified literature procedures. In short, a degassed mixture of Ru(bda)(DMSO)_2_ and the substituted pyridine ligand in methanol was heated to 60 °C. After overnight reaction the catalysts were obtained through flash column chromatography or simple filtration, in case the product displayed low solubility in methanol. Depending on the ligand substitution pattern, either **1‐*m*R** (*meta*‐substituted pyridines), **2‐*m*R**,***m*** **R** (doubly *meta*‐substituted pyridine), or **3‐*m*R**,***p*** **R** (*meta*‐ and *para*‐substituted pyridines) were obtained (Figure 2).

**Figure 3:**
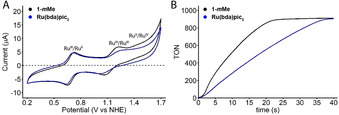
A) Cyclic voltammograms for **Ru(bda)(pic)_2_** and **1‐*m*Me**. B) Catalytic performances of **Ru(bda)(pic)_2_** and **1‐*m*Me**. [Ce^IV^]=0.365 M; [**Ru(bda)(pic)_2_**]=[**1‐*m*Me**]=100 μM, in pH 1 HNO_3_ in H_2_O containing 5 % CF_3_CH_2_OH. TOF_init_ was calculated by retrieving the maximum from a linear regression analysis connecting five measurement points. TON_max_ is limited by the oxidant to 912.5. The GC O_2_ yield based on Ce^IV^ is >95 % for both catalysts.

To probe the hypothesis, we designed the primary part of the study to involve the direct comparison of **Ru(bda)(pic)_2_** and its configurational isomer **1‐*m*Me**. Based on the Hammett σ‐constants, changing the position of the methyl group from the *para*‐ to the *meta*‐position incurs a reduction in its electron donating ability, changing the constant from −0.17 to −0.07. To investigate the influence of this change on the electrochemical behavior of the catalyst, differential pulse voltammetry (DPV, *cf*. ESI Figure S2B) and cyclic voltammetry (CV, Figure 3 A) were performed. It can easily be observed that indeed the oxidation potential of the Ru^III^/Ru^II^ and Ru^IV^/Ru^III^ couples show a slight anodic shift for **1‐*m*Me**, suggestive of an electronic influence of the axial ligands for water oxidation. An interesting observation that can be made from the CV and DPV is the clear intensity difference of the Ru^IV^/Ru^III^ and possibly the Ru^V^/Ru^IV^ oxidations, indicative of increased kinetic accessibility of the higher oxidation states for the **1‐*m*Me** catalyst.

In order to test if this suggestive electronic influence also translates to the actual oxygen evolution reaction, the chemical water oxidation catalyzed by **1‐*m*Me** was probed using Ce^IV^ as sacrificial oxidant. To ensure optimal solubility of all catalysts used in this study, prior to the kinetic measurements, the stock solution was treated with Ce^IV^ (40 equivalents, 10 TON equivalent for oxygen evolution) to convert all Ru^II^‐species to their respective Ru^III^‐form. This oxidation is easily discernible due to the loss of the characteristic red‐brown color of the stock solution of the Ru^II^(bda)L_2_ species and formation of a transparent yellow to colorless solution. The catalytic rates were confirmed to be independent of the pre‐treatment using samples where both Ru^II^‐ and Ru^III^‐form are soluble in the initial catalyst solution. Injection of **Ru(bda)(pic)_2_** or **1‐*m*Me** (100 μM final concentration) into a pH 1 solution of cerium ammonium nitrate (0.365 M Ce^IV^ in HNO_3_, TON_max_=912.5) provided pressure build‐up which could be tracked by a high‐precision pressure sensor. For both catalysts, full consumption of Ce^IV^ for the production of oxygen was achieved (Figure 3 B). From the kinetic trace it can easily be discerned that oxygen evolution using **1‐*m*Me** is considerably faster, for which a TOF_init_ of 87 s^−1^ was obtained versus the much lower 35 s^−1^ observed for **Ru(bda)(pic)_2_** under the same conditions. From absorption analysis of the re‐reduced catalysts catalyst decomposition was nearly identical for both **1‐*m*Me** and **Ru(bda)(pic)_2_**, which is marginal compared to the 2.5‐fold difference in TOF_init_ ruling out the influence of catalyst decomposition on the activity differences (Figure S15–S16). Similarly, controlled potential electrolysis shows no significant difference in decomposition between **1‐*m*Me** and **Ru(bda)(pic)_2_** (Figure S6). Although the electronic effect dictates that thermodynamics are less ideal for water oxidation on **1‐*m*Me**, the pressure sensor data suggests that moving the methyl substituent to the *meta*‐position instigates a reduced kinetic barrier for the crucial O‐O coupling step.

To confirm if electronic effects arising from the coordinated axial ligand play a clear role in the kinetics of water oxidation, a series of seventeen catalysts (**1‐*m*R**, Figure 2) was prepared exhibiting changes in the electron‐withdrawing or ‐donating properties of the *meta*‐substituent on the axial pyridyl‐ligand (Hammett σ_m_ from −0.07 to 0.56).^[22]^ In addition, this catalyst series allowed for probing of other secondary effects, such as π‐system extension, hydrophobic effects and the previously studied halogen‐π interactions.^[14d]^ It is worth to note that **1‐*m*OH** could only be obtained with a third 3‐hydroxypyridine ligand coordinated to the equatorial plane of the catalyst, and that the electron‐donating ability of **1‐*m*NH_2_** and **1‐*m*NMe_2_** may be affected by the acidic conditions used in the measurements. Initial studies by CV clearly show a dependence of the Ru^III^/Ru^II^ wave on the substituent used in the studies (*cf*. ESI, Figure S1), with the most electron‐poor **1‐*m*CN** displaying the largest shift towards anodic potentials. From the CV it can clearly be discerned that **1‐*m*Ph** displays larger currents for all oxidation peaks, an effect potentially caused by the stronger interaction of the hydrophobic phenyl groups and the glassy carbon electrode. Interestingly, much larger currents are observed for **1‐*m*NMe_2_** suggestive of potential catalytic superiority over other catalysts in this series. However, further investigation showed that this catalyst decomposes under electrocatalytic conditions.

**Figure 4:**
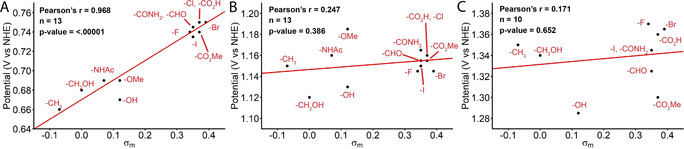
Pearson's correlation analysis of the catalysts redox couples: A) Ru^III^/Ru^II^, B) Ru^IV^/Ru^III^, and C) Ru^V^/Ru^IV^ with the Hammett‐values of the substituent. Four samples were removed for the correlation analysis: **1‐*m*NH_2_**, **1‐*m*NMe_2_** (for potential inaccuracy in σ_m_ due to protonation possibility) and **1‐*m*CN** and **1‐*m*Ph** (after outlier analysis by plotting e_i_/S_V,σ_ for Ru^III^/Ru^II^ over σ_m_, Figure S3).

The oxidation potentials of all redox couples were deducted from DPV curves (*cf*. ESI, Figure S2 and Table S1). Interestingly, a good Pearson's r value could be obtained correlating σ_m_ with the Ru^III^/Ru^II^ oxidation, as previously also observed by Murata and co‐workers for catalysts bearing a *para*‐substituted pyridine.^[12]^ As the Ru^II^‐species is not involved in the catalytic cycle, the two other redox couples were investigated as well, however, no clear correlation could be observed between their oxidation potentials and σ_m_ (Figure 4). These results suggest that σ_m_ of substituents can be predicted with reasonable accuracy from the Ru^III^/Ru^II^ redox couple through coordination of the substituted pyridyl analogue to the Ru(bda) center. More importantly, the results indicate an electronic effect on the initial oxidation to convert the catalyst precursor to its active Ru^III^‐analogue, placing it on the catalytic cycle, however, no dependence was observed between σ_m_ and the oxidations involved in the catalytic turnover.

Moving the substituent from the *para*‐ (σ_p_=−0.17) to the *meta*‐position (σ_m_=−0.07) of the methyl substituent, i.e., it becomes relatively less electron donating. To further investigate if this electronic effect has an obvious influence on the kinetics of the oxygen evolution, all seventeen catalysts were measured under the same conditions and the TOFs were compared (Table 1, Figure S7–S8). Very low TOF values were obtained for **1‐*m*NH_2_**, **1‐*m*NMe_2_** and **1‐*m*OH** due to their instability under the conditions used for the measurement. This oxidative instability is also reflected by the lack of a well‐defined reversible Ru^IV^/Ru^III^ wave in their CVs (Figure S1A,C). Low concentration DPV revealed that the instability of these catalysts can be a result of oxidation occurring at the ligand instead, as apparent by the observation of additional redox peaks (Figure S4). For **1‐*m*Ph** a decent TOF_init_ was observed of 73 s^−1^, however, the reaction kinetics quickly reverted to 1^st^ order behavior and a TOF of ≈1 s^−1^. **1‐*m*Ph** was the only catalyst displaying this interesting behavior, which is potentially caused by too strong intermolecular interactions or hydrophobic effects causing aggregation of the catalyst.

**Table 1:** Chemical oxygen evolution rates of the catalysts used in this study ranked with respect to their Hammett constant.

Catalyst	σ_m_	TOF_init_ (s^−1^)^[a]^
**1‐*m*NH_2_**	−0.16^[b]^	5.2^[c]^
**1‐*m*NMe_2_**	−0.15^[b]^	10.6^[c]^
**1‐*m*Me**	−0.07	86.9
**1‐*m*CH_2_OH** ^[d]^	0	43.5
**1‐*m*Ph**	0.06	72.7
**1‐*m*NHAc**	0.07	127.5
**1‐*m*OH**	0.12	2.0^[c]^
**1‐*m*OMe**	0.12	45.0
**1‐*m*F**	0.34	53.8
**1‐*m*I**	0.35	412.1
**1‐*m*CHO**	0.35	67.7
**1‐*m*CONH_2_**	0.35^[e]^	84.9
**1‐*m*Cl**	0.37	206.9
**1‐*m*CO_2_H**	0.37	89.2
**1‐*m*CO_2_Me**	0.37	166.6
**1‐*m*Br**	0.39	330.7
**1‐*m*CN**	0.56	118.2

[a] Catalytic performances of **1‐*m*R**. [Ce^IV^]=0.365 M; [**1‐*m*R**]=100 μM, in pH 1 HNO_3_ in H_2_O containing 5 % CF_3_CH_2_OH. TOF_init_ was calculated by retrieving the maximum from a linear regression analysis connecting five measurement points. TON_max_ is limited by the oxidant to 912.5. [b] The σ_m_‐value displayed is for the non‐protonated species, the σ_m_‐value of the ammonium groups would be approximately 0.86. [c] Rapid decomposition of the catalyst is observed and as a result TON_max_ is not achieved. [d] Please note that Ce^IV^ is known to be capable of oxidizing aliphatic alcohols. [e] The displayed σ_m_‐value is for the ‐CONHMe substituted aryl group, most closely related to the substituent used for **1‐*m*CONH_2_** for which no Hammett data was available.^[22]^

Overall, no trend could be observed between the Hammett constants and the reaction kinetics, with both electron‐rich and poor substituents displaying independent TOF_init_ values varying from decent to good.

These results clearly confirm that electronic effects on the axial ligand play a minimal role in determining the reaction kinetics in the cerium(IV)‐driven chemical water oxidation reaction. The reaction thus appears to be accelerated by increased intermolecular interactions and proper alignment of the catalytic units during catalysis. For example, changing from the hydrophilic carboxylic acid based catalyst (**1‐*m*CO_2_H**) to its more hydrophobic methyl ester derivative (**1‐*m*CO_2_Me**) coincides with a two‐fold increase in TOF_init_ from 89 to 167 s^−1^, respectively. Interestingly, similar effects for the halide series as observed by Concepcion and co‐workers^[14d]^ are found in this series of catalysts, albeit with increased performance for the catalysts reported here with the substituent in the *meta*‐position. In comparison, the previously published **1‐*p*Cl**, **1‐*p*Br** and **1‐*p*I** have a reported TOF_max_ of 62, 101 and 334 s^−1^, respectively, versus the higher un‐optimized TOF_init_ observed for **1‐*m*Cl**, **1‐*m*Br** and **1‐*m*I** of 207, 331, and 412 s^−1^, respectively. Overall, this confirms the importance of the previously described halogen‐π interactions and further supports the importance of directing the interaction between the two separate catalytic units in minimizing the reaction barrier, where introduction of a slight off‐set in the interaction favors catalytic turnover.

**Figure 5:**
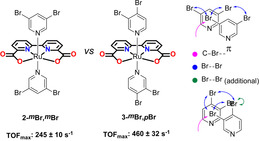
Structures of **2‐*m*Br**,***m*** **Br** and **3‐*m*Br**,***p*** **Br** and schematic of the most important interactions involved in compressing two catalytic units together.

**Figure 6:**
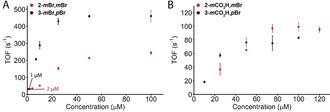
Catalytic performances of A) **2‐*m*Br**,***m*** **Br** and **3‐*m*Br**,***p*** **Br** and B) **2‐*m*CO_2_H**,***m*** **Br** and **3‐*m*CO_2_H**,***p*** **Br** at different catalyst concentrations. [Ce^IV^]=0.365 M in pH 1 HNO_3_ in H_2_O containing 5 % CF_3_CH_2_OH. TOFs were calculated by retrieving the maximum from a linear regression analysis connecting a minimum of five measurement points. Due to the low response observed for [**2‐*m*Br,*m*Br**]=1 μM, the TOF was calculated from the mid‐part of the oxygen evolution curve, where the highest degree of linearity in the measurement points was observed.

To further investigate the hypothesis, that introduction of slight asymmetry in the interaction plays a crucial role in promoting the O−O bond formation step, disubstituted pyridyl ligands were targeted next. Based on availability of the ligands and solubility of the final compounds, **1‐*m*Br** derived systems were targeted instead of derivatives of the previously observed better catalyst **1‐*m*I**. Two dibromopyridine based catalysts were prepared, bearing the bromine‐substitution in both *meta*‐positions (**2‐*m*Br**,***m*** **Br**) and bearing the bromine‐substitution on the *meta*‐ and *para*‐position (**3‐*m*Br**,***p*** **Br**). For **2‐*m*Br**,***m*** **Br** symmetry of the catalyst is re‐established, whereas for **3‐*m*Br**,***p*** **Br** the asymmetry in the catalyst is maintained, maximally supporting the ideal pre‐organization in the encounter complex (Figure 5). To get a better impression of the effect of the substitution pattern on the catalysis, oxygen evolution was measured at different concentrations (*cf*. ESI Figure S5 for CV and DPV and Figure S9–S10 for chemical oxygen evolution performance). As expected, **3‐*m*Br**,***p*** **Br** showed better TOFs_init_ than **2‐*m*Br**,***m*** **Br**, however, the effect was dramatically more pronounced than initially anticipated (Figure 6 A). For **3‐*m*Br**,***p*** **Br** an increased TOF_max_ of 460±32 s^−1^ was observed, whereas the TOF_max_ for symmetric **2‐*m*Br**,***m*** **Br** lingered at 245±10 s^−1^, even lower than observed for mono‐substituted **1‐*m*Br**. In case the bromine‐substituents had provided a linear cumulative effect, it would have been expected that **2‐*m*Br**,***m*** **Br** would have been the stronger catalyst instead (TOF_max_ of 101 s^−1^ for **1‐*p*Br**, and TOF_init_ of 331 s^−1^ for **1‐*m*Br**).

Another aspect indicative of the reduced kinetic barrier in the encounter complex is the concentration where the rate‐determining step shifts from radical coupling controlled kinetics (2^nd^ order behavior) to oxidation controlled kinetics (1^st^ order behavior). For symmetric **2‐*m*Br**,***m*** **Br** the rate‐determining step changes at ≈50 μM catalyst concentration, whereas **3‐*m*Br**,***p*** **Br** is less dependent on catalyst concentration showing the shift to 2^nd^ order dependence on the catalyst only at concentrations lower than ≈25 μM (Figure S13). As a result, **3‐*m*Br**,***p*** **Br** exhibits a reasonable TOF_init_ of 31±3 s^−1^ even at concentrations as low as 1 μM. Placed in perspective, this is about 2 orders of magnitude lower in concentration than required for **Ru(bda)(pic)_2_** for achieving the same TOFs. In addition, a TON_max_ of 12 500 is observed for **3‐*m*Br**,***p*** **Br** making this the most stable Ru(bda)L_2_ catalyst with axial pyridyl substituents up to date.

The superiority of **3‐*m*Br**,***p*** **Br** is also apparent from its ability to catalyze water oxidation through the I2M pathway even at low concentrations, whereas water oxidation with **2‐*m*Br**,***m*** **Br** starts proceeding through the WNA pathway at concentrations below 10 μM. The symmetric **2‐*m*Br**,***m*** **Br** still displayed clear catalytic behavior at 2 μM catalyst concentration, however, lost its performance at lower concentrations. With a clear vision of the catalytic activity of **3‐*m*Br**,***p*** **Br** at 1 μM, we investigated if the reaction was dependent on [Ce^IV^] (Figure S14). The observed TOFs at [Ce^IV^]=5, 20, and 100 mM were 29.9±4.6, 29.1±5.9, and 26.7±6.3 s^−1^, respectively. This provides further evidence that the rate limiting step at this low concentration of **3‐*m*Br**,***p*** **Br** is still the bimolecular step, which does not involve the sacrificial oxidant.

To explore if similar effects are apparent for less ideal systems, the combination of less favored ‐CO_2_H (TOF_init_ **1‐*m*CO_2_H**=89 s^−1^) and highly favored ‐Br substituents was investigated (Figure 6 B, *cf*. ESI Figure S5 for electrochemistry and Figure S11–12 for chemical oxygen evolution performance). If the catalytic rate is mainly governed by the favored ‐Br substituent and not the off‐set interaction, catalytic rates are expected to be high for the mixed system with the bromine in the *meta*‐position. However, when testing **2‐*m*CO_2_H**,***m*** **Br** under chemical oxygen evolution conditions a significant drop in catalytic activity (TOF_max_=99±6) was found as compared to **1‐*m*Br**. For **3‐*m*CO_2_H**,***p*** **Br**, with the bromine substituent in the less privileged *para*‐position, only a slightly lower TOF_max_ was observed. Most remarkably, also for this less ideal system clear differences can be observed in the point where catalysis starts showing first order dependence on the catalyst concentration (Figure S13). Herein, the catalyst favoring off‐set bimolecular interactivity **3‐*m*CO_2_H**,***p*** **Br** maintains first order dependence longer with decreasing concentrations, eventually outcompeting **2‐*m*CO_2_H**,***m*** **Br**, which performs better at higher concentrations.

Although thorough computational studies by Ahlquist and co‐workers suggested that the kinetics of the bimolecular step is defined by the formation of prereactive dimer rather than the transition state, it was concluded that noncovalent interactions between axial ligands of two M=O units is the key factor contributing to the rate of radical coupling.^[23]^ Concepcion and co‐workers demonstrated that π‐π interactions play a key role in case of isoquinoline, while X‐π interactions are defining in pyridines (X—*para*‐substituent).^[14d]^ In order to gain more insight into the role of off‐set and explain the superior performance of the catalysts featuring meta‐substituted pyridines, we assessed the interactions between ligands in the radical coupling product–peroxo‐dimer. Despite the structure of peroxo‐dimer does not directly reflect the kinetics of the coupling step, it provides important information about the role and nature of axial ligands interactions.

**Figure 7:**
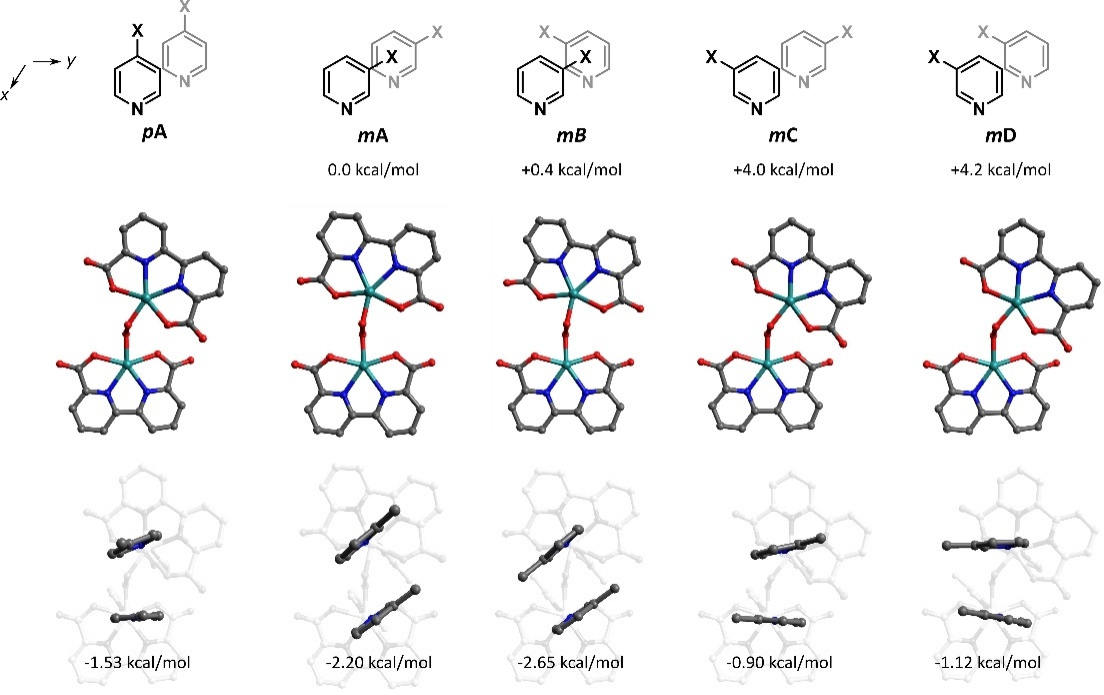
Optimized structures of peroxo‐dimers of **Ru(bda)(pic)_2_** (**pA**, X=Me) and **1‐*m*Me** (***mA*** **‐D**, capital letters indicate different relative configuration of interacting axial ligands), relative Gibbs formation energies (for ***mA*** **‐D**), and complexation energies for interacting axial ligands (average of two pairs, negative value implies attraction). Ru turquoise, O red, N blue, C grey.

Since a significant difference in catalytic activity was observed even between 3‐picoline and 4‐picoline, for which halogen‐π interactions are absent, we used **1‐*m*Me** and **Ru(bda)(pic)_2_** as a model for comparison. As 3‐picoline is less symmetric, four different configurations exist for the dimer (A–D), all of which have been explored (Figure 7). Among 4 configurations, two (***mA*** and ***mB***) were found to be preferential by about 4 kcal mol^−1^, suggesting they would be primarily encountered in the O‐O coupling step. Interestingly, there is a distinct difference between ***mA‐B*** and both ***mC‐D*** and ***pA***: the angle between front faces of bda ligands exceeds 40° in the latter, consistent with previous results for ***pA***, and is much smaller (≈15°) in the former—a feature only observed in isoquinoline derivatives (Figure 7, Table S3).^[14d]^ The prevalence of the ‘skewed’ conformation in ***pA*** but not in ***mA‐B*** suggests that rotation of bda units around the O−O bond is energetically dominated by the interactions between axial ligands. To confirm this, we extracted axial ligands from the optimal geometries and calculated the complexation energies between each pair of interacting ligands.

It was found that, indeed, a significant part of the energy difference between ***mA‐D*** could be attributed to the difference in axial ligands interactions. In ***pA***, ***mC*** and ***mD*** geometries, relative dispositions of the axial ligands are very similar, with 15–20° angle between aromatic rings. This indicates that Ru‐Ru distance in peroxo‐dimer (and, subsequently, prereactive dimer) is too large to take full advantage of π‐π interactions. In contrast, the axial ligands in ***mA*** and ***mB*** are positioned in parallel planes, minimizing the X‐π distance, which is the most essential contribution to the stacking.^[24]^ As *meta*‐substituent is located over the center of another pyridine ring, this interaction should be present in structurally close complexes, such as the prereactive dimer, affecting the ligand binding energy and thus increasing collision rate. Moreover, positioning of the meta‐substituent close to another pyridine ring can positively contribute to the hydrophobic attraction, effectively reducing the solvent accessible surface area (Table S3). These observations confirm the previously proposed X‐π interactions as determining for the bimolecular coupling step and rationalize superior performance of catalysts in this work, compared to their *para*‐substituted analogs. Furthermore, these results demonstrate that commonly overlooked methyl‐π interactions can lead to a significant enhancement in catalysis, provided a proper geometric alignment, such as in the proposed **1‐*m*Me** dimer.^[4b]^

## Conclusion

This work provides a new strategy for overcoming the limitations of 2^nd^ order catalysis, through simple structural considerations. By de‐symmetrization of the axial pyridine ligands in the Ru(bda)L_2_ type catalysts, moving the substituent from *para‐* to *meta*‐position, improved chemical oxygen evolution efficiencies have been achieved. No apparent correlation between the σ_m_ of the substituent, TOF and redox potentials further excludes electronic influence of the axial ligand on catalysis. Record efficiencies with a TOF of 460±32 s^−1^ and TON of 12 500 are observed for the most efficient 3,4‐dibromopyridine substituted catalyst **3‐*m*Br**,***p*** **Br**. In addition, longer maintenance of the 1^st^ order dependence on the catalyst concentration provides **3‐*m*Br,*p*Br** with a TOF of 31±3 s^−1^ at a concentration of only 1 μM, a similar TOF as observed for **Ru(bda)(pic)_2_** at two‐orders higher concentrations. We hypothesize that the introduced off‐set in the bimolecular interaction provides ample space for the O−O bond formation to occur, reducing its barrier. Similar off‐set interactions were proposed for **Ru(bda)(isoq)_2_** catalysts with its extended π‐system, however, DFT results suggest that the Ru‐Ru distance in the prereactive dimer is too large to optimally benefit from direct π‐π interactions between pyridine ligands, and position of the pyridine substituents is crucial for the stacking. Overall, this work shows that de‐symmetrization is the key factor governing the catalytic activity, offering inspiration for future design of water oxidation catalysts. The presented results may also serve to inspire investigation on other types of cooperative catalysis, such as N_2_ and CO_2_ de‐symmetrization.

## Conflict of interest

The authors declare no conflict of interest.

## Supporting information

As a service to our authors and readers, this journal provides supporting information supplied by the authors. Such materials are peer reviewed and may be re‐organized for online delivery, but are not copy‐edited or typeset. Technical support issues arising from supporting information (other than missing files) should be addressed to the authors.

Supplementary
