# Supplementary material for: Off‐Set Interactions of Ruthenium–bda Type Catalysts for Promoting Water‐Splitting Performance
Source: Angew Chem Int Ed Engl. 2021 May 19;60(26):14504–11. doi: 10.1002/anie.202101931 (PMC8251529; doi:10.1002/anie.202101931)
Supplement: Supplementary file 1 — Supplementary [file ANIE-60-14504-s001.pdf]

Angewandte Chemie
Eine Zeitschrift der Gesellschaft Deutscher Chemiker

Supporting Information

Off-Set Interactions of Ruthenium–bda Type Catalysts for Promoting Water-Splitting Performance

*Brian J. J. Timmer, Oleksandr Kravchenko, Tianqi Liu, Biaobiao Zhang, and Licheng Sun**

anie_202101931_sm_miscellaneous_information.pdf

Catalyst Preparation & Characterization

General Procedures

All chemicals were purchased from commercial suppliers and used as received. Solvents used during workup, extraction and flash column chromatography were analytical or HPLC grade and used as supplied. Reactions were monitored by thin-layer chromatography (TLC) using pre-coated Merck silica gel 60-F254 alumina plates (0.25 mm). Visualization of unreacted ligand was performed using ultraviolet light (λ = 254 nm). Flash chromatography was carried out using Merck silica gel 60 (particle size 0.040–0.063 mm). ATR-IR spectroscopy was performed on a Thermo Scientific Nicolet iS10 spectrophotometer. Mass spectrometry was performed at the Dalian University of Technology (China). NMR spectra (^{1}H) were collected with a Bruker Ascend 400 NMR spectrometer. Chemical shifts are reported as δ-values (ppm) relative to tetramethylsilane with (residual) solvent as internal standard. All coupling constants (J) are given in Hertz (Hz). Decomposition temperatures (T_d) were measured through thermogravimetric analysis with a heating rate of 10°C per minute under a N_2 flow of 30 mL per minute. The TGA plots provided show the 1st derivative in red for illustration purposes. The first minimum was set as the decomposition temperature. The reference decomposition temperature for **Ru(bda)(pic)$_2$** is 338 °C. UV/Vis spectroscopy was performed in cuvettes with a 1mm path length using CF_3CH_2OH as solvent and corrected for the solvent absorption. The reference molar extinction coefficient for **Ru(bda)(pic)$_2$** is ε_{349} = 8098 $M^{-1}\cdot cm^{-1}$.

General Preparation

A suspension of Ru(bda)(DMSO)$_2$ (0.1 mmol, 50 mg) and solid pyridine ligands in MeOH (5 mL) was degassed by bubbling with nitrogen for a minimum of 15 minutes (for liquid pyridines the ligand was added after degassing). The mixture was heated to 60°C overnight. After cooling down the product was purified using either filtration or flash column chromatography. The product was thoroughly dried *in vacuo* prior to electrochemical and kinetic experiments.

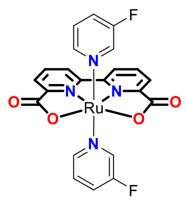

1-*m*F: Pyridine ligand = 3-fluoropyridine (10 eq., 1 mmol, 97 mg, 86 µL). After 16h reaction time, the mixture was concentrated in vacuo and applied on a column of silica gel. The product was eluted using 5% MeOH in CH_2Cl_2 to 10% MeOH in CH_2Cl_2 as eluent. Fractions containing the product were concentrated in vacuo. The product was re-suspended in EtOAc, filtered and washed with Et_2O to obtain the product as a red-brown solid (29.4 mg, 55 µmol, 55%). **^{1}H NMR** (400 MHz, DMSO) δ 8.72 (dd, *J* = 7.0, 1.5 Hz, 2H), 7.97 – 7.86 (m, 6H), 7.73 (dt, *J* = 8.2, 2.2 Hz, 2H), 7.47 (d, *J* = 5.5 Hz, 2H), 7.33 (dt, *J* = 8.4, 5.7 Hz, 2H). **HRMS:** (TOF-MS ES, m/z) calc. for $C_{22}H_{14}F_2N_4O_4RuH$ $[M+H]^+$: 539.0099, found: 539.0116. **T_d**: 290 °C. **IR** (cm^{-1}) 3111, 3062, 1739, 1635, 1607, 1579, 1477, 1435, 1409, 1367, 1289, 1251, 1236, 1187, 1171, 1149, 1139, 1104, 1042, 1033, 1022, 941, 904, 875, 844. **UV/Vis:** ε_{373} = 10660 $M^{-1}\cdot cm^{-1}$.

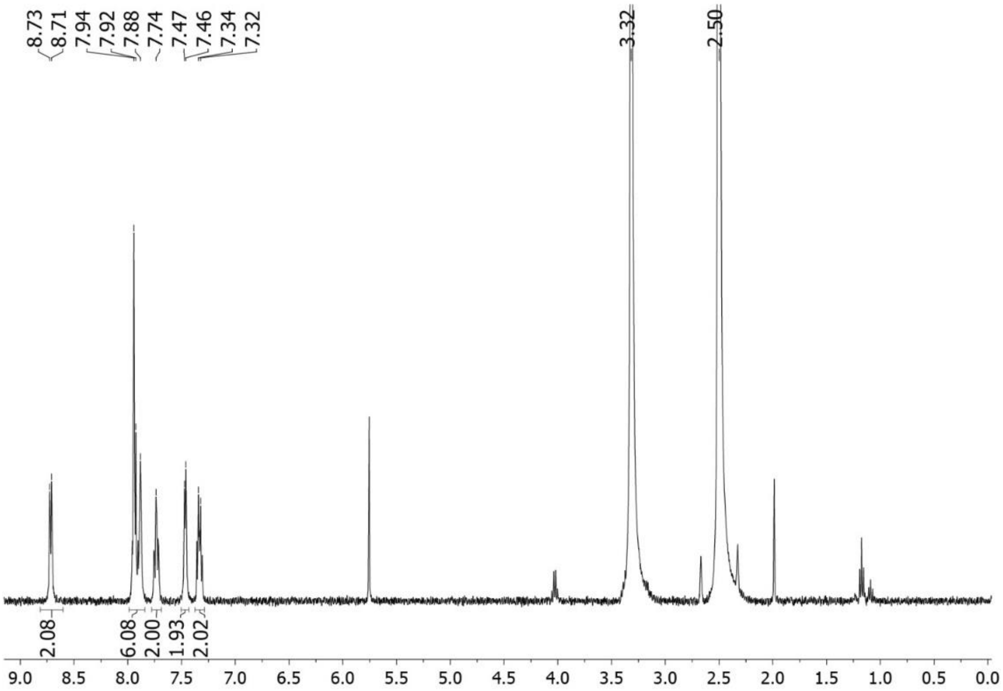

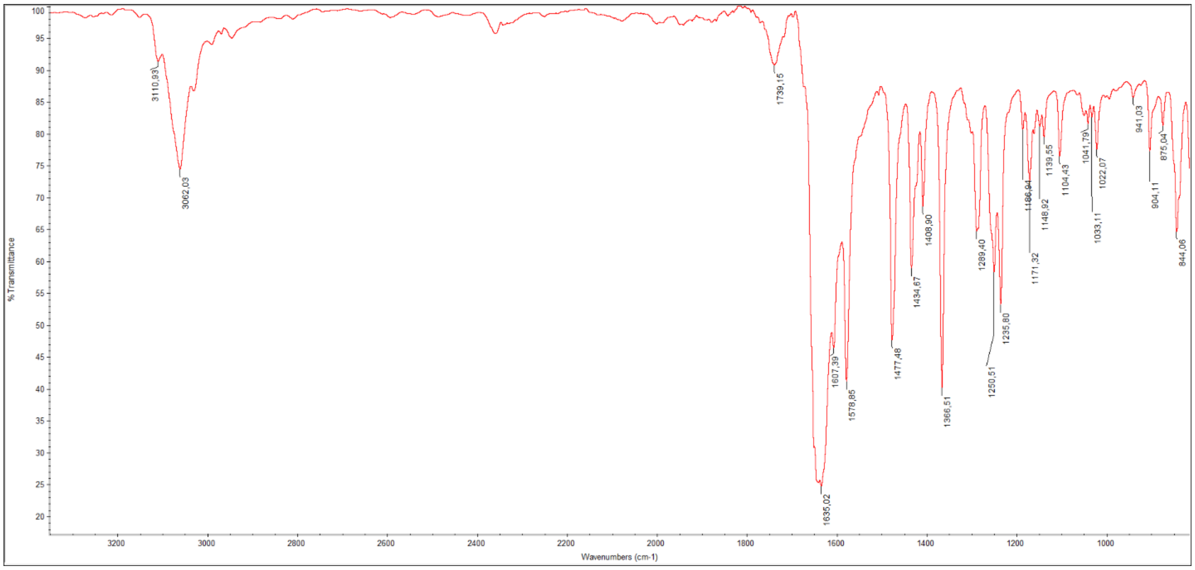

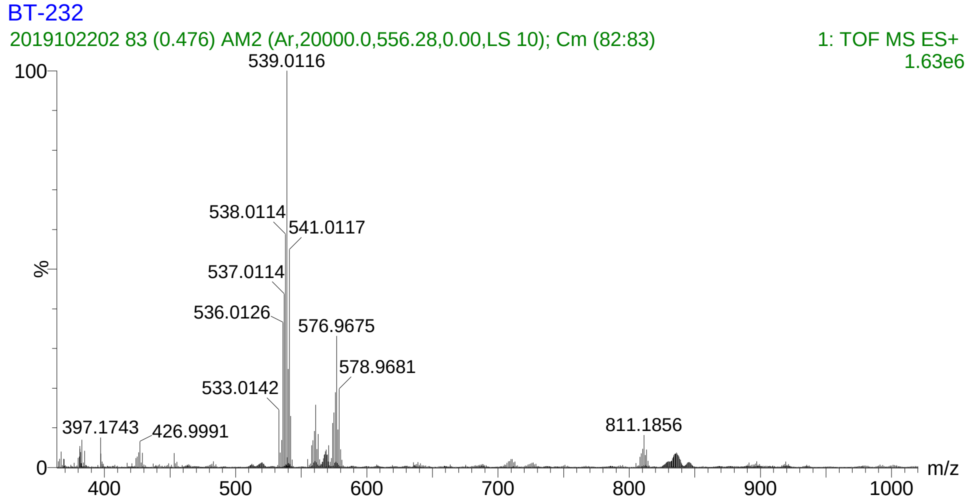
BT-232
2019102202 83 (0.476) AM2 (Ar,20000.0,556.28,0.00,LS 10); Cm (82:83)
1: TOF MS ES+
1.63e6
539.0116
538.0114
541.0117
537.0114
536.0126
576.9675
578.9681
533.0142
397.1743
426.9991
811.1856
100
%
0
400
500
600
700
800
900
1000
m/z

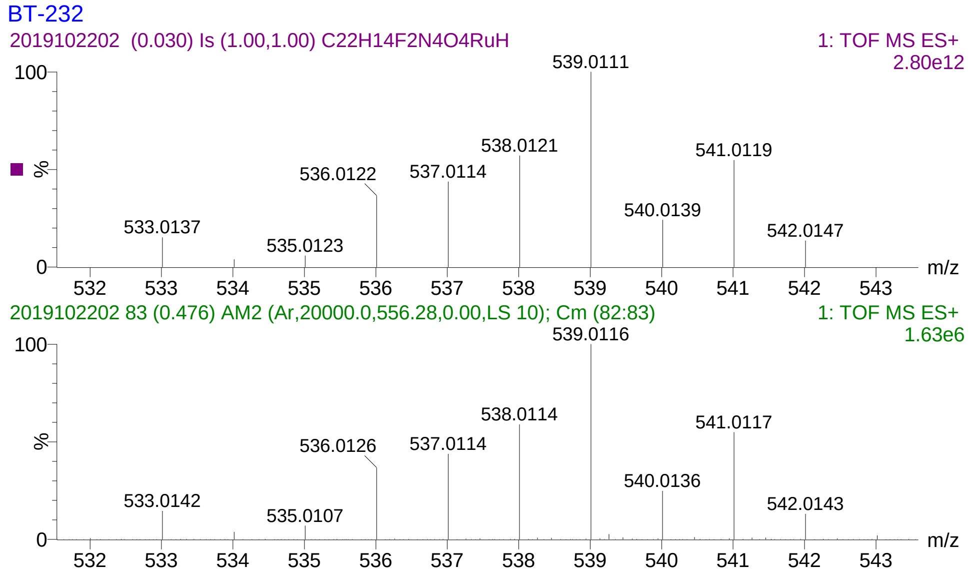
BT-232
2019102202 (0.030) Is (1.00,1.00) C22H14F2N4O4RuH
1: TOF MS ES+
2.80e12
539.0111
538.0121
541.0119
536.0122
537.0114
540.0139
533.0137
542.0147
535.0123
2019102202 83 (0.476) AM2 (Ar,20000.0,556.28,0.00,LS 10); Cm (82:83)
1: TOF MS ES+
1.63e6
539.0116
538.0114
541.0117
536.0126
537.0114
540.0136
533.0142
542.0143
535.0107
100
%
0
532
533
534
535
536
537
538
539
540
541
542
543
m/z

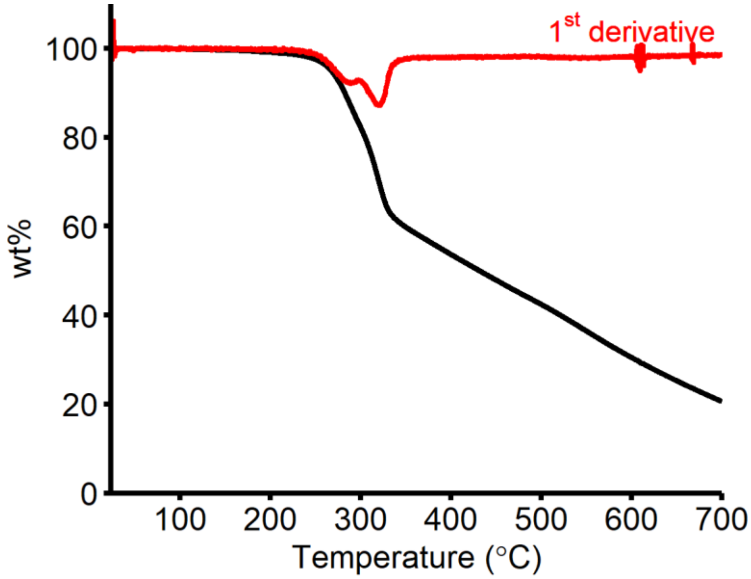
1st derivative
wt%
Temperature (°C)
0
20
40
60
80
100
100
200
300
400
500
600
700

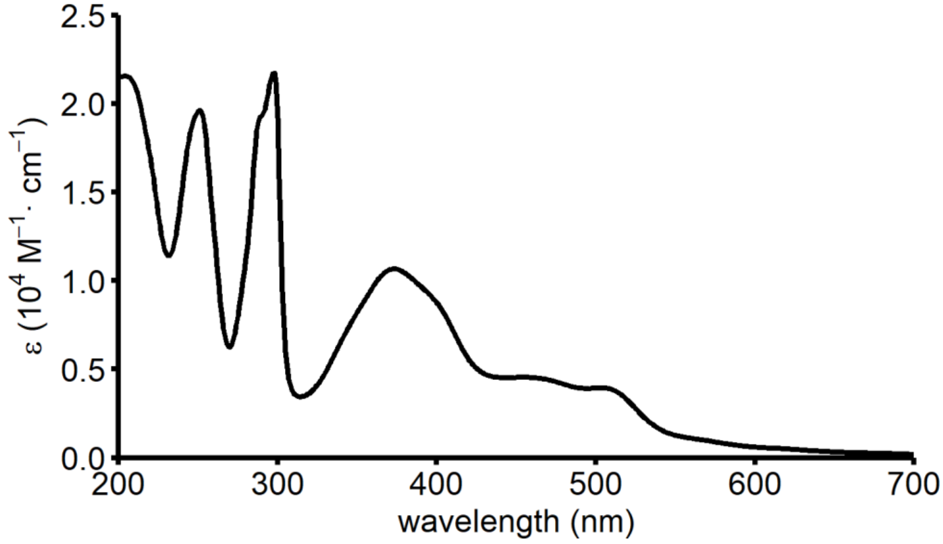

ε (10^4 M^{-1}· cm^{-1})
2.5
2.0
1.5
1.0
0.5
0.0
200
300
400
500
600
700
wavelength (nm)

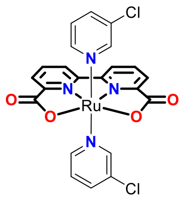

1-*m*Cl: Pyridine ligand = 3-chloropyridine (10 eq., 1 mmol, 114 mg, 95 µL). After 16h reaction time, the mixture was concentrated in vacuo and applied on a column of silica gel. The product was eluted using 5% MeOH in CH_2Cl_2 to 10% MeOH in CH_2Cl_2 as eluent. Fractions containing the product were concentrated in vacuo to obtain the product as a red-brown solid (36.2 mg, 63 µmol, 63%). **^{1}H NMR** (400 MHz, DMSO) δ 8.74 (dd, *J* = 6.6, 2.3 Hz, 2H), 8.04 (d, *J* = 1.6 Hz, 2H), 7.92 (dd, *J* = 16.1, 7.9 Hz, 6H), 7.39 (d, *J* = 5.3 Hz, 2H), 7.26 (dd, *J* = 8.1, 5.7 Hz, 2H). **HRMS:** (TOF-MS ES, m/z) calc. for $C_{22}H_{14}Cl_2N_4O_4RuH$ $[M+H]^+$: 570.9508, found: 570.9509. **T*d***: 311 °C. **IR** (cm^{-1}) 3071, 3043, 2931, 1635, 1588, 1554, 1464, 1419, 1400, 1361, 1285, 1253, 1166, 1148, 1116, 1104, 1038, 1026, 904, 835. **UV/Vis:** ε_{378} = 11745 $M^{-1}\cdot cm^{-1}$.

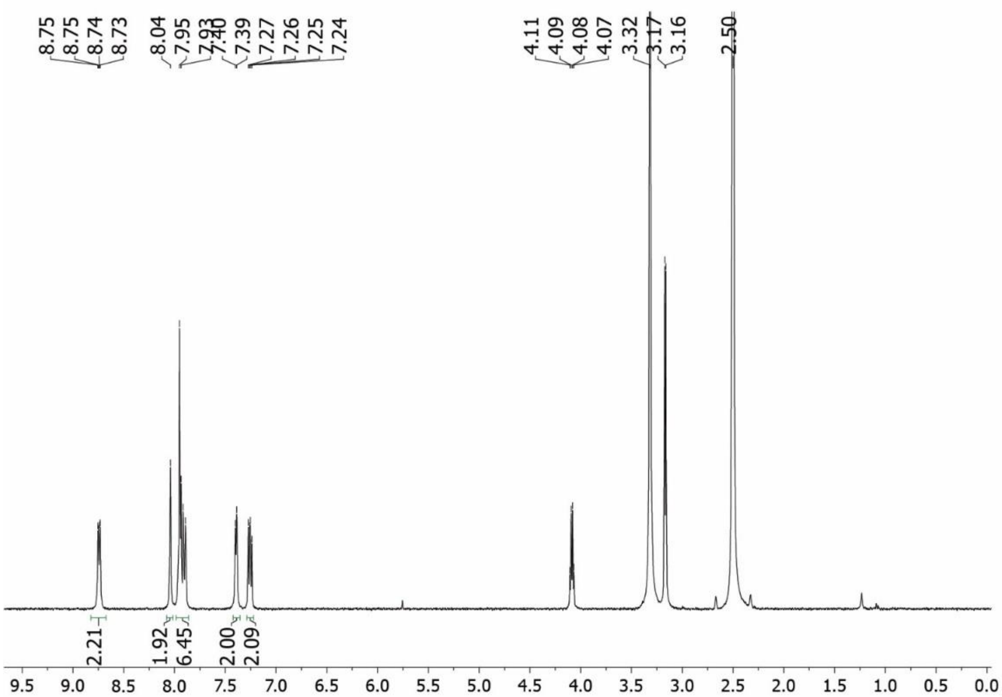

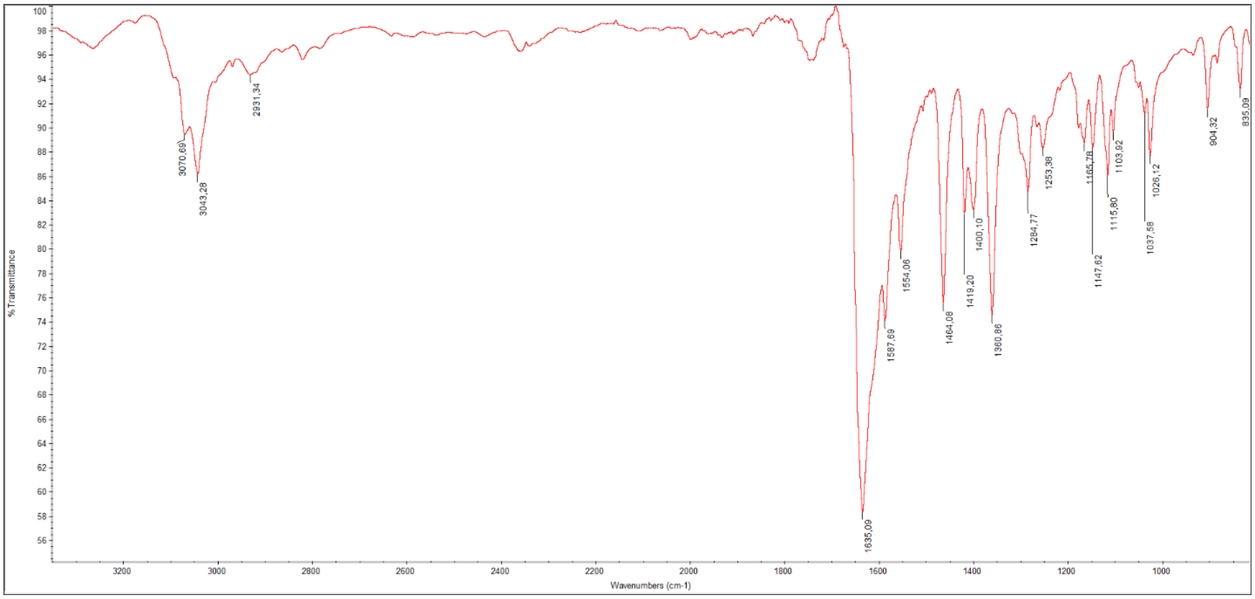

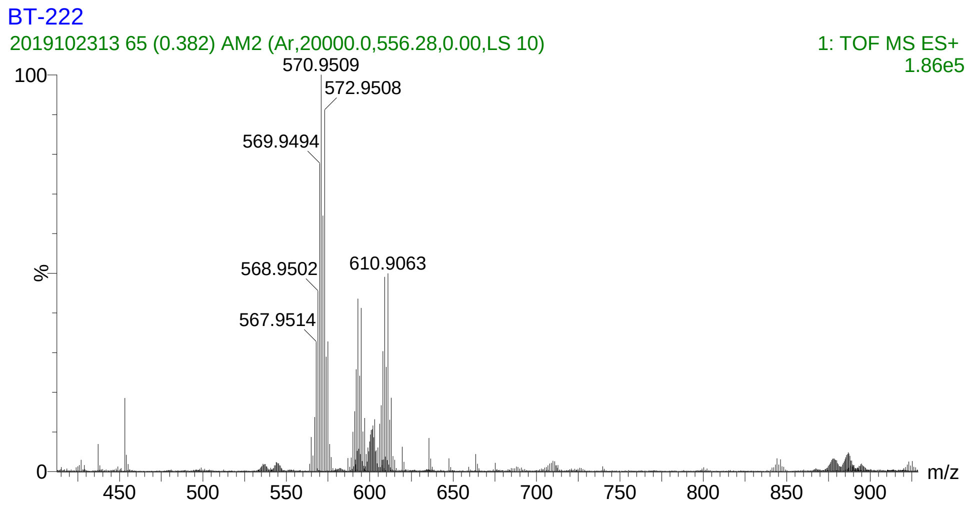
BT-222
2019102313 65 (0.382) AM2 (Ar,20000.0,556.28,0.00,LS 10)
1: TOF MS ES+
1.86e5
570.9509
572.9508
569.9494
568.9502
610.9063
567.9514
100
%
0
450
500
550
600
650
700
750
800
850
900
m/z

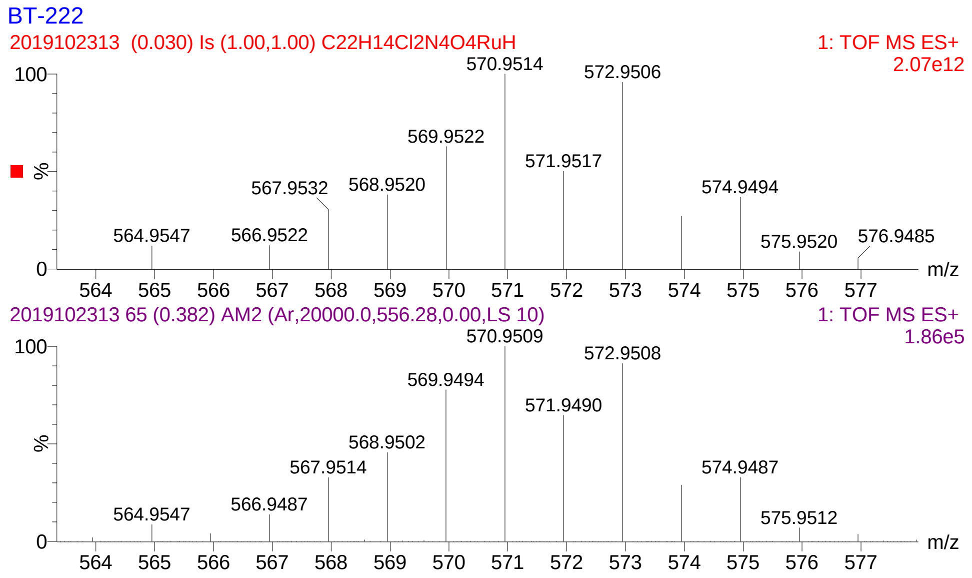
BT-222
2019102313 (0.030) Is (1.00,1.00) C22H14Cl2N4O4RuH
1: TOF MS ES+
2.07e12
570.9514
572.9506
569.9522
571.9517
567.9532
568.9520
574.9494
564.9547
566.9522
575.9520
576.9485
2019102313 65 (0.382) AM2 (Ar,20000.0,556.28,0.00,LS 10)
1: TOF MS ES+
1.86e5
570.9509
572.9508
569.9494
571.9490
568.9502
567.9514
574.9487
564.9547
566.9487
575.9512
100
%
0
564
565
566
567
568
569
570
571
572
573
574
575
576
577
m/z

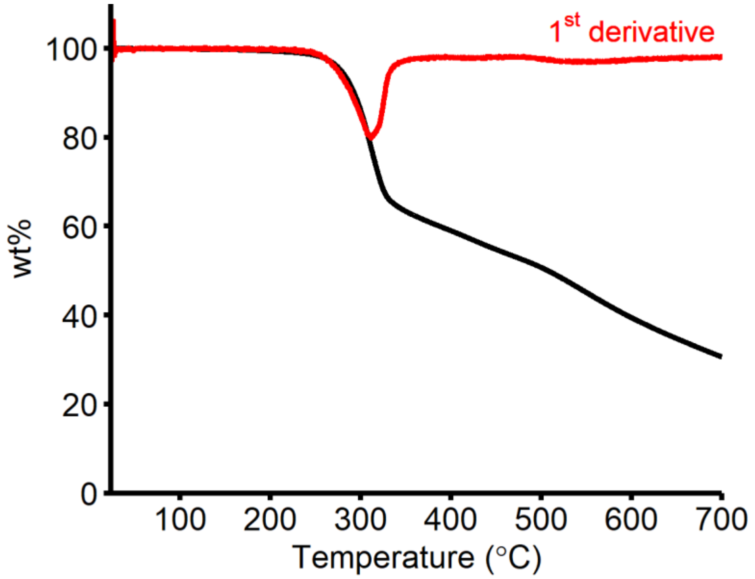
1st derivative
wt%
100
80
60
40
20
0
100
200
300
400
500
600
700
Temperature (°C)

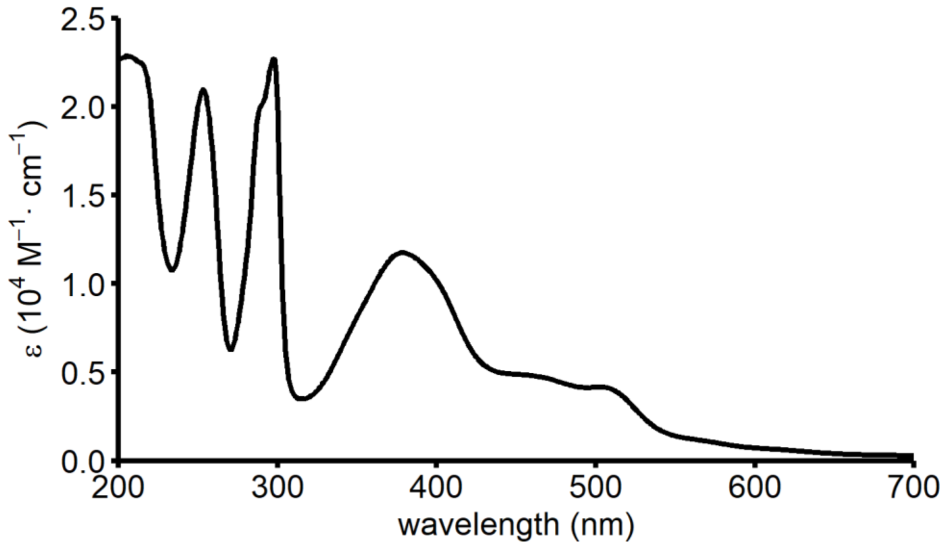
2.5
2.0
1.5
1.0
0.5
0.0
ε (10^4 M^-1 · cm^-1)
200
300
400
500
600
700
wavelength (nm)

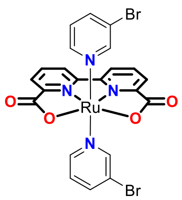

1-*m*Br: Pyridine ligand = 3-bromopyridine (10 eq., 1 mmol, 158 mg, 96 µL). After 16h reaction time, the mixture was concentrated in vacuo and applied on a column of silica gel. The product was eluted using 5% MeOH in CH_2Cl_2 to 10% MeOH in CH_2Cl_2 as eluent. Fractions containing the product were concentrated in vacuo to obtain the product as a red-brown solid (42.3 mg, 64 µmol, 64%). **^{1}H NMR** (400 MHz, DMSO) δ 8.75 (dd, *J* = 6.7, 2.2 Hz, 2H), 8.15 (d, *J* = 1.6 Hz, 2H), 8.01 (d, *J* = 8.2 Hz, 2H), 7.98 – 7.89 (m, 4H), 7.38 (d, *J* = 5.5 Hz, 2H), 7.17 (dd, *J* = 8.1, 5.8 Hz, 2H). **HRMS:** (TOF-MS ES, m/z) calc. for $C_{22}H_{14}Br_2N_4O_4RuK$ $[M+K]^+$: 698.8037, found: 698.8054. **T_d**: 316 °C. **IR** (cm^{-1}) 3099, 3071, 3050, 2922, 1634, 1586, 1551, 1463, 1418, 1399, 1361, 1291, 1254, 1166, 1146, 1104, 1043, 1026, 904, 837. **UV/Vis:** ε_{380} = 10761 $M^{-1}\cdot cm^{-1}$.

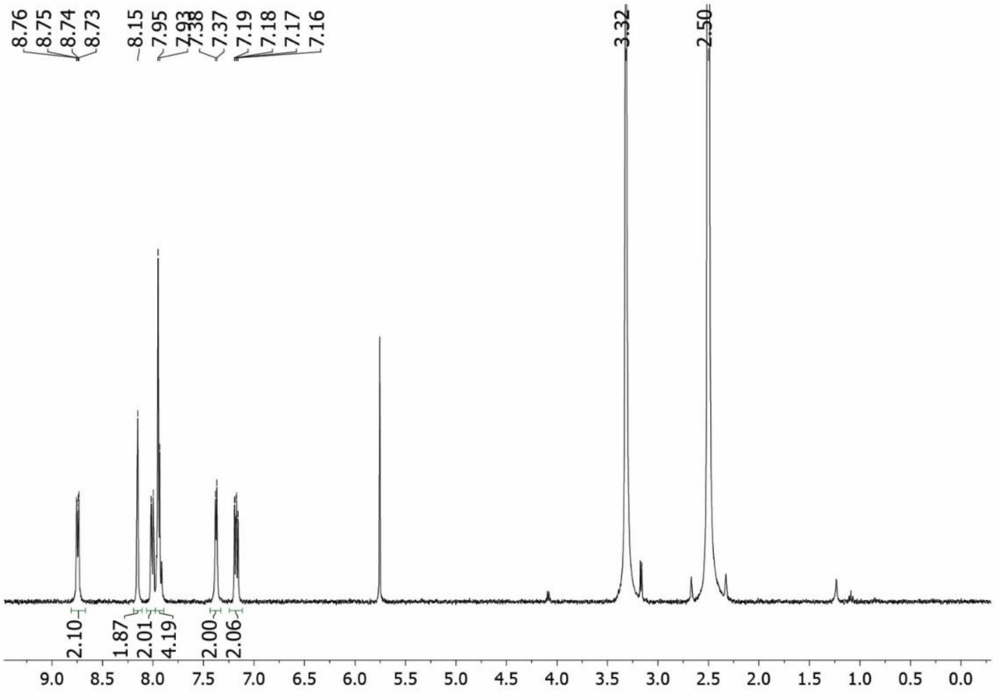

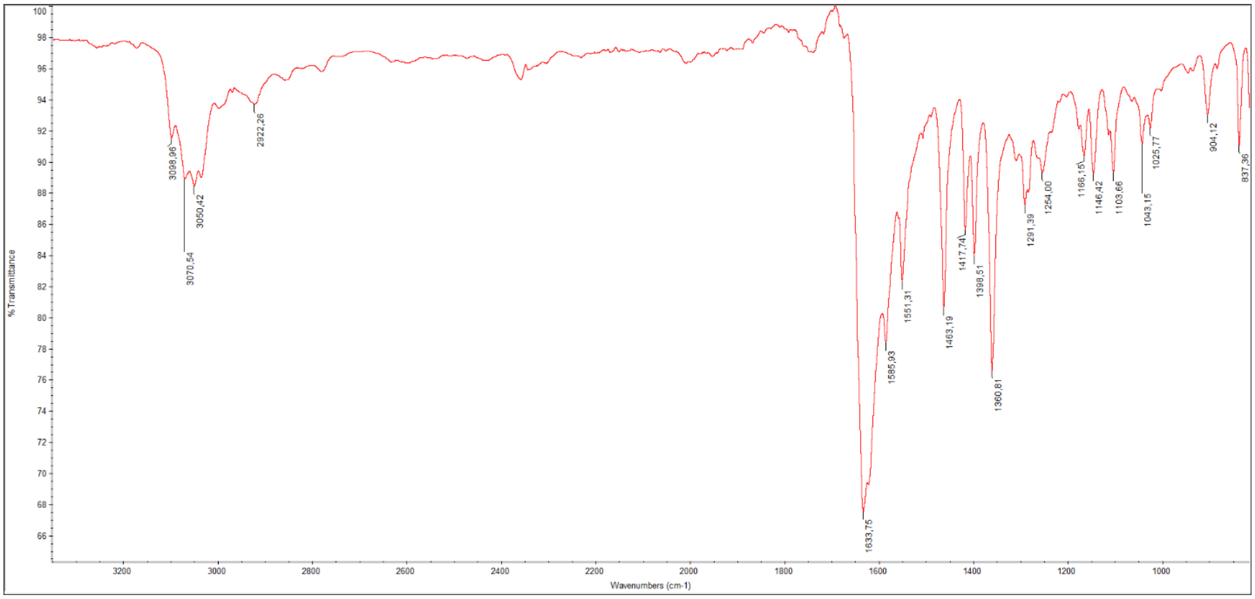

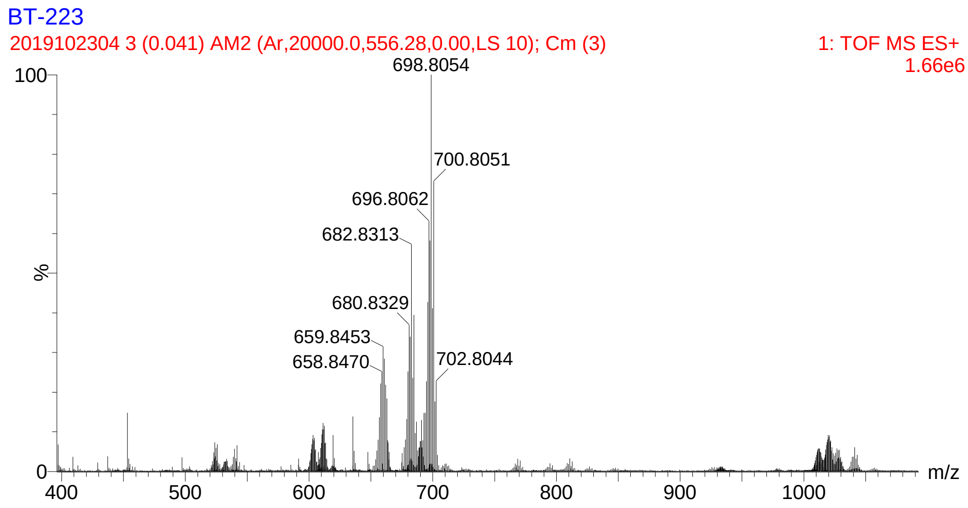
BT-223
2019102304 3 (0.041) AM2 (Ar,20000.0,556.28,0.00,LS 10); Cm (3)
1: TOF MS ES+
1.66e6
698.8054
700.8051
696.8062
682.8313
680.8329
659.8453
658.8470
702.8044
%
m/z

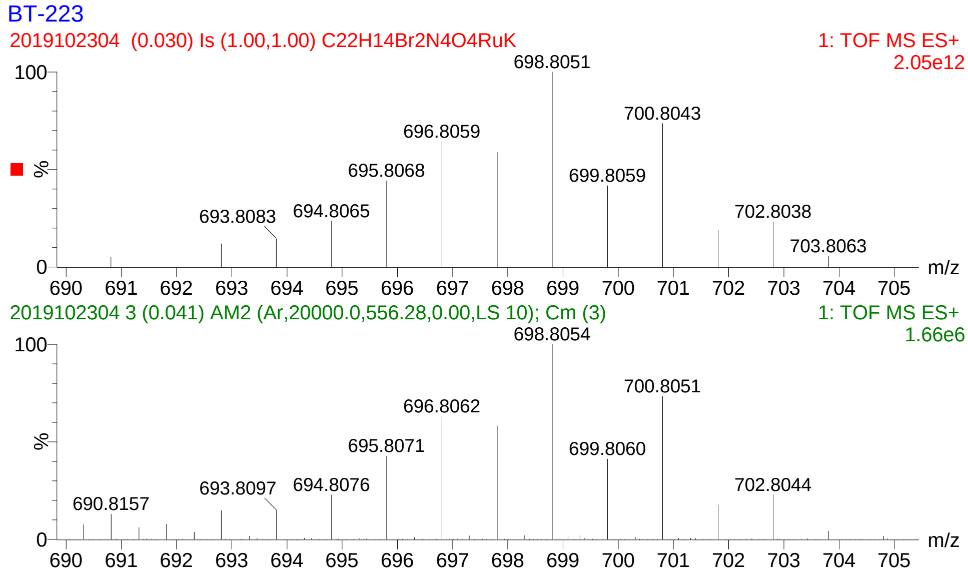
BT-223
2019102304 (0.030) Is (1.00,1.00) C22H14Br2N4O4RuK
1: TOF MS ES+
2.05e12
698.8051
700.8043
696.8059
695.8068
699.8059
693.8083
694.8065
702.8038
703.8063
2019102304 3 (0.041) AM2 (Ar,20000.0,556.28,0.00,LS 10); Cm (3)
1: TOF MS ES+
1.66e6
698.8054
700.8051
696.8062
695.8071
699.8060
690.8157
693.8097
694.8076
702.8044
%
m/z

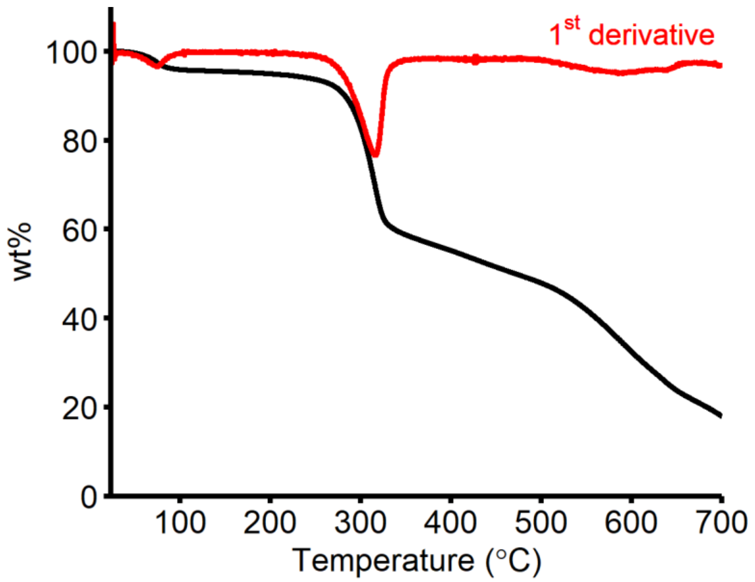
1st derivative
wt%
Temperature (°C)

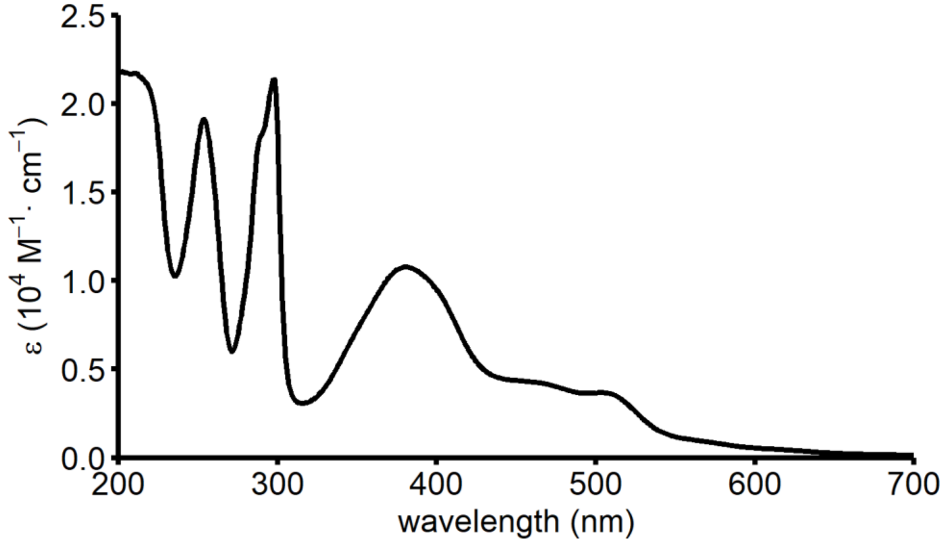
ε (10^4 M^{-1}· cm^{-1})
0.0
0.5
1.0
1.5
2.0
2.5
200
300
400
500
600
700
wavelength (nm)

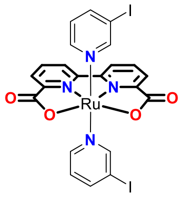

1-*m*I: Pyridine ligand = 3-iodopyridine (2.5 eq., 0.25 mmol, 51 mg). After 17h reaction time, the mixture was concentrated in vacuo and applied on a column of silica gel. The product was eluted using 5% MeOH in CH_2Cl_2 to 10% MeOH in CH_2Cl_2 as eluent. Fractions containing the product were concentrated in vacuo. The product was resuspended in CH_2Cl_2, filtered and washed consecutively with EtOAc and Et_2O to obtain the product as a yellow-brown solid (43.2 mg, 57 µmol, 57%). **^{1}H NMR** (400 MHz, DMSO) δ 8.74 (dd, *J* = 7.1, 1.3 Hz, 2H), 8.29 (s, 2H), 8.09 (d, *J* = 8.0 Hz, 2H), 8.00 – 7.87 (m, 4H), 7.30 (d, *J* = 5.5 Hz, 2H), 7.00 (dd, *J* = 7.9, 5.8 Hz, 2H). **HRMS:** (TOF-MS ES, m/z) calc. for $C_{22}H_{14}I_2N_4O_4RuH$ $[M+H]^+$: 754.8221, found: 754.8233. **T_d**: 328 °C. **IR** (cm^{-1}) 3097, 3055, 3029, 2992, 2974, 1616, 1548, 1461, 1409, 1368, 1320, 1292, 1265, 1254, 1231, 1182, 1148, 1106, 1093, 1050, 1041, 1023, 903, 846, 835. **UV/Vis:** ε_{382} = 11945 $M^{-1}\cdot cm^{-1}$.

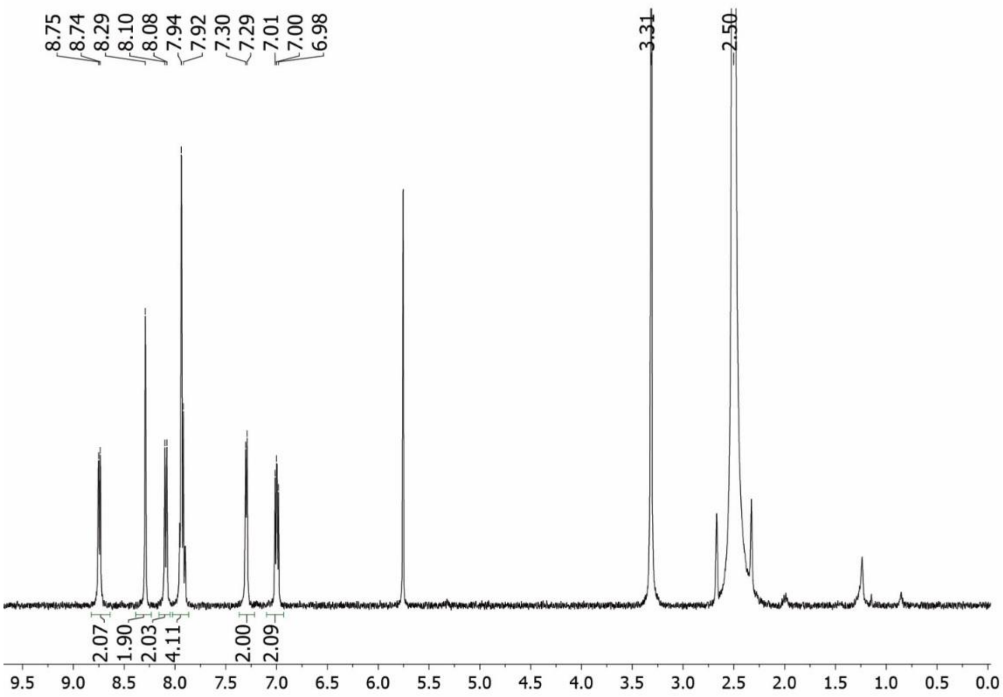

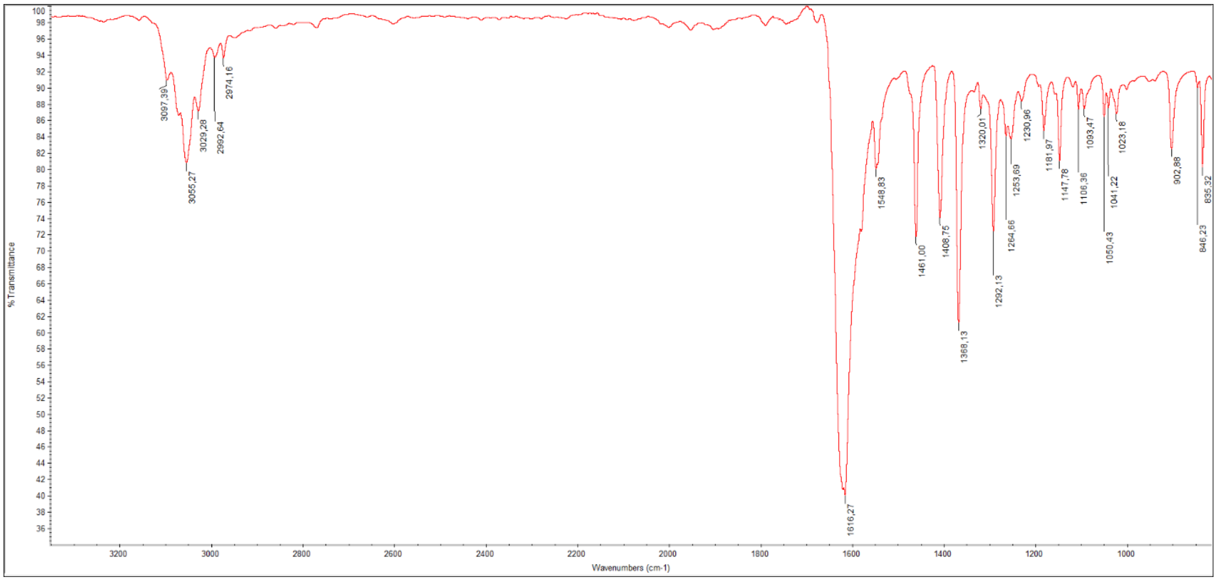

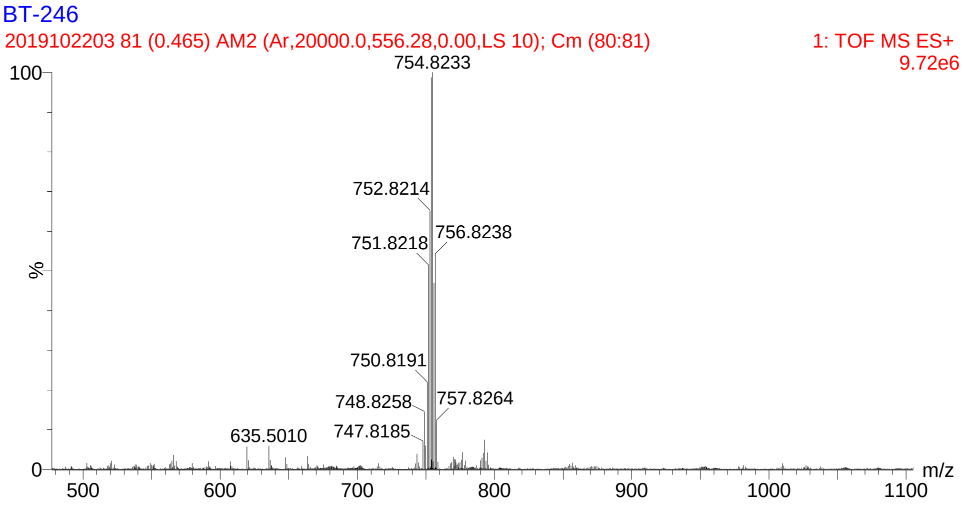
BT-246
2019102203 81 (0.465) AM2 (Ar,20000.0,556.28,0.00,LS 10); Cm (80:81)
1: TOF MS ES+
9.72e6
754.8233
752.8214
751.8218
756.8238
750.8191
748.8258
757.8264
747.8185
635.5010
%
m/z

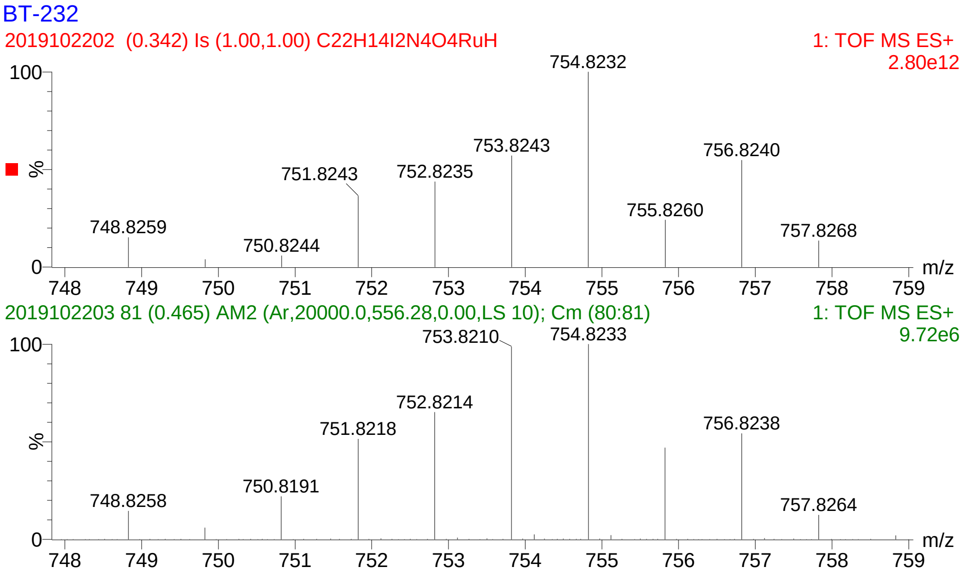
BT-232
2019102202 (0.342) Is (1.00,1.00) C22H14I2N4O4RuH
1: TOF MS ES+
2.80e12
754.8232
753.8243
756.8240
751.8243
752.8235
755.8260
748.8259
757.8268
750.8244
2019102203 81 (0.465) AM2 (Ar,20000.0,556.28,0.00,LS 10); Cm (80:81)
1: TOF MS ES+
9.72e6
753.8210
754.8233
752.8214
751.8218
756.8238
750.8191
748.8258
757.8264
%
m/z

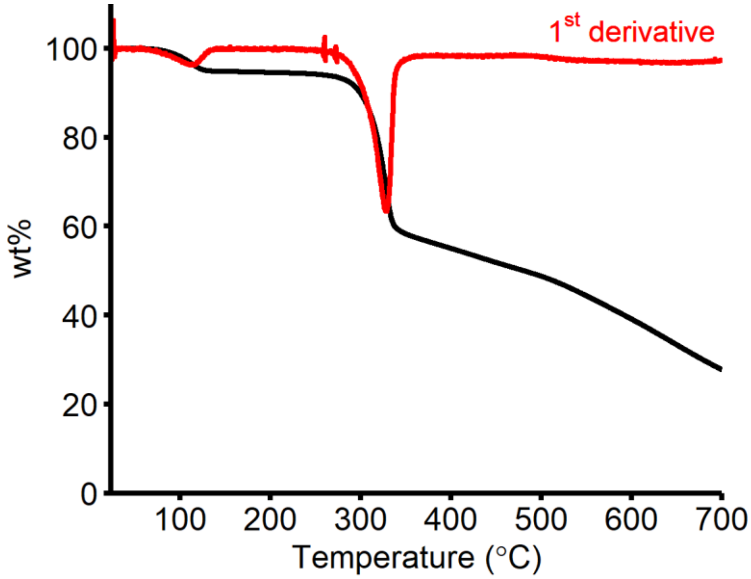
1st derivative
wt%
Temperature (°C)

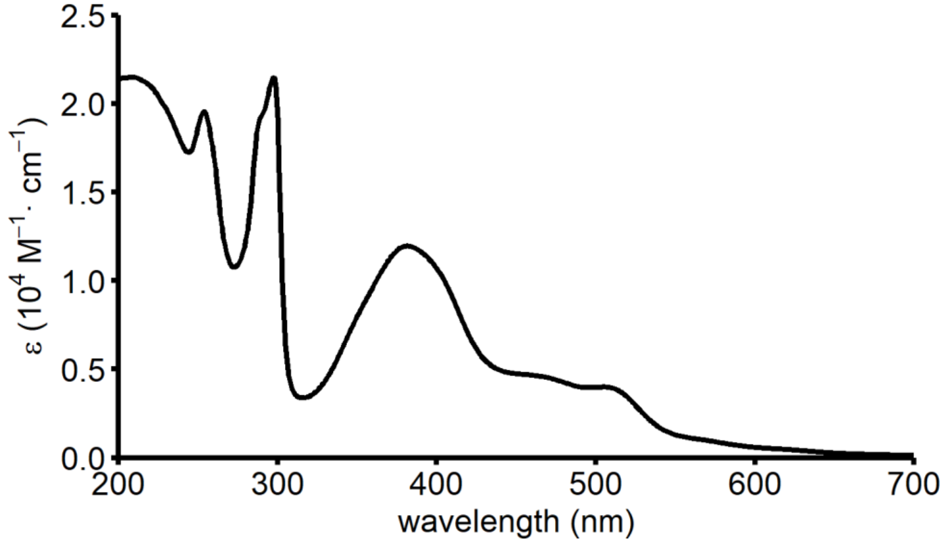
2.5
2.0
1.5
1.0
0.5
0.0
ε (10^4 M^{-1}· cm^{-1})
200
300
400
500
600
700
wavelength (nm)

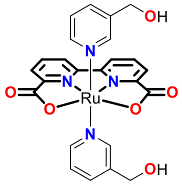

1-*m*CH_2OH: Pyridine ligand = 3-pyridinemethanol (10 eq., 1 mmol, 109 mg, 97 µL). After 18h reaction time, the product was filtered and washed with MeOH and Et_2O to obtain the product as a red-brown solid (26.7 mg, 48 µmol, 48%). **^{1}H NMR** (400 MHz, DMSO) δ 8.71 (d, *J* = 7.3 Hz, 2H), 7.92 – 7.82 (m, 6H), 7.57 (d, *J* = 8.1 Hz, 2H), 7.41 (d, *J* = 5.5 Hz, 2H), 7.17 (dd, *J* = 7.6, 5.9 Hz, 2H), 5.37 (t, *J* = 5.6 Hz, 2H), 4.35 (d, *J* = 5.6 Hz, 4H). **HRMS:** (TOF-MS ES, m/z) calc. for $C_{24}H_{19}N_4O_6Ru$ $[M-H]^-$: 561.0354, found: 561.0358. **T_d**: 314 °C. **IR** (cm^{-1}) 3271, 3050, 3026, 2906, 2855, 1623, 1601, 1473, 1433, 1407, 1366, 1311, 1298, 1257, 1227, 1201, 1180, 1151, 1107, 1055, 1024, 998, 940, 908, 833, 798, 777, 712, 700. **UV/Vis:** ε_{367} = 10430 $M^{-1}\cdot cm^{-1}$.

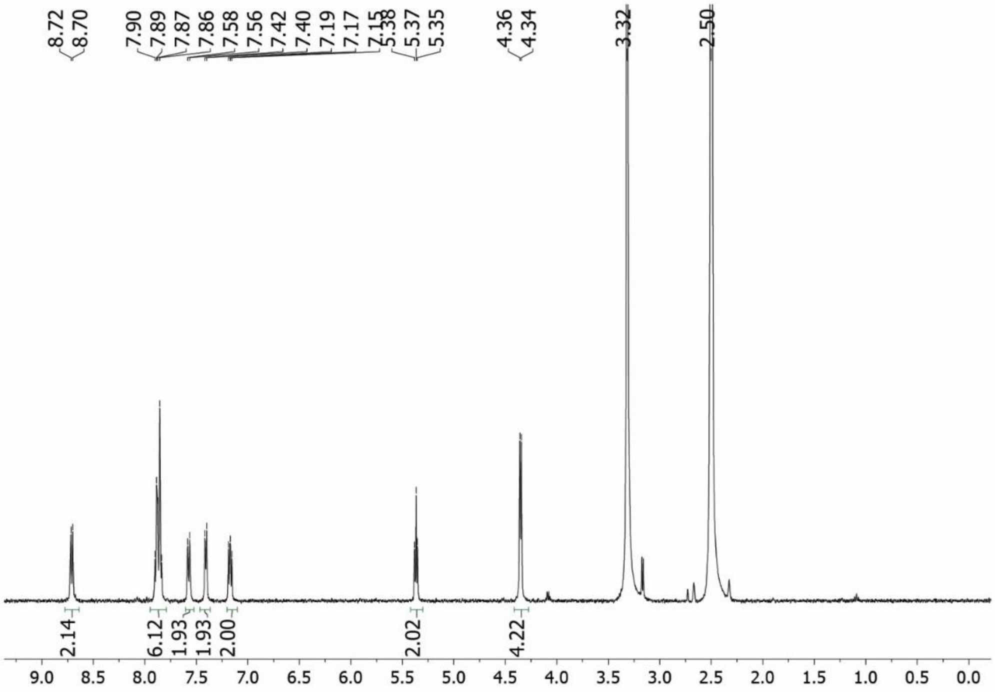

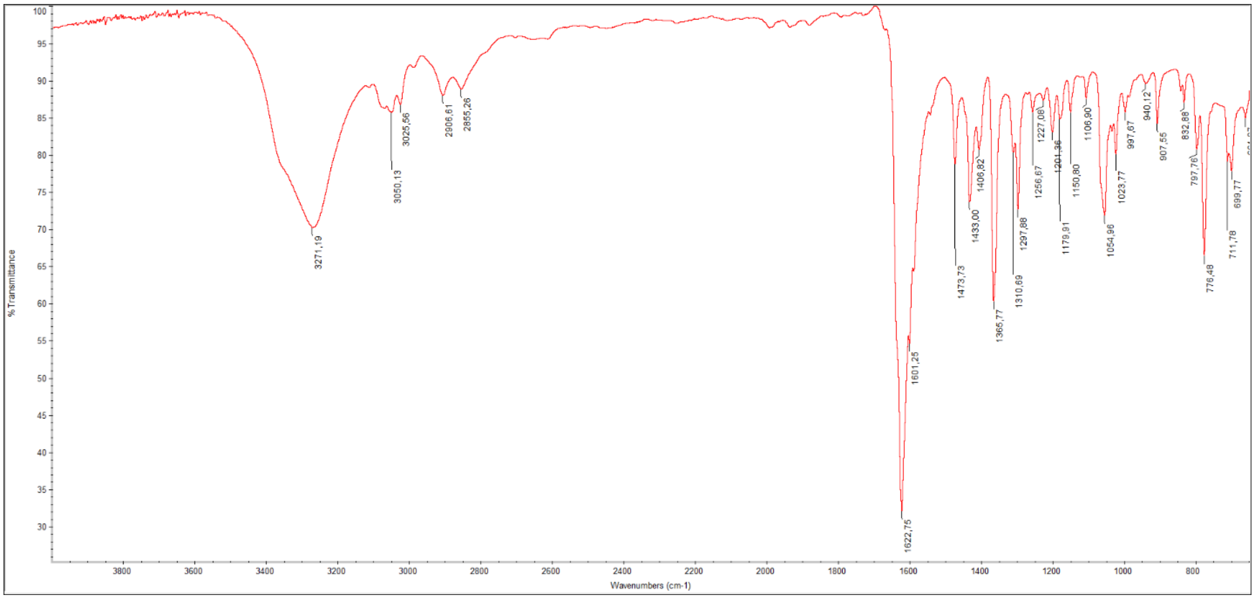

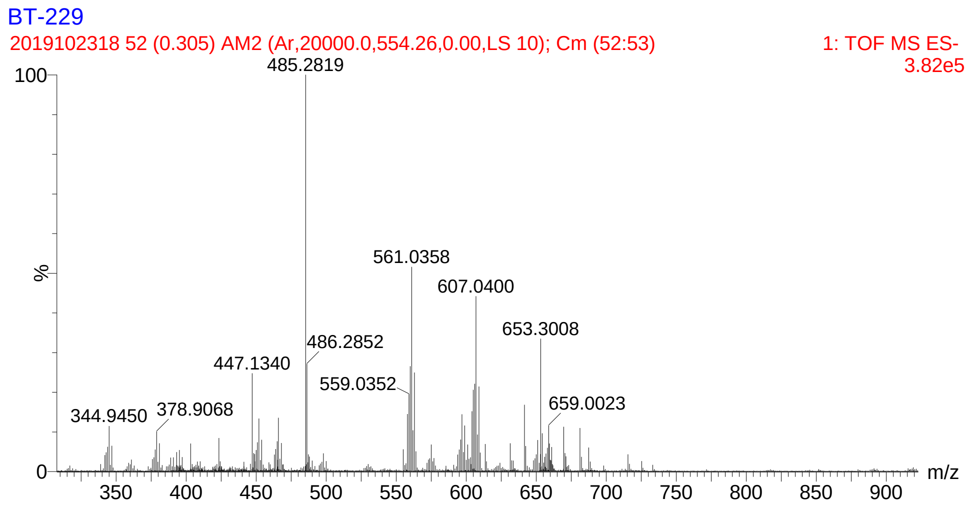
BT-229
2019102318 52 (0.305) AM2 (Ar,20000.0,554.26,0.00,LS 10); Cm (52:53)
1: TOF MS ES-
3.82e5
485.2819
561.0358
607.0400
653.3008
486.2852
447.1340
559.0352
659.0023
344.9450
378.9068
%
m/z

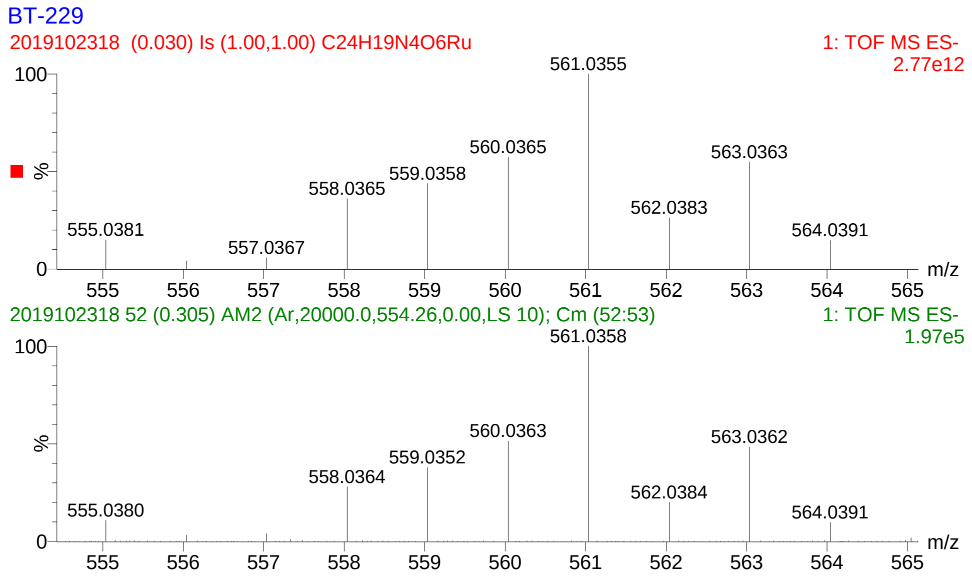
BT-229
2019102318 (0.030) Is (1.00,1.00) C24H19N4O6Ru
1: TOF MS ES-
2.77e12
561.0355
560.0365
563.0363
559.0358
558.0365
562.0383
555.0381
564.0391
557.0367
2019102318 52 (0.305) AM2 (Ar,20000.0,554.26,0.00,LS 10); Cm (52:53)
1: TOF MS ES-
1.97e5
561.0358
560.0363
563.0362
559.0352
558.0364
562.0384
555.0380
564.0391
%
m/z

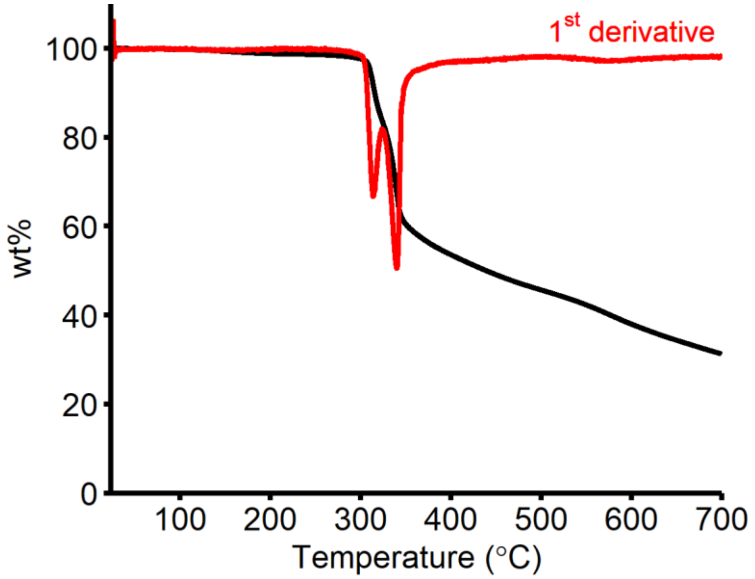
1st derivative
wt%
Temperature (°C)

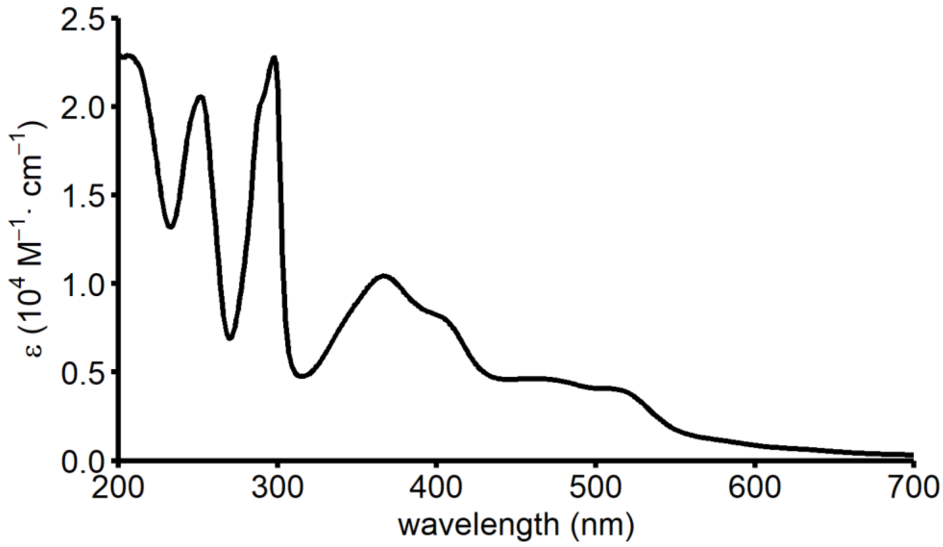

2.5
2.0
1.5
1.0
0.5
0.0
ε (10^4 M^−1 · cm^−1)
200
300
400
500
600
700
wavelength (nm)

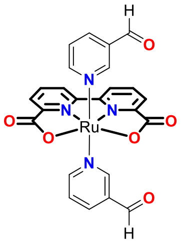

1-*m*CHO: Pyridine ligand = 3-pyridinecarbaldehyde (2.5 eq., 0.25 mmol, 27 mg, 24 µL). After 21h reaction time, a few drops of H_2O were added and the reaction mixture was stirred for an additional 10 min. The product was then filtered and washed with MeOH, EtOAc and Et_2O to obtain the product as a red-brown solid (29.0 mg, 52 µmol, 52%). NOTE: The product is obtained as a mixture of the hydrate/(hemi)acetal, which cleanly converts to the aldehyde upon standing in d_6-DMSO overnight. **^{1}H NMR** (400 MHz, DMSO) δ 9.88 (s, 2H), 8.79 (dd, J = 6.6, 2.3 Hz, 2H), 8.33 (s, 2H), 8.15 (d, J = 7.7 Hz, 2H), 7.97 – 7.89 (m, 4H), 7.85 (d, J = 5.6 Hz, 2H), 7.47 (dd, J = 7.5, 6.0 Hz, 2H). **HRMS:** (TOF-MS ES, m/z) calc. for $C_{24}H_{15}N_4O_6Ru$ $[M-H]^-$: 557.0041, found: 557.0045. **T*d*:** 233 °C. **IR** (cm^{-1}) 3232, 3074, 2992, 2933, 2828, 1610, 1586, 1478, 1425, 1401, 1364, 1321, 1301, 1211, 1181, 1090, 1065, 1050, 971, 787, 715, 707. **UV/Vis:** ε_{396} = 9277 M^{-1}·cm^{-1}.

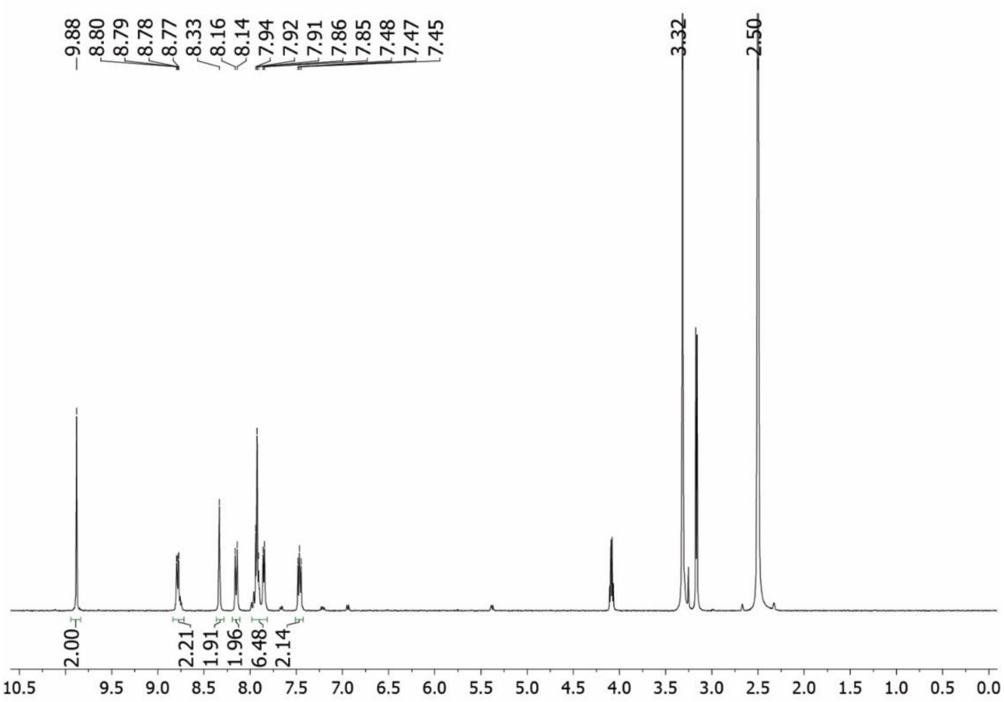

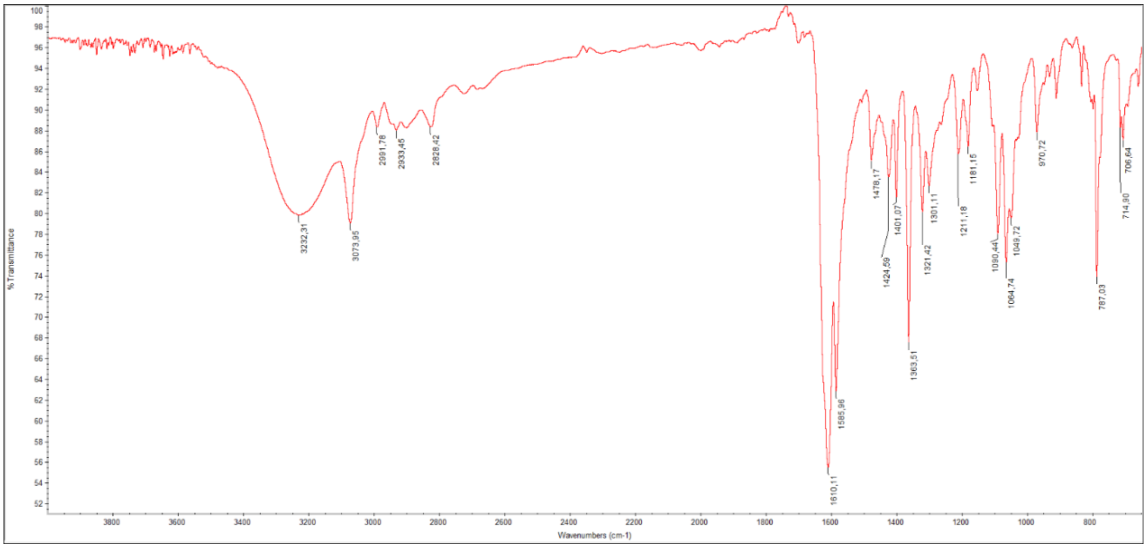

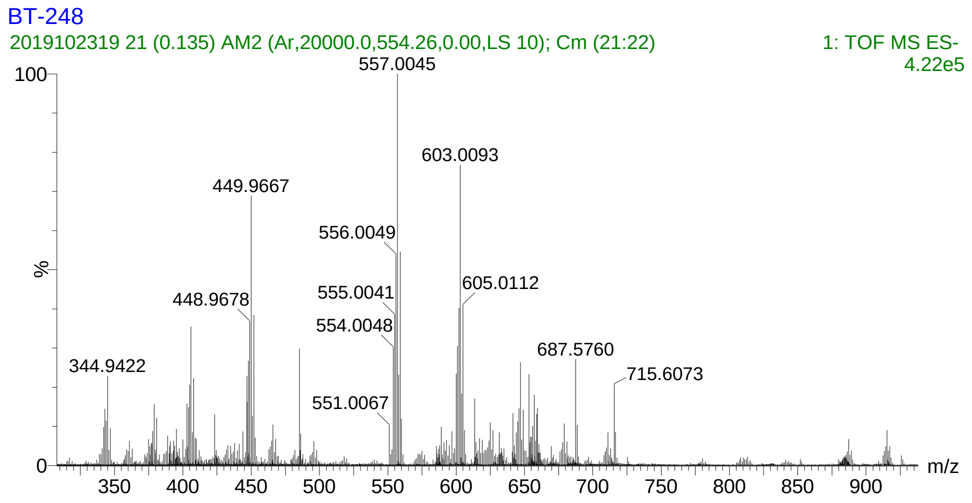
BT-248
2019102319 21 (0.135) AM2 (Ar,20000.0,554.26,0.00,LS 10); Cm (21:22)
1: TOF MS ES-
4.22e5
557.0045
603.0093
449.9667
556.0049
555.0041
605.0112
448.9678
554.0048
687.5760
344.9422
715.6073
551.0067
100
%
0
350
400
450
500
550
600
650
700
750
800
850
900
m/z

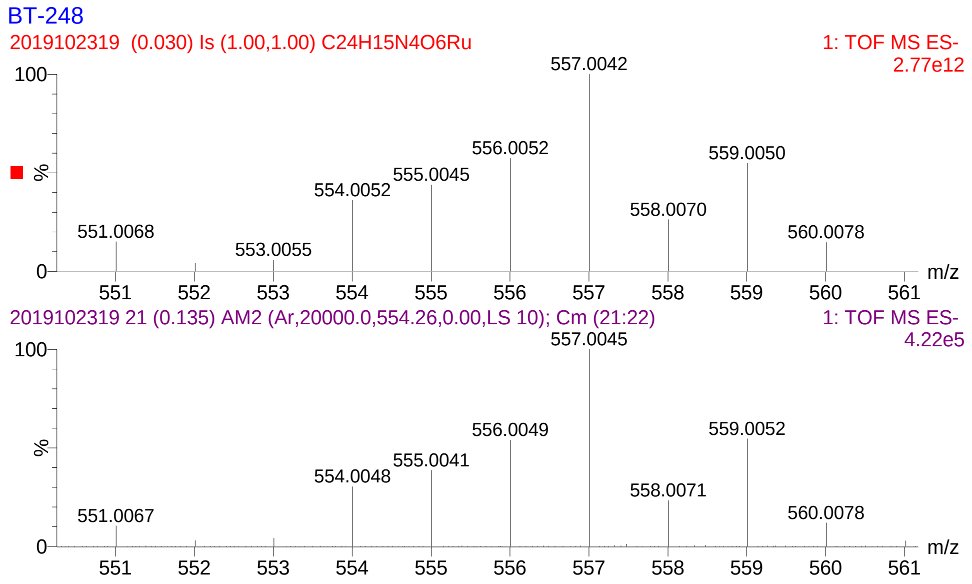
BT-248
2019102319 (0.030) Is (1.00,1.00) C24H15N4O6Ru
1: TOF MS ES-
2.77e12
557.0042
556.0052
559.0050
555.0045
554.0052
558.0070
551.0068
560.0078
553.0055
2019102319 21 (0.135) AM2 (Ar,20000.0,554.26,0.00,LS 10); Cm (21:22)
1: TOF MS ES-
4.22e5
557.0045
556.0049
559.0052
555.0041
554.0048
558.0071
551.0067
560.0078
100
%
0
551
552
553
554
555
556
557
558
559
560
561
m/z

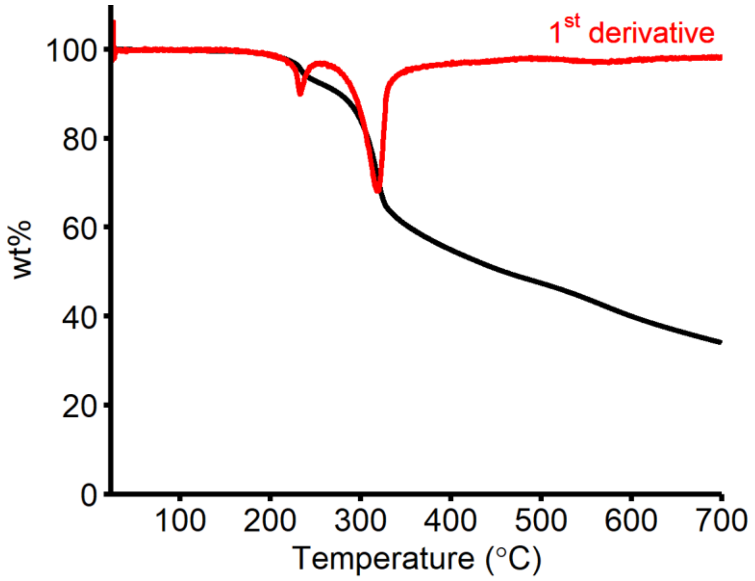
1st derivative
wt%
100
80
60
40
20
0
100
200
300
400
500
600
700
Temperature (°C)

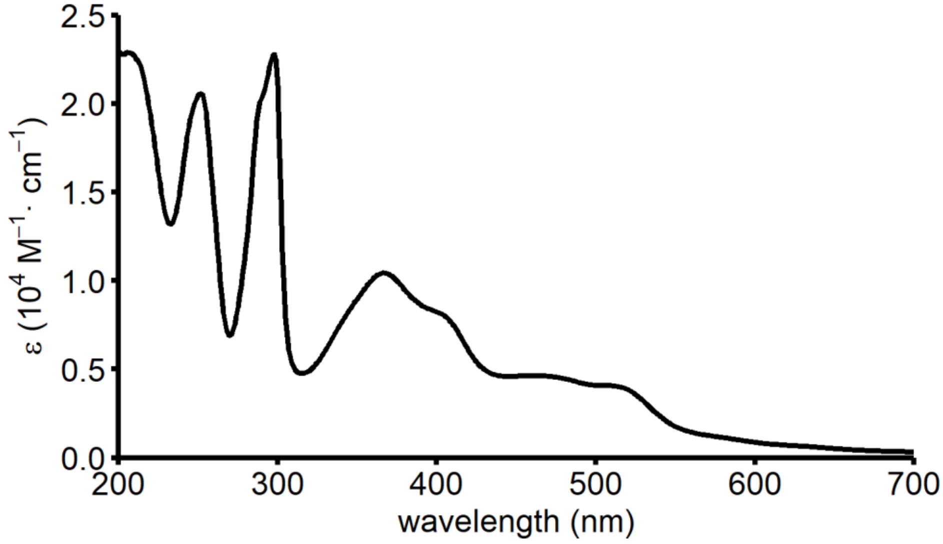
2.5
2.0
1.5
1.0
0.5
0.0
ε (10⁴ M⁻¹· cm⁻¹)
200
300
400
500
600
700
wavelength (nm)

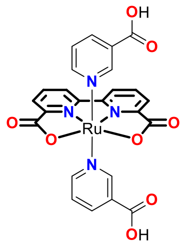

1-*m*CO_2H: Pyridine ligand = 3-pyridinecarboxylic acid (10 eq., 1.0 mmol, 123 mg). After 16.5h reaction time the product was filtered off and washed with MeOH and Et_2O to obtain the product as a red-brown solid (32.6 mg, 55 µmol, 55%). **^{1}H NMR** (400 MHz, DMSO) δ 13.77 (s, 2H), 8.78 (dd, *J* = 5.8, 2.9 Hz, 2H), 8.41 (s, 2H), 8.13 (d, *J* = 7.7 Hz, 2H), 8.00-7.90 (m, 4H), 7.75 (d, *J* = 5.3 Hz, 2H), 7.44 – 7.25 (m, 2H). **HRMS:** (TOF-MS ES, m/z) calc. for $C_{24}H_{16}N_4O_8RuH$ $[M+H]^+$: 591.0096, found: 591.0094. **T_d**: 338 °C. **IR** (cm^{-1}) 3071, 2832, 2432, 1706, 1607, 1594, 1568, 1471, 1430, 1402, 1360, 1321, 1311, 1297, 1224, 1182, 1137, 1115, 1024, 836, 771, 749, 714, 704, 963, 677. **UV/Vis:** ε_{391} = 9579 $M^{-1}\cdot cm^{-1}$.

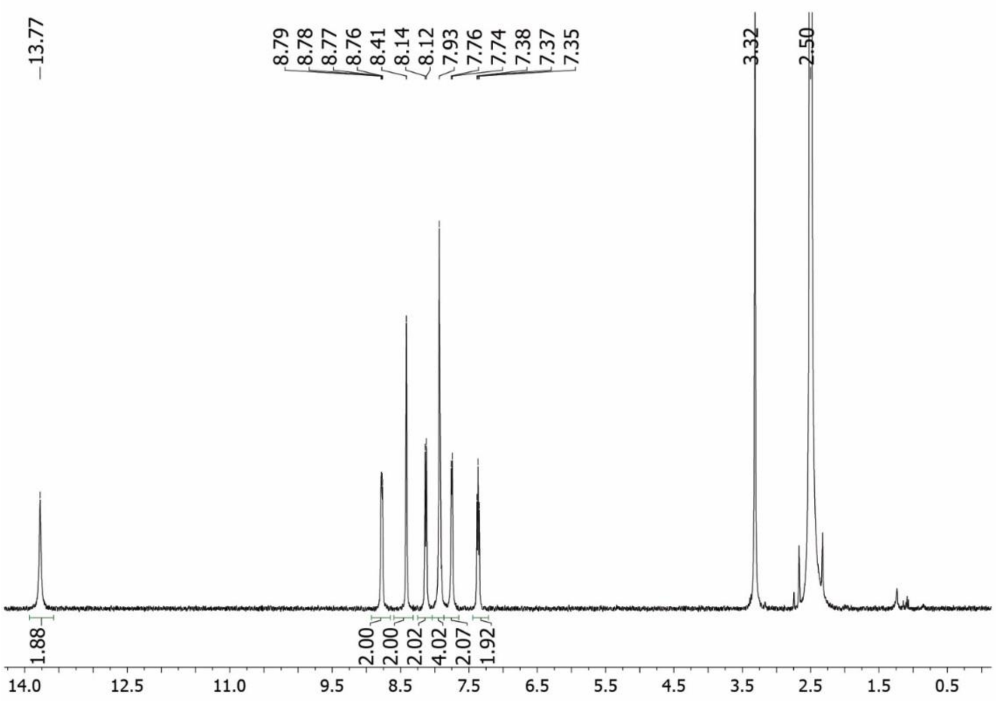

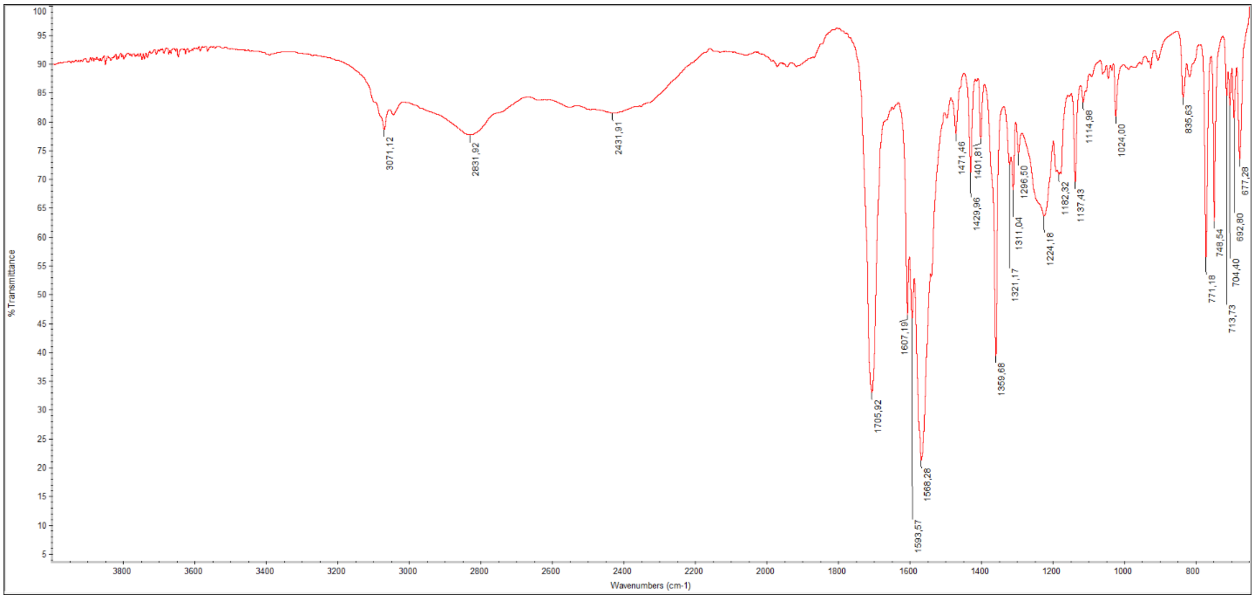

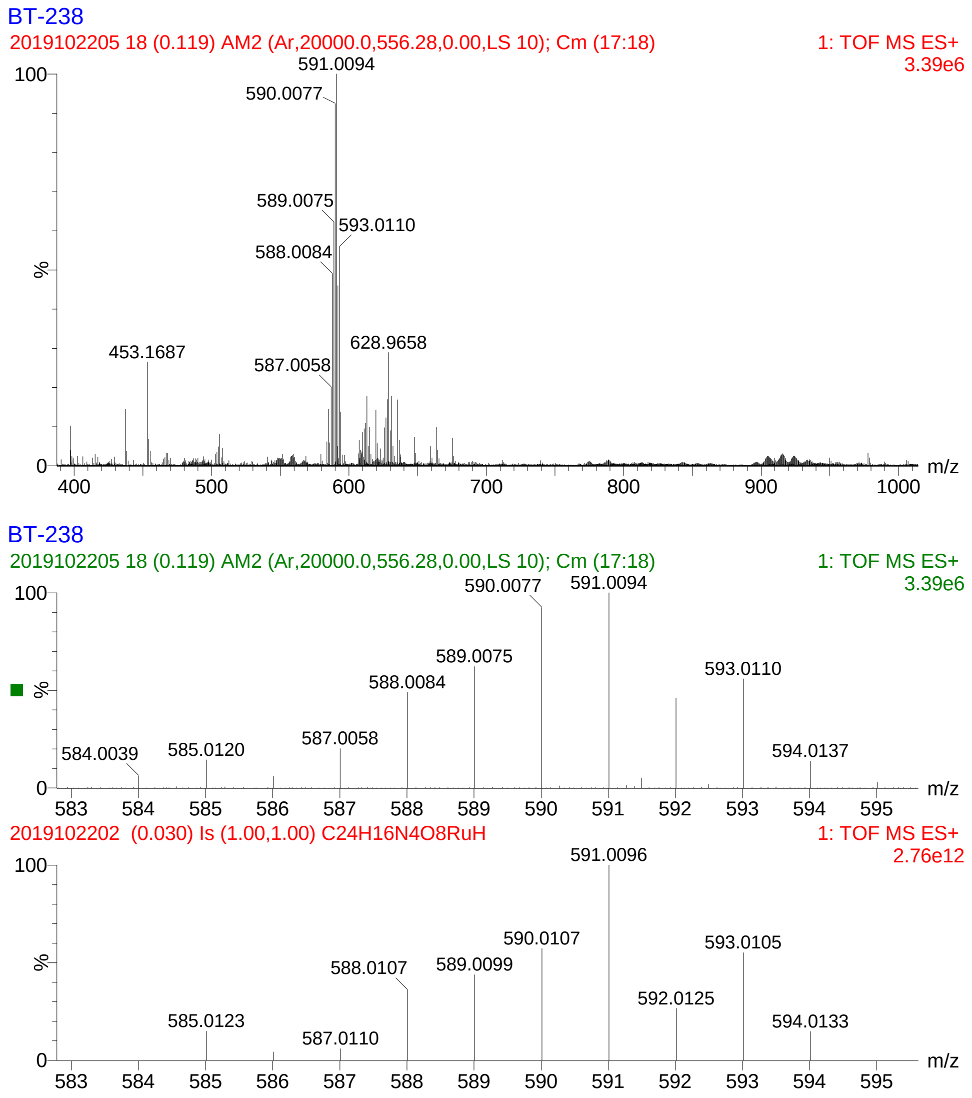
BT-238
2019102205 18 (0.119) AM2 (Ar,20000.0,556.28,0.00,LS 10); Cm (17:18)
1: TOF MS ES+
3.39e6
591.0094
590.0077
589.0075
593.0110
588.0084
628.9658
453.1687
587.0058
%
m/z
BT-238
2019102205 18 (0.119) AM2 (Ar,20000.0,556.28,0.00,LS 10); Cm (17:18)
1: TOF MS ES+
3.39e6
590.0077
591.0094
589.0075
593.0110
588.0084
587.0058
584.0039
585.0120
594.0137
2019102202 (0.030) Is (1.00,1.00) C24H16N4O8RuH
1: TOF MS ES+
2.76e12
591.0096
590.0107
593.0105
588.0107
589.0099
592.0125
585.0123
594.0133
587.0110
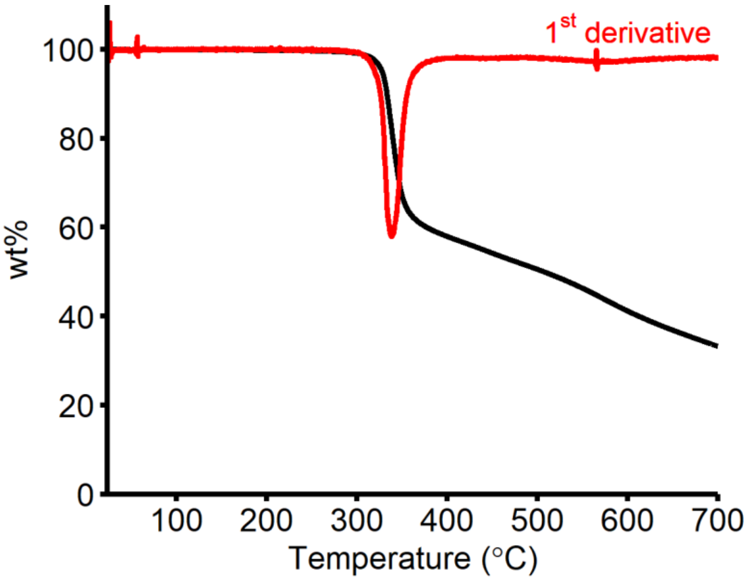
1st derivative
wt%
Temperature (°C)

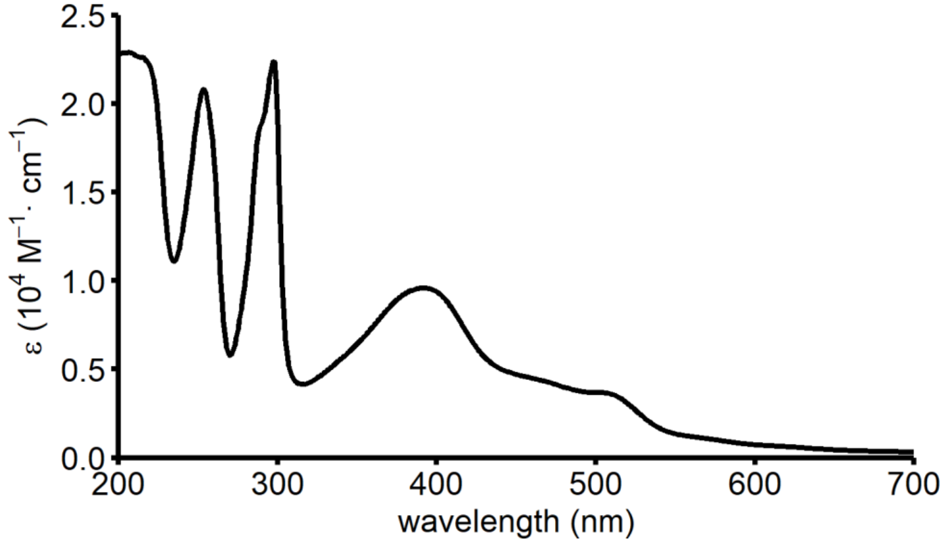

2.5
2.0
1.5
1.0
0.5
0.0
ε (10⁴ M⁻¹· cm⁻¹)
200
300
400
500
600
700
wavelength (nm)

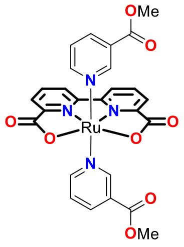

1-*m*CO₂Me: Pyridine ligand = methyl nicotinate (10 eq., 1 mmol, 137 mg). After 16.5h reaction time, the mixture was concentrated in vacuo and applied on a column of silica gel. The product was eluted using 5% MeOH in CH_2Cl_2 to 10% MeOH in CH_2Cl_2 as eluent. Fractions containing the product were concentrated in vacuo. The product was resuspended in EtOAc, filtered and washed with Et_2O to obtain the product as a red-brown solid (37.7 mg, 61 µmol, 61%). **^{1}H NMR** (400 MHz, DMSO) δ 8.79 (dd, *J* = 5.8, 3.2 Hz, 2H), 8.51 (s, 2H), 8.16 (d, *J* = 7.9 Hz, 2H), 8.00 – 7.87 (m, 4H), 7.70 (d, *J* = 5.4 Hz, 2H), 7.37 (dd, *J* = 7.7, 6.0 Hz, 2H), 3.84 (s, 6H). **HRMS:** (TOF-MS ES, m/z) calc. for $C_{26}H_{20}N_4O_8RuH$ $[M+H]^+$: 619.0410, found: 619.0415. **T_d**: 270 °C. **IR** (cm^{-1}) 3545, 3460, 3056, 1741, 1729, 1722, 1627, 1474, 1435, 1426, 1411, 1370, 1318, 1292, 1251, 1183, 1130, 1115, 1046, 1025, 960, 906, 835, 784, 750, 708, 691. **UV/Vis:** ε_{393} = 10597 $M^{-1}\cdot cm^{-1}$.

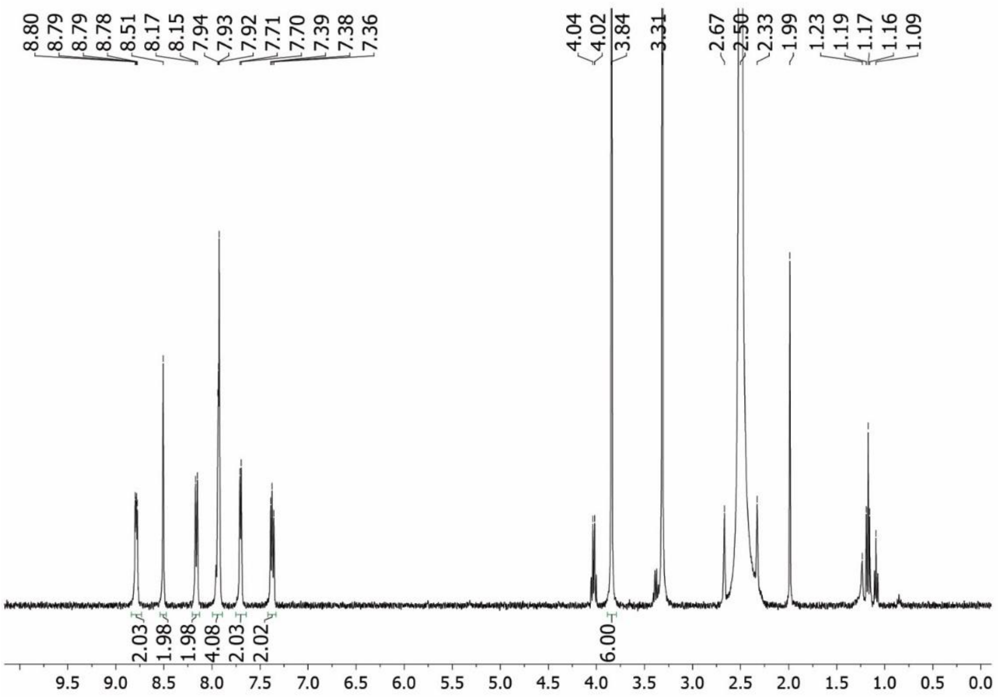

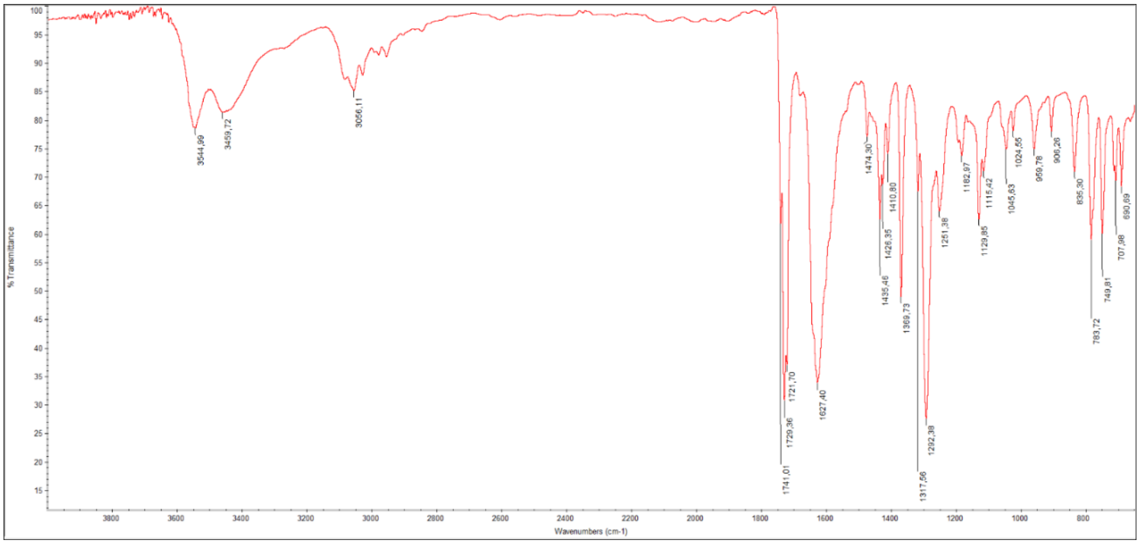

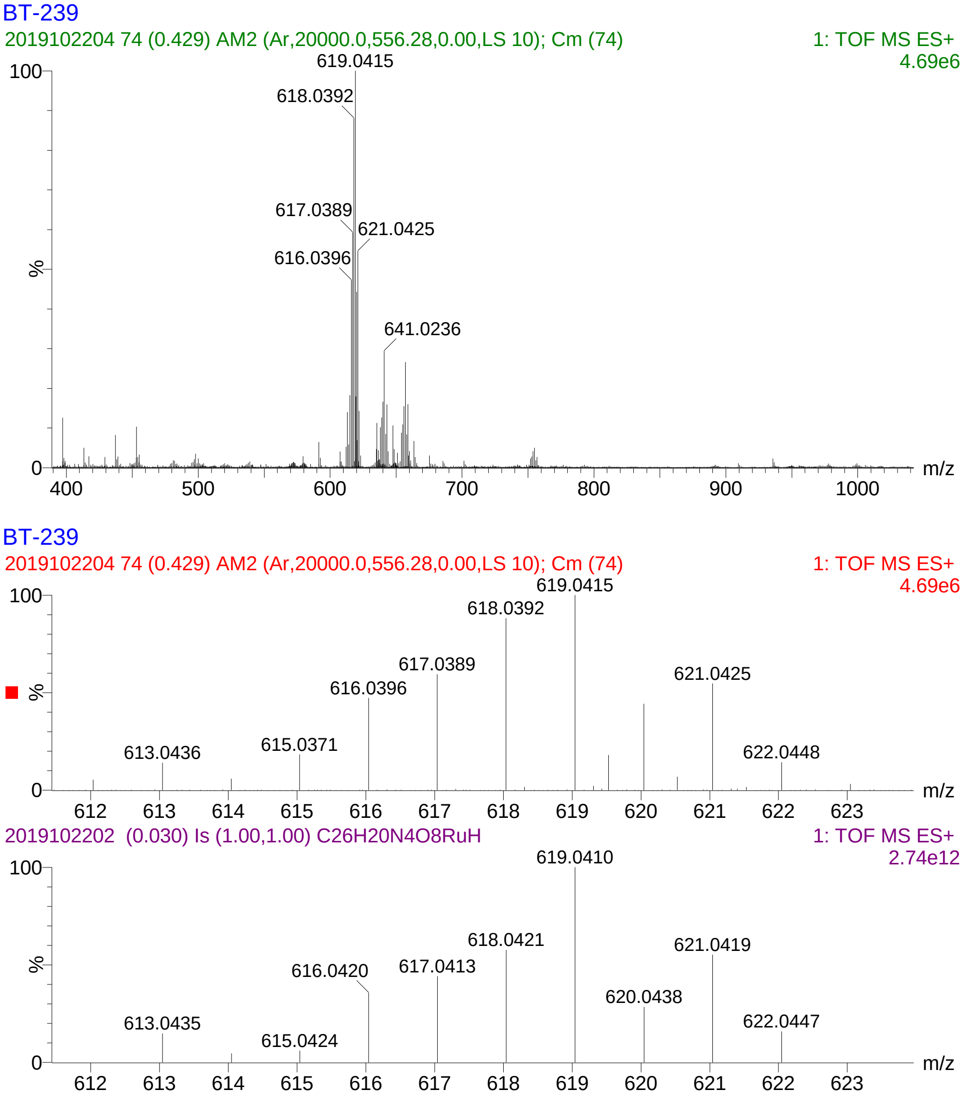
BT-239
2019102204 74 (0.429) AM2 (Ar,20000.0,556.28,0.00,LS 10); Cm (74)
1: TOF MS ES+
4.69e6
619.0415
618.0392
617.0389
621.0425
616.0396
641.0236
%
m/z
BT-239
2019102204 74 (0.429) AM2 (Ar,20000.0,556.28,0.00,LS 10); Cm (74)
1: TOF MS ES+
4.69e6
619.0415
618.0392
617.0389
616.0396
621.0425
613.0436
615.0371
622.0448
2019102202 (0.030) Is (1.00,1.00) C26H20N4O8RuH
1: TOF MS ES+
2.74e12
619.0410
618.0421
621.0419
616.0420
617.0413
620.0438
613.0435
622.0447
615.0424

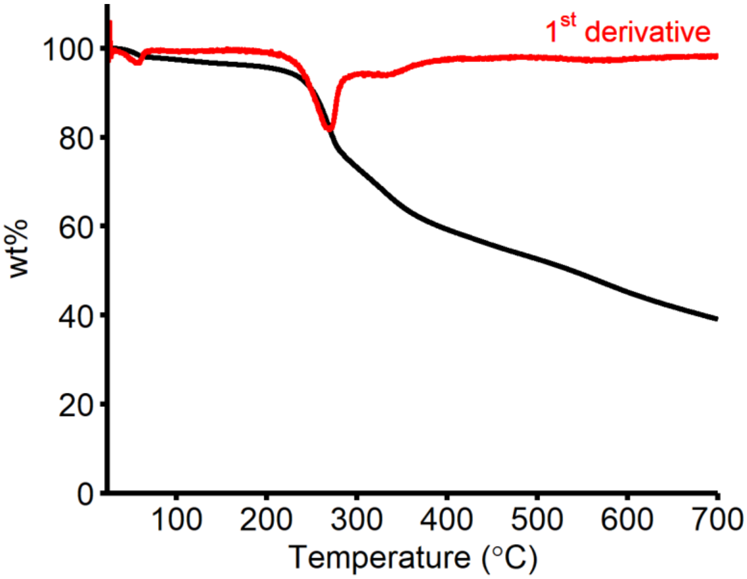
1st derivative
wt%
Temperature (°C)

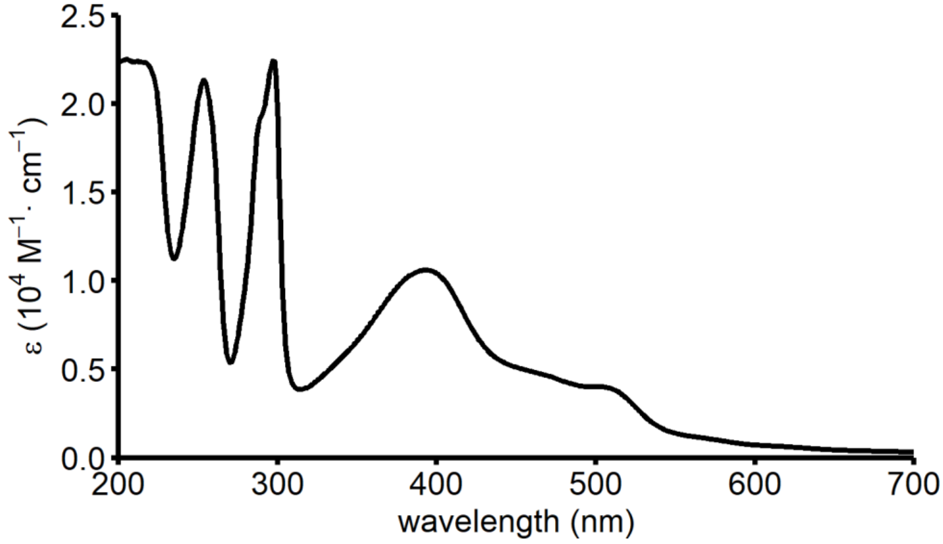
2.5
2.0
1.5
1.0
0.5
0.0
ε (10^4 M^-1 · cm^-1)
200
300
400
500
600
700
wavelength (nm)

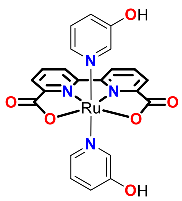

1-*m*OH: Pyridine ligand = 3-hydroxypyridine (10 eq., 1 mmol, 95 mg). After 21h reaction time, the product was filtered and washed with MeOH and Et_2O to obtain the product as a brown solid (23.5 mg, 37 µmol, 37%). NOTE: The product was obtained with three 3-hydroxypyridine ligands attached to the ruthenium center. Further attempts to isolate a product with two ligands were unsuccessful. **^{1}H NMR** (400 MHz, DMSO) δ 10.40 (s, 2H), 9.85 (s, 1H), 8.68 (d, *J* = 7.8 Hz, 2H), 8.12 (d, *J* = 2.6 Hz, 1H), 8.02 (d, *J* = 3.5 Hz, 1H), 7.92 (d, *J* = 7.4 Hz, 2H), 7.85 (t, *J* = 7.8 Hz, 2H), 7.38 (s, 2H), 7.22 – 7.12 (m, 2H), 7.06 – 6.98 (m, 6H). **HRMS:** (TOF-MS ES, m/z) calc. for $C_{22}H_{15}N_4O_6Ru$ $[M-L-H]^+$: 533.0035, found: 533.0040. **T_d**: 250 °C. **IR** (cm^{-1}) 3068, 2918, 2824, 2762, 2703, 2635, 2576, 2467, 1623, 1600, 1569, 1478, 1043, 1362, 1311, 1283, 1234, 1203, 1184, 1158, 1104, 1029. **UV/Vis:** ε_{350} = 7505 $M^{-1}\cdot cm^{-1}$.

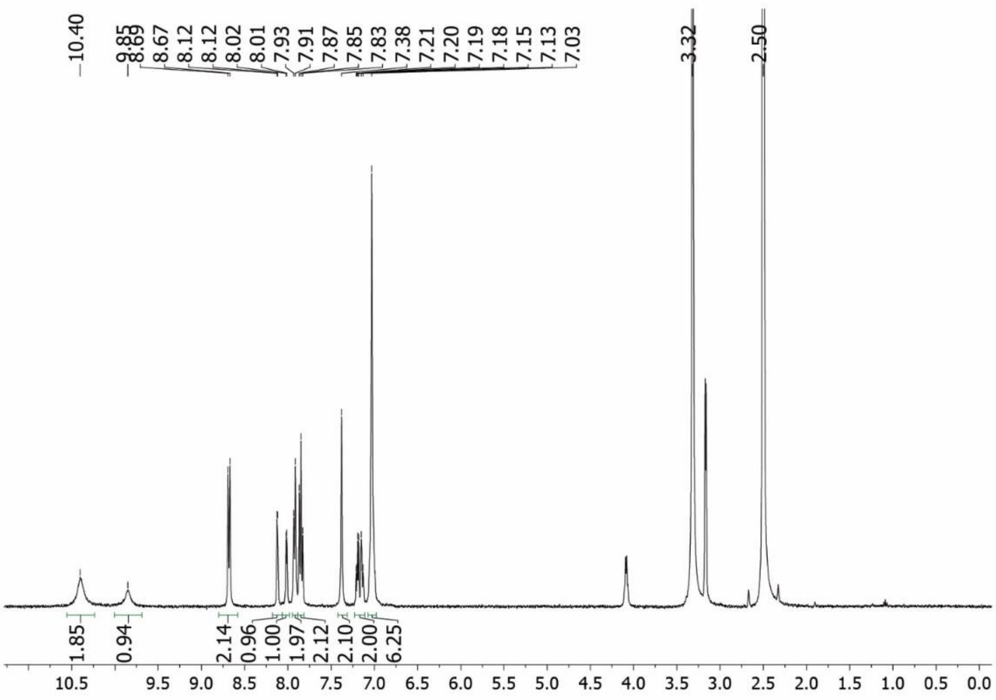

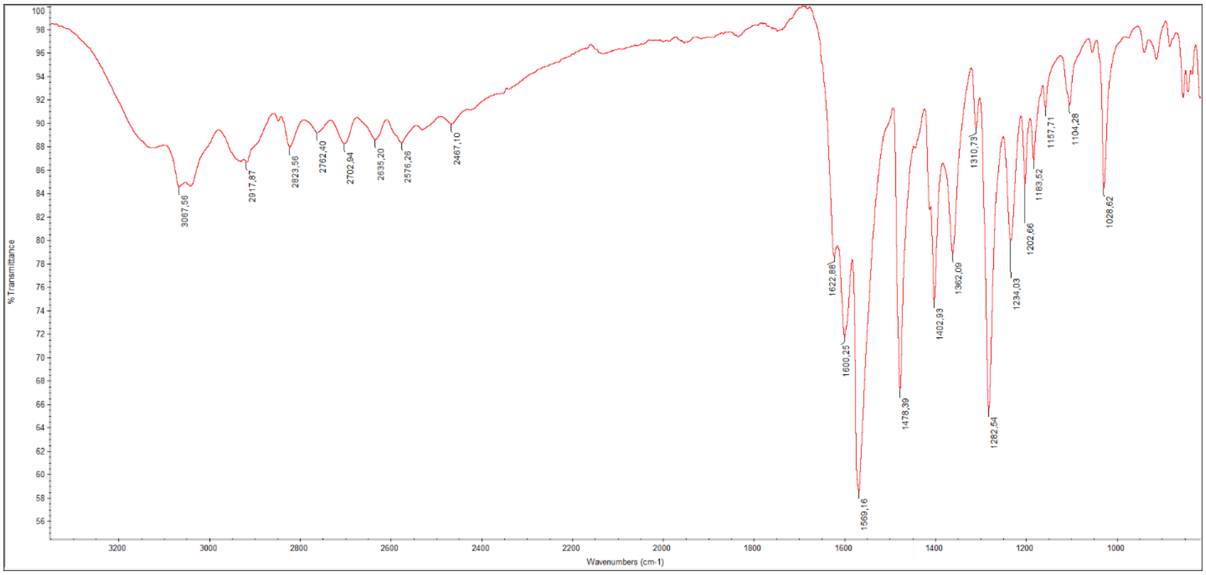

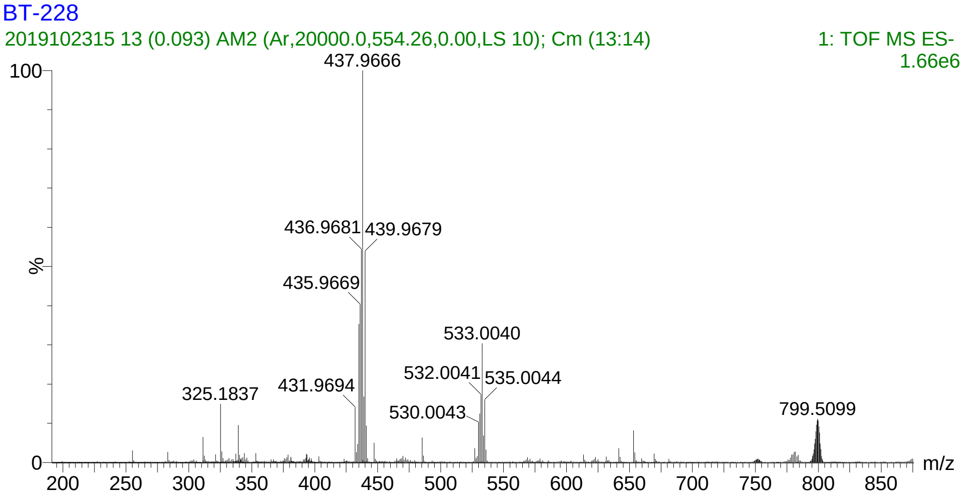
BT-228
2019102315 13 (0.093) AM2 (Ar,20000.0,554.26,0.00,LS 10); Cm (13:14)
1: TOF MS ES-
1.66e6
437.9666
436.9681
439.9679
435.9669
533.0040
532.0041
535.0044
325.1837
431.9694
530.0043
799.5099
100
%
0
200
250
300
350
400
450
500
550
600
650
700
750
800
850
m/z

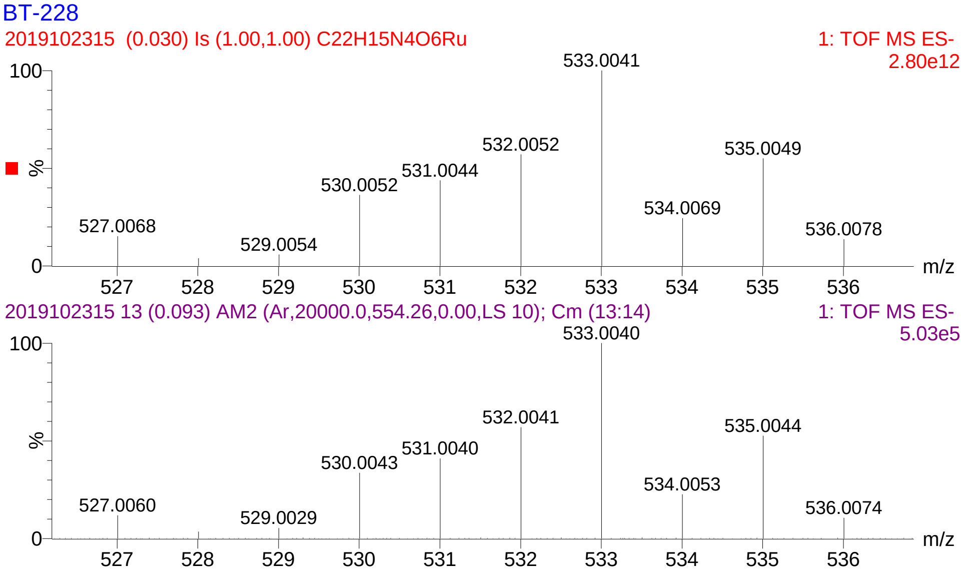
BT-228
2019102315 (0.030) Is (1.00,1.00) C22H15N4O6Ru
1: TOF MS ES-
2.80e12
533.0041
532.0052
535.0049
531.0044
530.0052
534.0069
527.0068
536.0078
529.0054
2019102315 13 (0.093) AM2 (Ar,20000.0,554.26,0.00,LS 10); Cm (13:14)
1: TOF MS ES-
5.03e5
533.0040
532.0041
535.0044
531.0040
530.0043
534.0053
527.0060
536.0074
529.0029
100
%
0
527
528
529
530
531
532
533
534
535
536
m/z

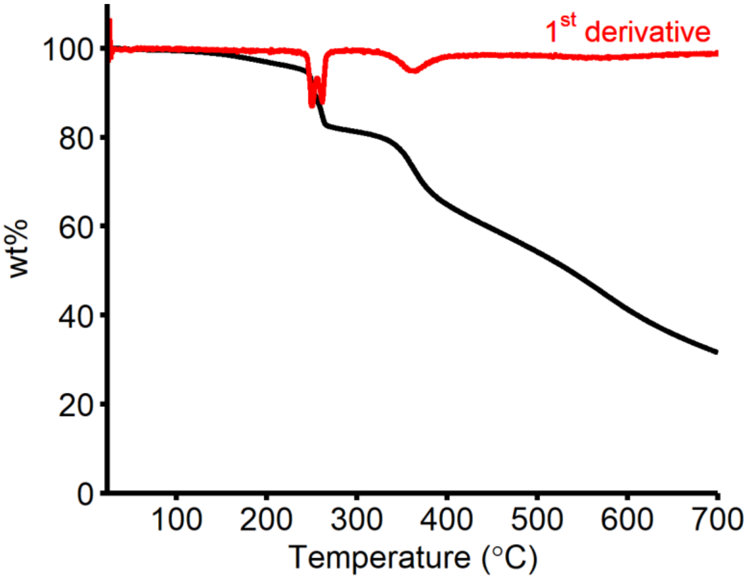
1st derivative
wt%
100
80
60
40
20
0
100
200
300
400
500
600
700
Temperature (°C)

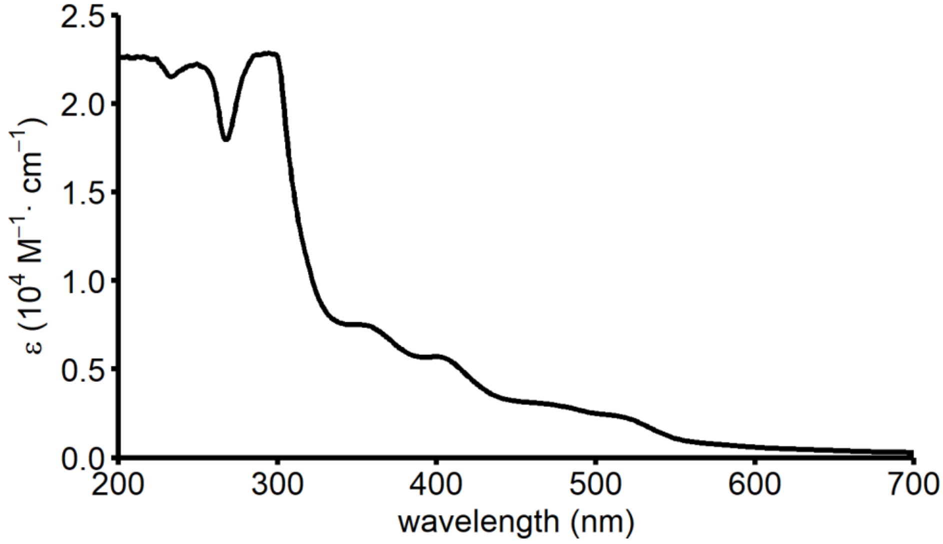
2.5
2.0
1.5
1.0
0.5
0.0
ε (10^4 M^{-1}· cm^{-1})
200
300
400
500
600
700
wavelength (nm)

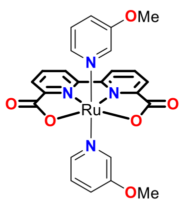

1-*m*OMe: Pyridine ligand = 3-methoxypyridine (10 eq., 1 mmol, 155 mg, 142 µL). After 17.5h reaction time, the mixture was concentrated in vacuo and applied on a column of silica gel. The product was eluted using 5% MeOH in CH_2Cl_2 to 10% MeOH in CH_2Cl_2 as eluent. Fractions containing the product were concentrated in vacuo. The product was resuspended in EtOAc, filtered and washed with Et_2O to obtain the product as a red-brown solid (38.0 mg, 58 µmol, 58%). **^{1}H NMR** (400 MHz, DMSO) δ 8.68 (d, *J* = 7.9 Hz, 2H), 7.94 (d, *J* = 7.5 Hz, 2H), 7.87 (t, *J* = 7.8 Hz, 2H), 7.43 – 7.32 (m, 4H), 7.26 (d, *J* = 5.3 Hz, 2H), 7.19 (dd, *J* = 8.0, 5.7 Hz, 2H), 3.67 (s, 6H). **HRMS:** (TOF-MS ES, m/z) calc. for $C_{24}H_{20}N_4O_6RuH$ $[M+H]^+$: 563.0511, found: 563.0514. **T_d**: 326 °C. **IR** (cm^{-1}) 3051, 2998, 2944, 2839, 1628, 1605, 1593, 1574, 1488, 1482, 1444, 1429, 1411, 1368, 1287, 1243, 1177, 1145, 1105, 1061, 1038, 1016, 940, 903, 882, 855. **UV/Vis:** ε_{360} = 9130 $M^{-1}\cdot cm^{-1}$.

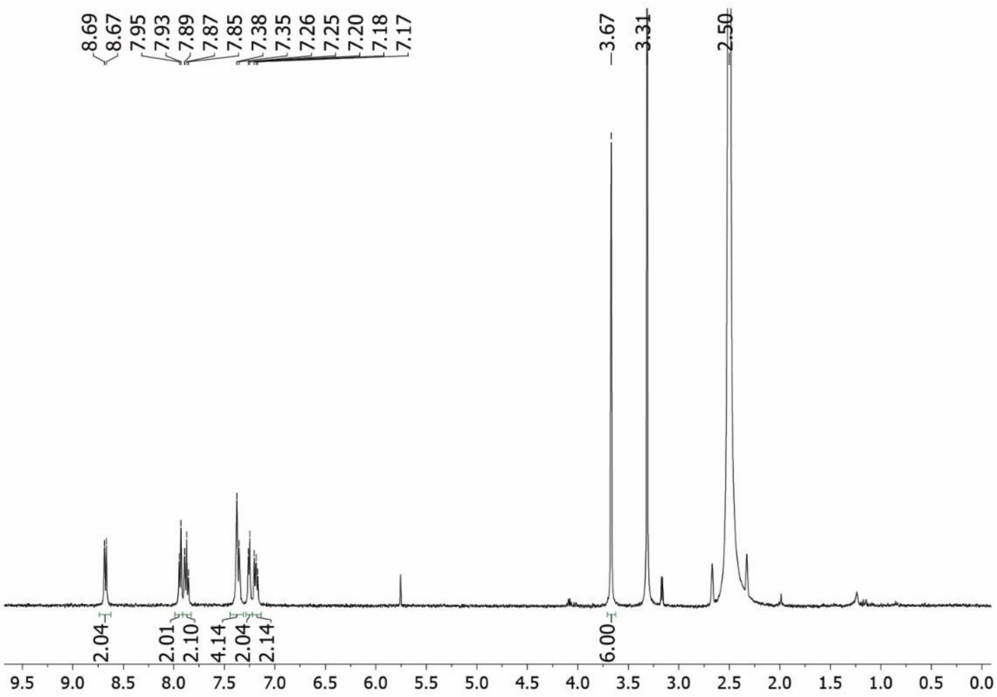

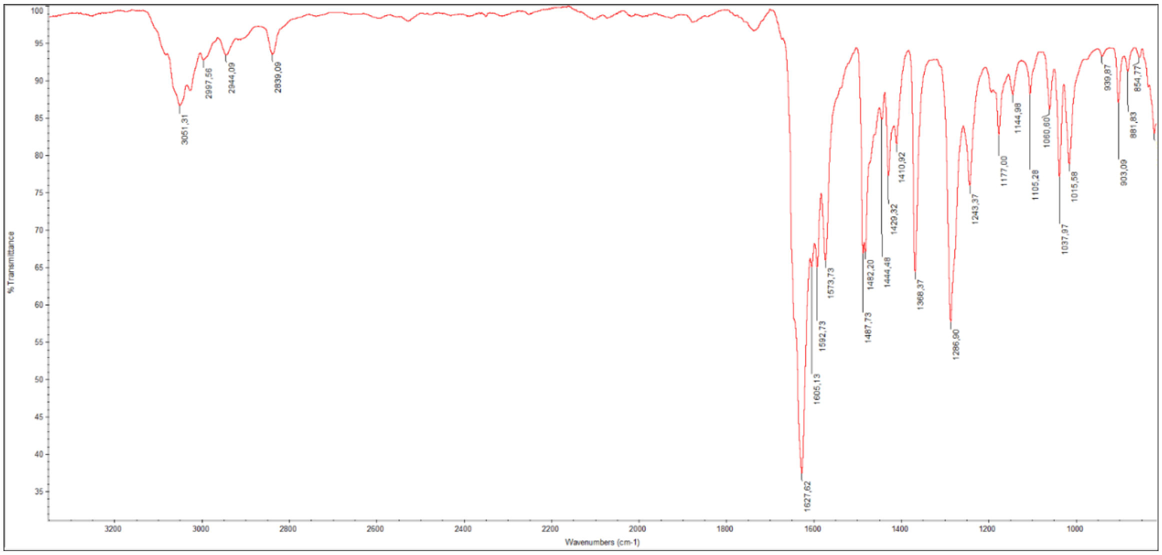

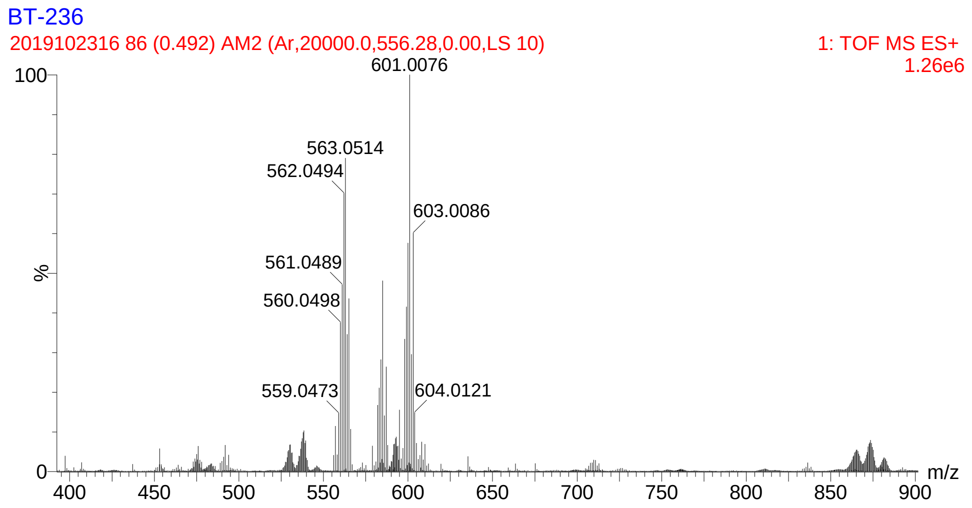

BT-236
2019102316 86 (0.492) AM2 (Ar,20000.0,556.28,0.00,LS 10)
1: TOF MS ES+
1.26e6
601.0076
563.0514
562.0494
603.0086
561.0489
560.0498
559.0473
604.0121
%
m/z

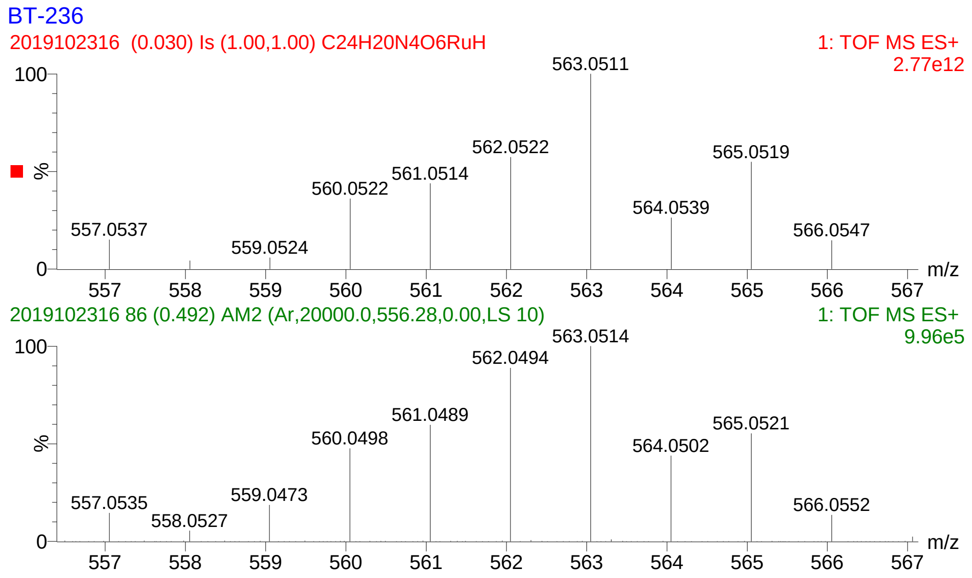

BT-236
2019102316 (0.030) Is (1.00,1.00) C24H20N4O6RuH
1: TOF MS ES+
2.77e12
563.0511
562.0522
565.0519
561.0514
560.0522
564.0539
557.0537
566.0547
559.0524
2019102316 86 (0.492) AM2 (Ar,20000.0,556.28,0.00,LS 10)
1: TOF MS ES+
9.96e5
563.0514
562.0494
561.0489
565.0521
560.0498
564.0502
559.0473
557.0535
566.0552
558.0527
%
m/z

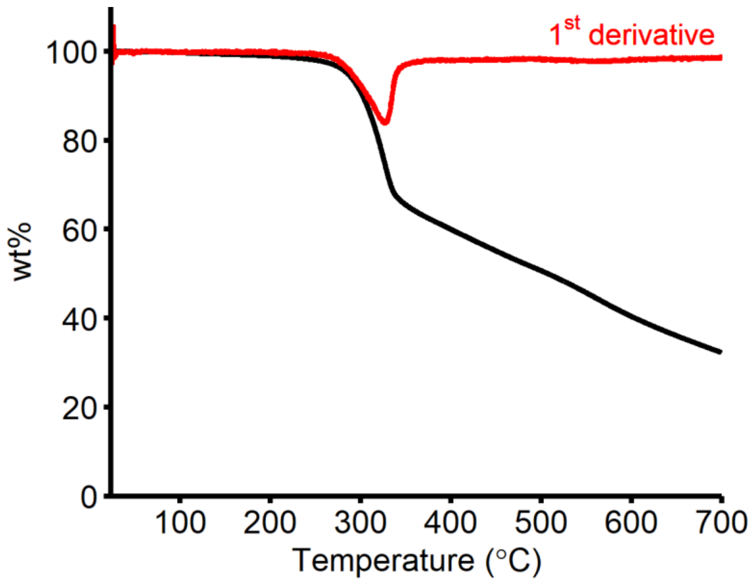

1st derivative
wt%
Temperature (°C)

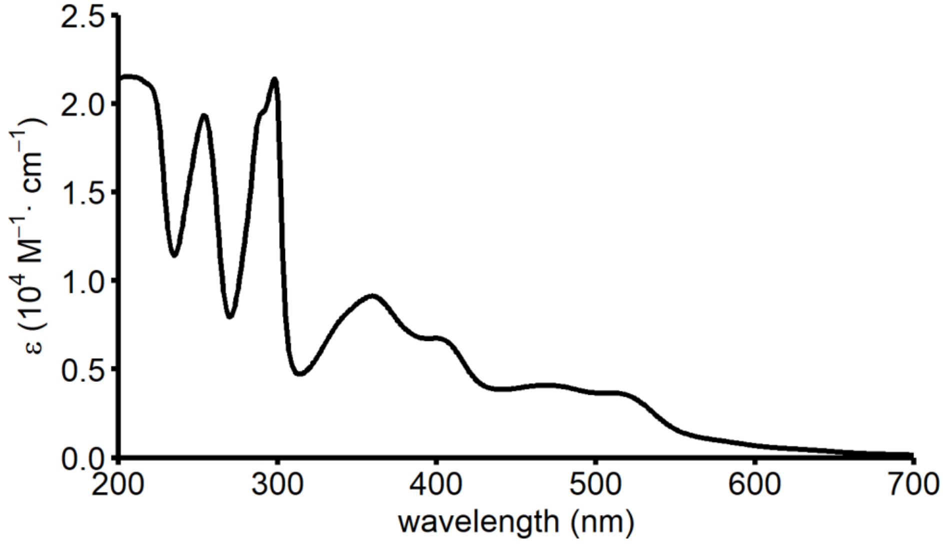

2.5
2.0
1.5
1.0
0.5
0.0
ε (10⁴ M⁻¹· cm⁻¹)
200
300
400
500
600
700
wavelength (nm)

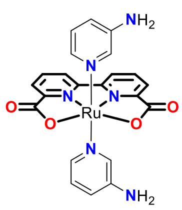

1-*m*NH$_2$: Pyridine ligand = 3-aminopyridine (10 eq., 1 mmol, 94 mg). After 17h reaction time, the mixture was filtered and washed with MeOH and Et_2O to obtain the product as a red-brown solid (30.5 mg, 57 µmol, 57%). **^{1}H NMR** (400 MHz, DMSO) δ 8.64 (d, *J* = 7.9 Hz, 1H), 7.87 (d, *J* = 7.5 Hz, 1H), 7.79 (t, *J* = 7.8 Hz, 1H), 7.32 (s, 1H), 6.80 – 6.65 (m, 2H), 6.49 – 6.37 (m, 1H), 5.58 (s, 2H). **HRMS:** (TOF-MS ES, m/z) calc. for $C_{22}H_{18}N_6O_4RuK$ $[M+K]^+$: 571.0076, found: 571.0078. **T*$_d$***: 355 °C. **IR** (cm^{-1}) 3421, 3324, 3205, 3063, 2970, 2940, 2817, 1599, 1492, 1449, 1412, 1369, 1319, 1301, 1270, 1184, 1162, 1149, 1107, 1030, 907, 870, 799, 775, 715, 706, 696, 687. **UV/Vis:** ε_{353} = 9088 M^{-1}·cm^{-1}.

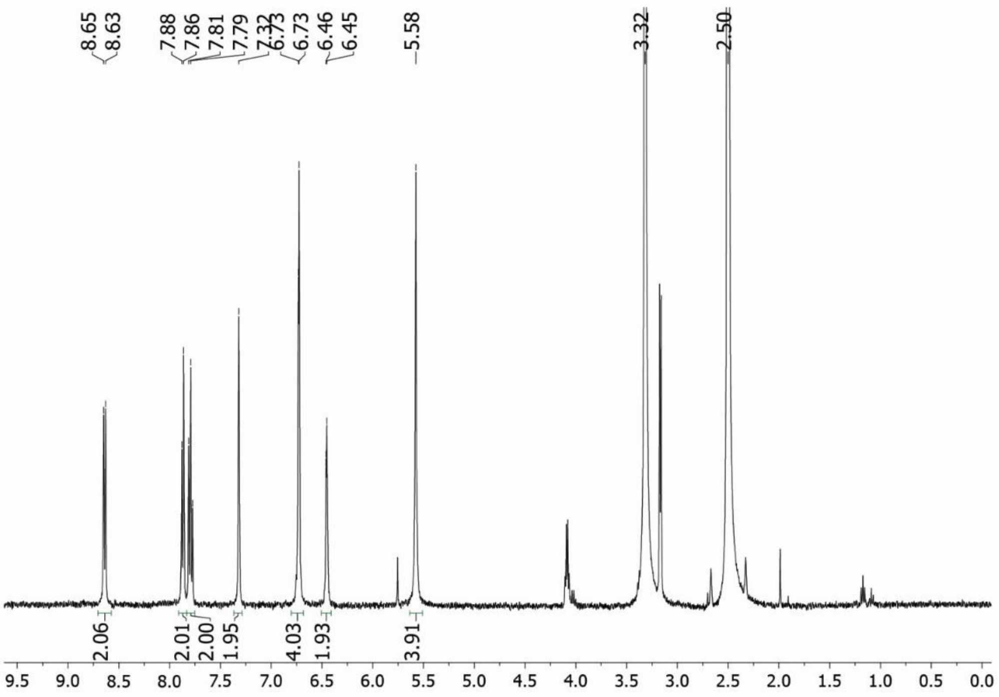

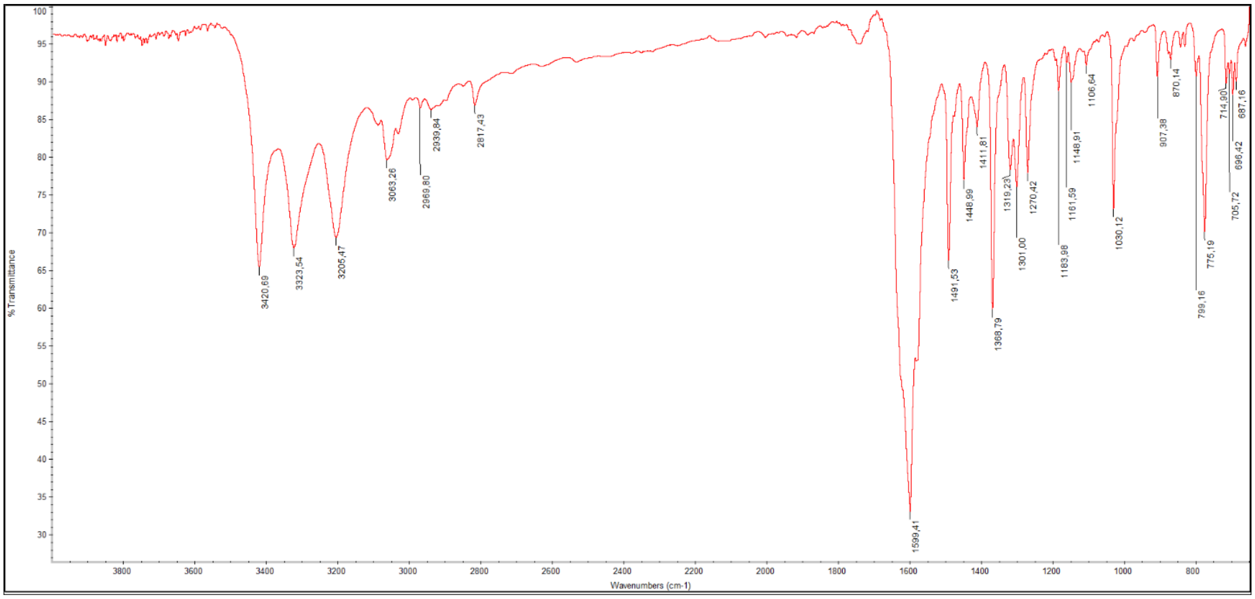

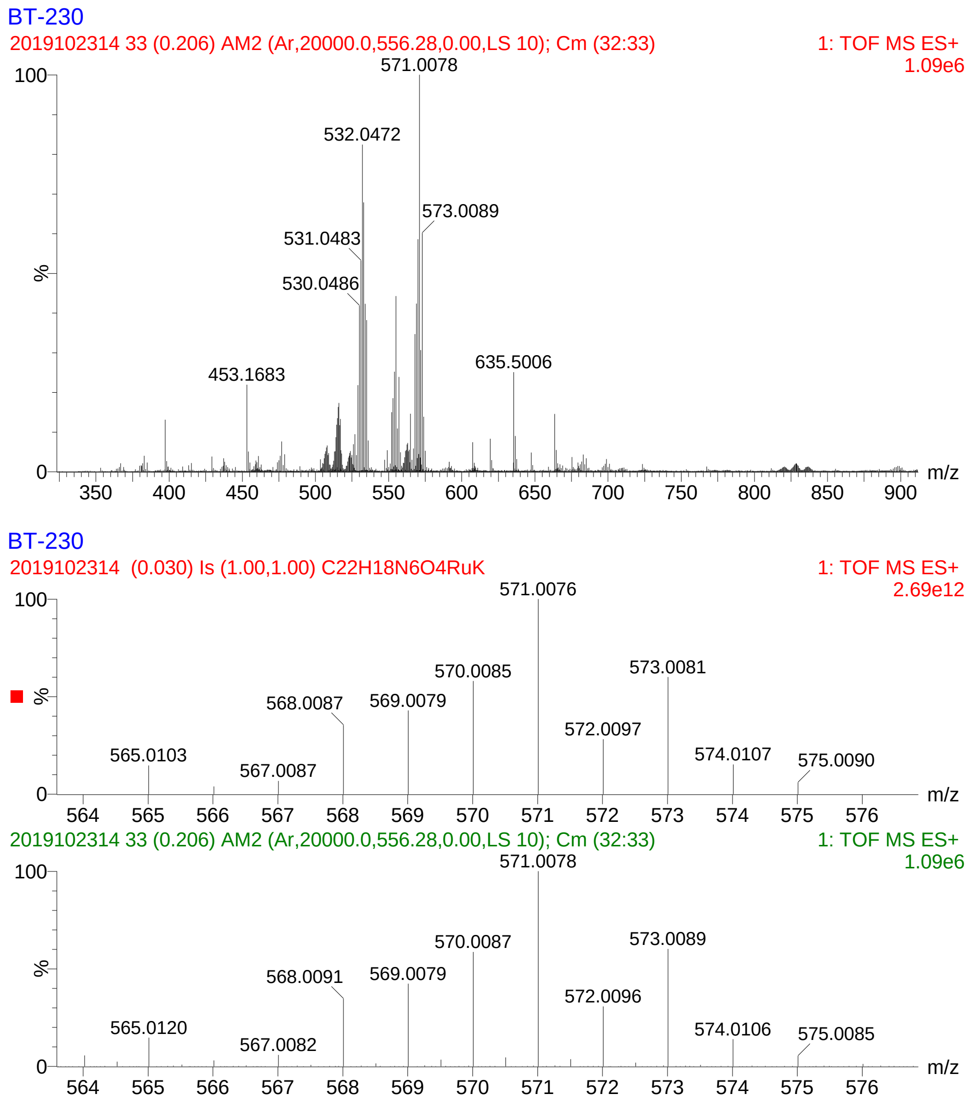

BT-230
2019102314 33 (0.206) AM2 (Ar,20000.0,556.28,0.00,LS 10); Cm (32:33)
1: TOF MS ES+
1.09e6
571.0078
532.0472
573.0089
531.0483
530.0486
453.1683
635.5006
100
%
0
m/z
350 400 450 500 550 600 650 700 750 800 850 900
BT-230
2019102314 (0.030) Is (1.00,1.00) C22H18N6O4RuK
1: TOF MS ES+
2.69e12
571.0076
570.0085
573.0081
568.0087
569.0079
572.0097
565.0103
567.0087
574.0107
575.0090
564 565 566 567 568 569 570 571 572 573 574 575 576
2019102314 33 (0.206) AM2 (Ar,20000.0,556.28,0.00,LS 10); Cm (32:33)
1: TOF MS ES+
1.09e6
571.0078
570.0087
573.0089
568.0091
569.0079
572.0096
565.0120
567.0082
574.0106
575.0085
564 565 566 567 568 569 570 571 572 573 574 575 576

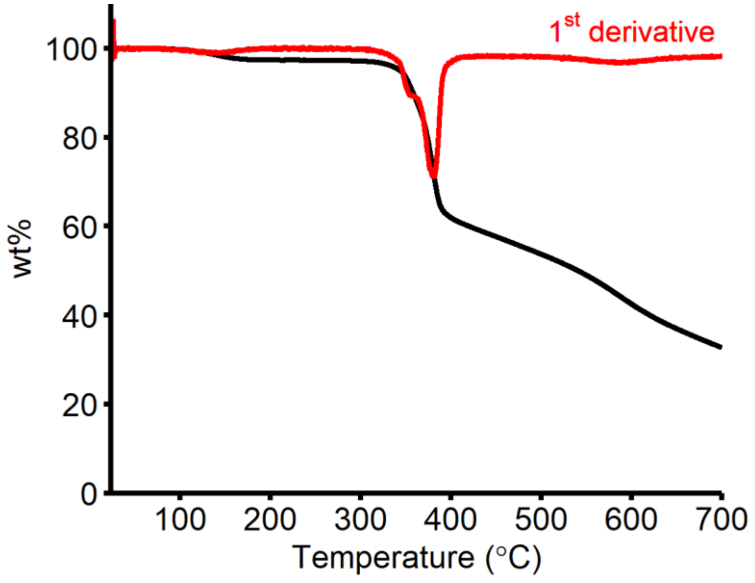

1st derivative
wt%
0 20 40 60 80 100
100 200 300 400 500 600 700
Temperature (°C)

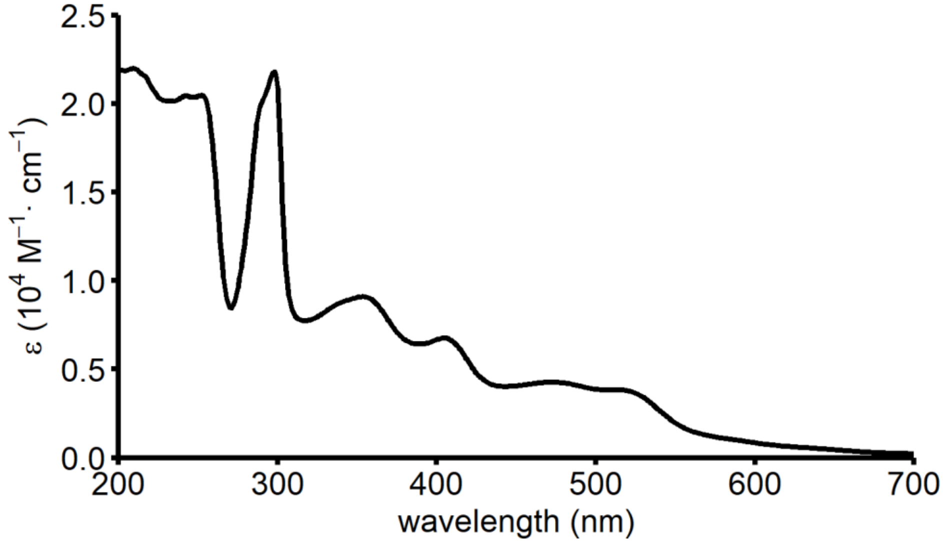
2.5
2.0
1.5
1.0
0.5
0.0
ε (10^4 M^−1 · cm^−1)
200
300
400
500
600
700
wavelength (nm)

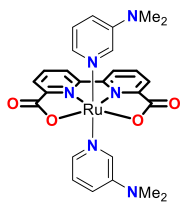

1-*m*NMe₂: Pyridine ligand = 3-dimethylaminopyridine (10 eq., 1 mmol, 122 mg). After 17h reaction time, the mixture was concentrated in vacuo and applied on a column of silica gel. The product was eluted using 5% MeOH in CH_2Cl_2 to 10% MeOH in CH_2Cl_2 as eluent. Fractions containing the product were concentrated in vacuo. The product was resuspended in EtOAc, filtered and washed with Et_2O to obtain the product as a red-brown solid (29.4 mg, 50 µmol, 50%). **^{1}H NMR** (400 MHz, DMSO) δ 8.63 (d, *J* = 7.8 Hz, 2H), 7.94 (d, *J* = 7.7 Hz, 2H), 7.83 (t, *J* = 7.7 Hz, 2H), 7.06 (s, 2H), 6.97 (s, 6H), 2.70 (s, 12H). **HRMS:** (TOF-MS ES, m/z) calc. for $C_{26}H_{26}N_6O_4RuK$ $[M+K]^+$: 627.0703, found: 627.0706. **T_d**: 341 °C. **IR** (cm^{-1}) 3528, 3434, 3082, 3052, 3026, 2992, 2884, 2811, 1628, 1590, 1568, 1505, 1445, 1430, 1410, 1369, 1315, 1292, 1238, 1196, 1178, 1148, 1066, 1020, 935, 852, 790, 772, 689. **UV/Vis:** ε_{343} = 12346 $M^{-1}\cdot cm^{-1}$.

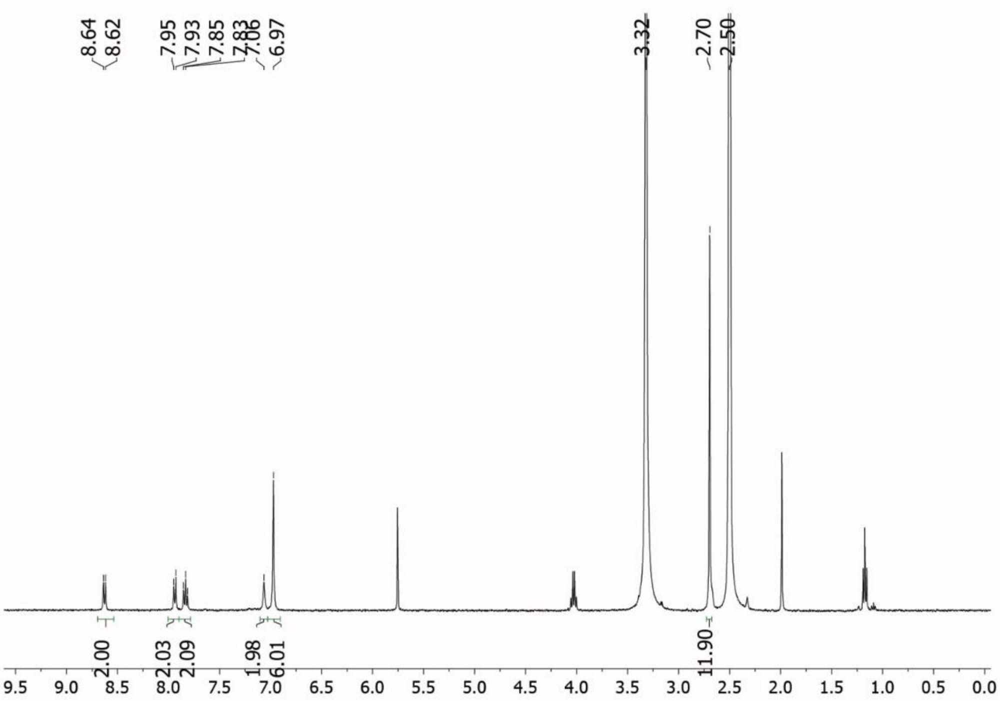

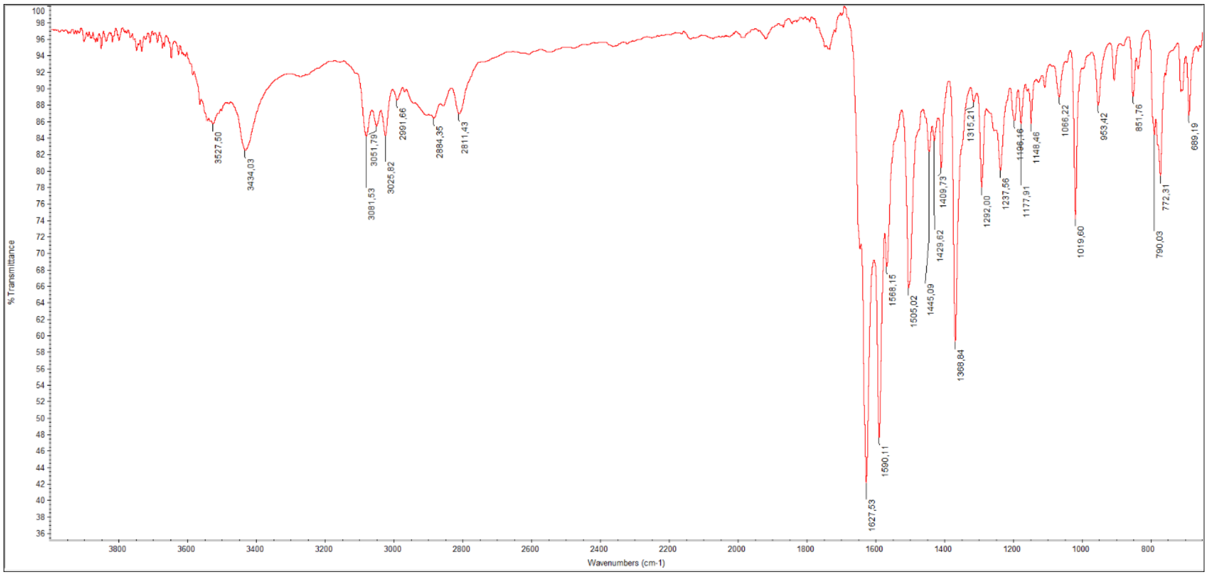

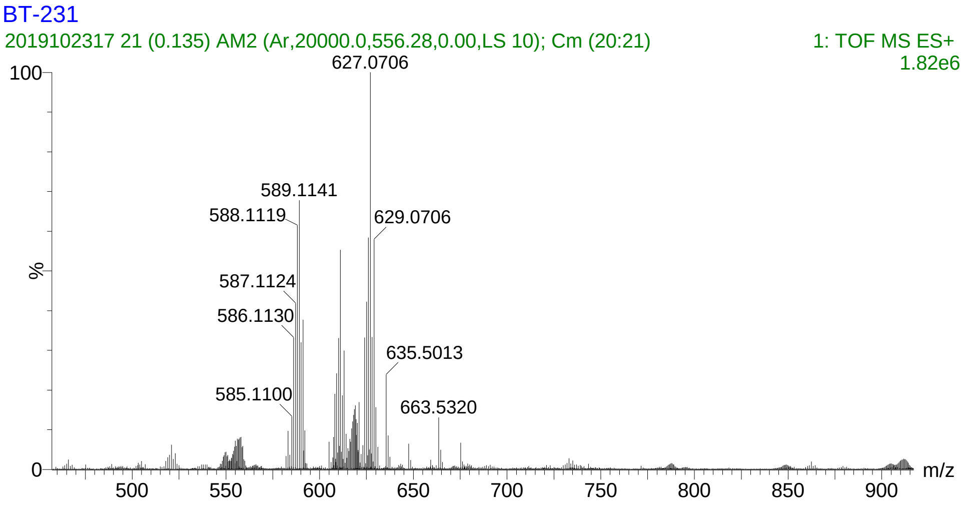
BT-231
2019102317 21 (0.135) AM2 (Ar,20000.0,556.28,0.00,LS 10); Cm (20:21)
1: TOF MS ES+
1.82e6
627.0706
589.1141
588.1119
629.0706
587.1124
586.1130
635.5013
585.1100
663.5320
%
m/z

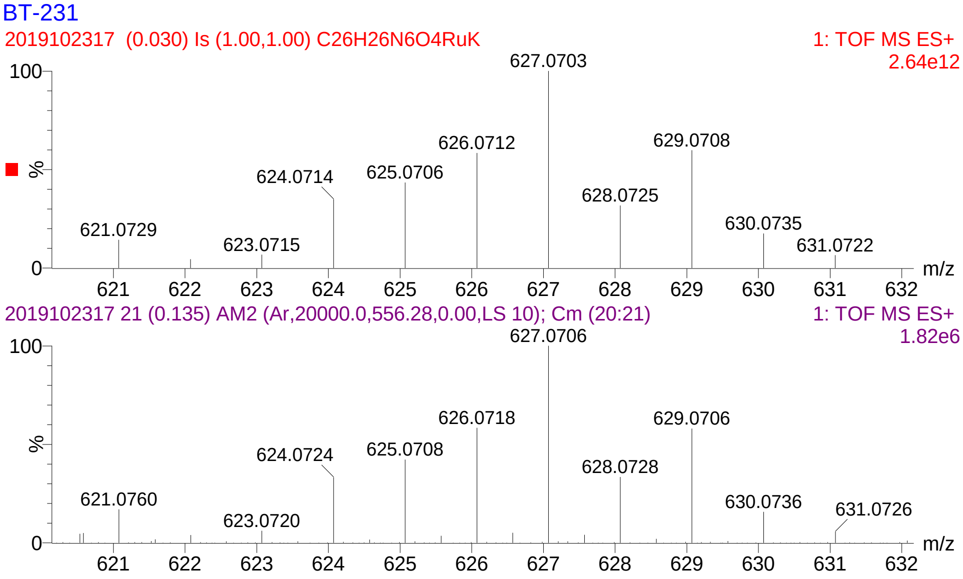
BT-231
2019102317 (0.030) Is (1.00,1.00) C26H26N6O4RuK
1: TOF MS ES+
2.64e12
627.0703
626.0712
629.0708
624.0714
625.0706
628.0725
621.0729
630.0735
623.0715
631.0722
2019102317 21 (0.135) AM2 (Ar,20000.0,556.28,0.00,LS 10); Cm (20:21)
1: TOF MS ES+
1.82e6
627.0706
626.0718
629.0706
624.0724
625.0708
628.0728
621.0760
630.0736
631.0726
623.0720
%
m/z

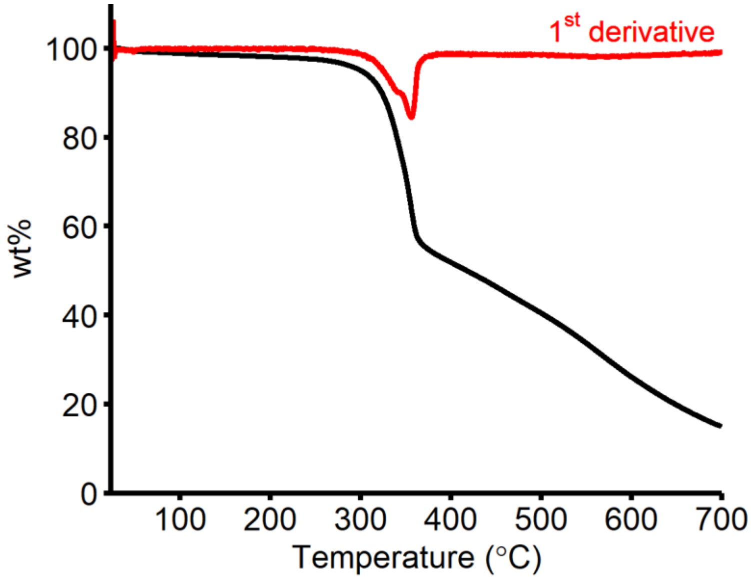
1st derivative
wt%
Temperature (°C)

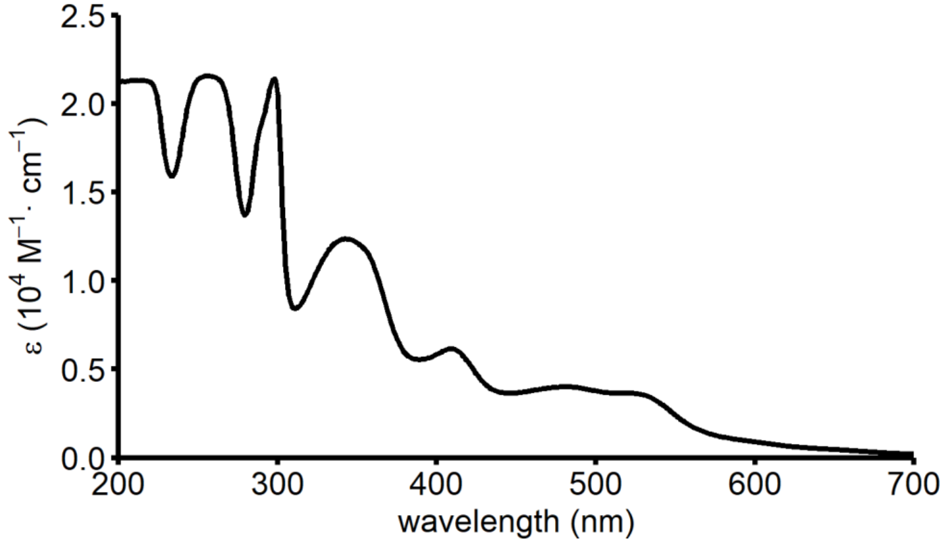

2.5
2.0
1.5
1.0
0.5
0.0
ε (10⁴ M⁻¹· cm⁻¹)
200
300
400
500
600
700
wavelength (nm)

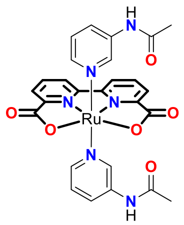

1-*m*NHAc: Pyridine ligand = *N*-(pyridin-3-yl)acetamide (2.5 eq., 0.25 mmol, 34 mg). After 16h reaction time the product was filtered off and washed with MeOH, EtOAc and Et_2O to obtain the product as a red-brown solid (25.6 mg, 42 µmol, 42%). **^{1}H NMR** (400 MHz, DMSO) δ 10.23 (s, 2H), 8.72 (d, *J* = 7.2 Hz, 2H), 8.07 (s, 2H), 7.92 – 7.81 (m, 4H), 7.76 (d, *J* = 8.5 Hz, 2H), 7.45 (d, *J* = 5.5 Hz, 2H), 7.17 (dd, *J* = 7.6, 5.3 Hz, 2H), 1.96 (s, 6H). **HRMS:** (TOF-MS ES, m/z) calc. for $C_{26}H_{21}N_6O_6Ru$ $[M-H]^-$: 615.0573, found: 615.0571. **T_d**: 360 °C. **IR** (cm^{-1}) 3312, 3258, 3186, 3139, 3118, 3071, 2955, 1703, 1694, 1614, 1589, 1558, 1543, 1485, 1418, 1373, 1329, 1307, 1284, 1246, 1191, 1162, 1109, 1023, 955, 908, 833, 799, 778, 715, 706, 694. **UV/Vis:** ε_{360} = 8772 $M^{-1}\cdot cm^{-1}$.

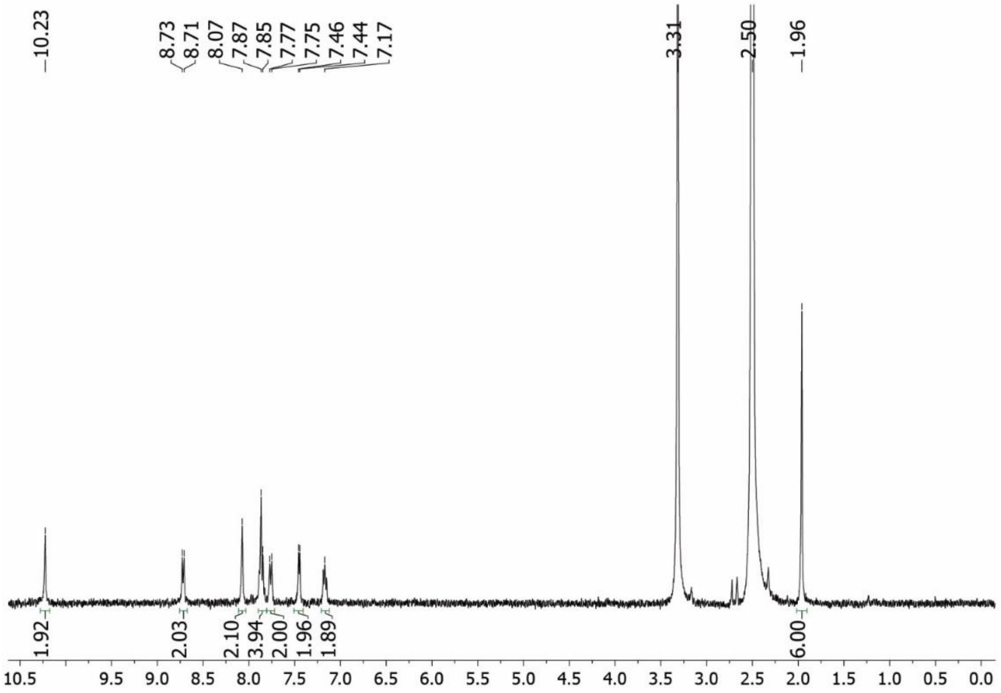

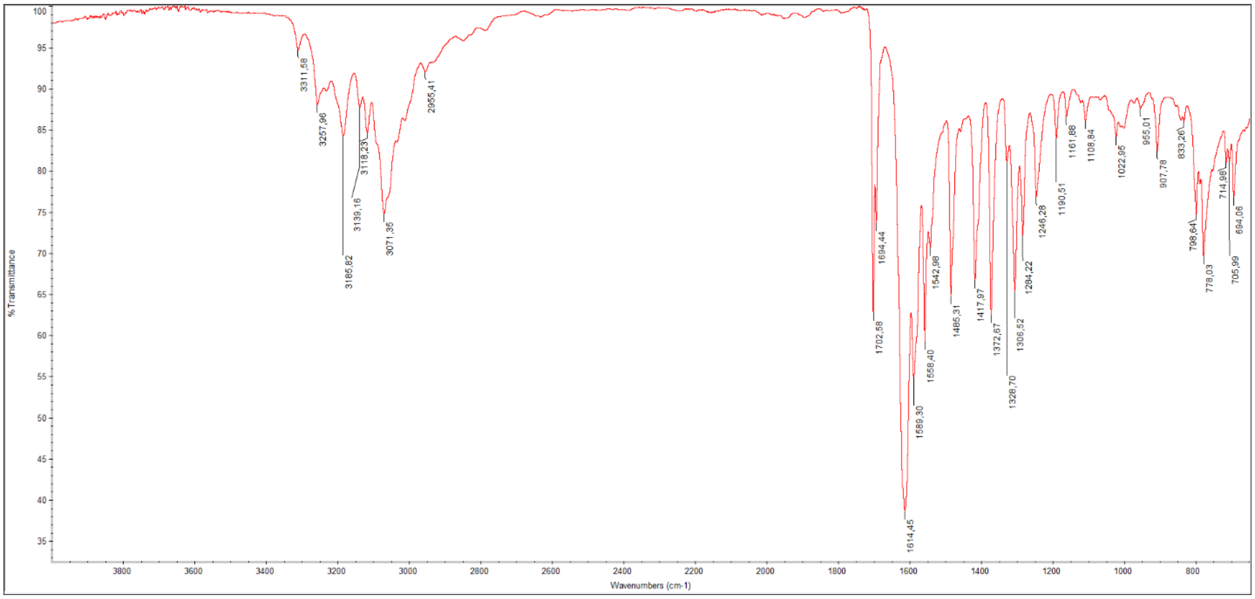

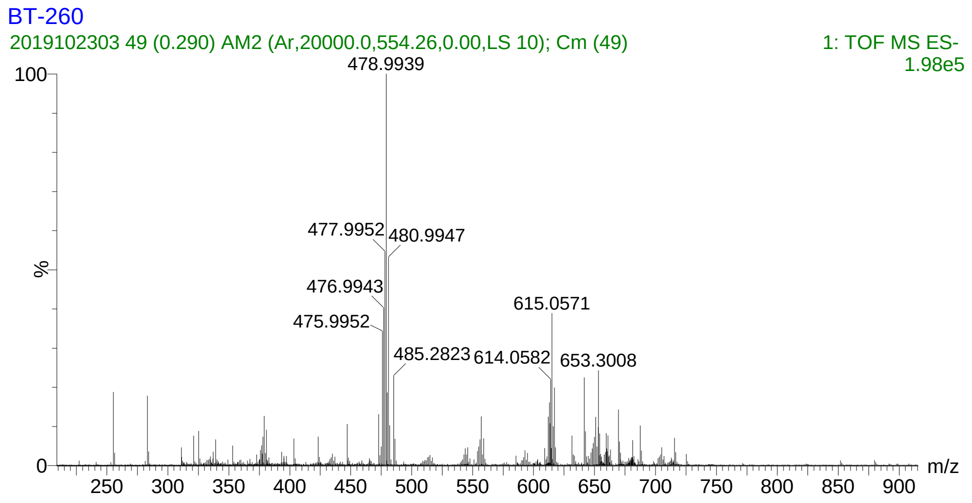
BT-260
2019102303 49 (0.290) AM2 (Ar,20000.0,554.26,0.00,LS 10); Cm (49)
1: TOF MS ES-
1.98e5
478.9939
477.9952
480.9947
476.9943
475.9952
485.2823
615.0571
614.0582
653.3008
%
m/z

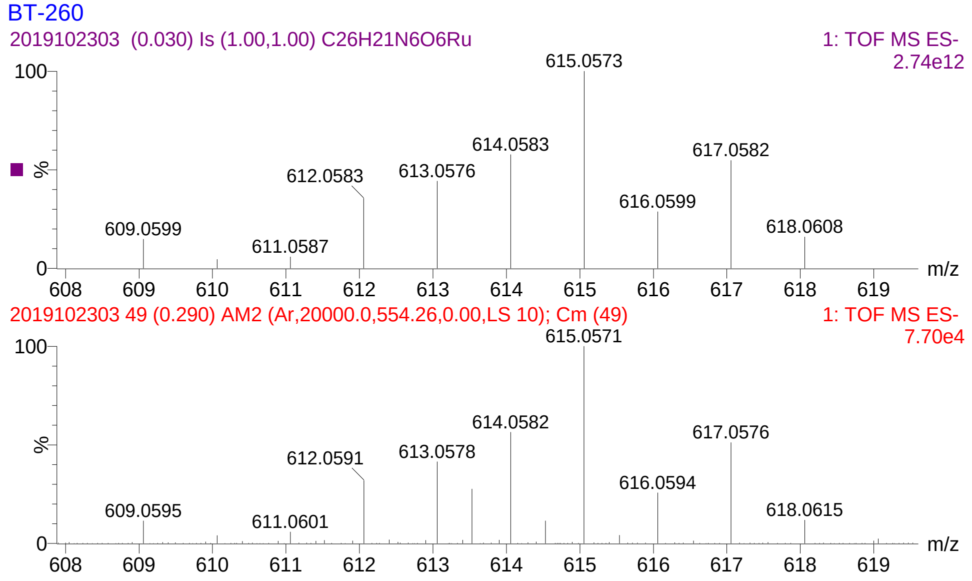
BT-260
2019102303 (0.030) Is (1.00,1.00) C26H21N6O6Ru
1: TOF MS ES-
2.74e12
615.0573
614.0583
617.0582
612.0583
613.0576
616.0599
609.0599
618.0608
611.0587
%
m/z
2019102303 49 (0.290) AM2 (Ar,20000.0,554.26,0.00,LS 10); Cm (49)
1: TOF MS ES-
7.70e4
615.0571
614.0582
617.0576
612.0591
613.0578
616.0594
609.0595
618.0615
611.0601
%
m/z

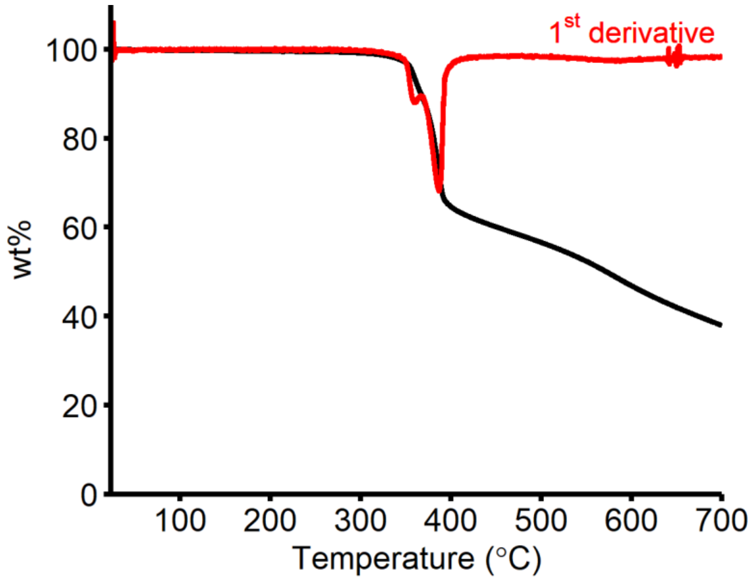
1st derivative
wt%
Temperature (°C)

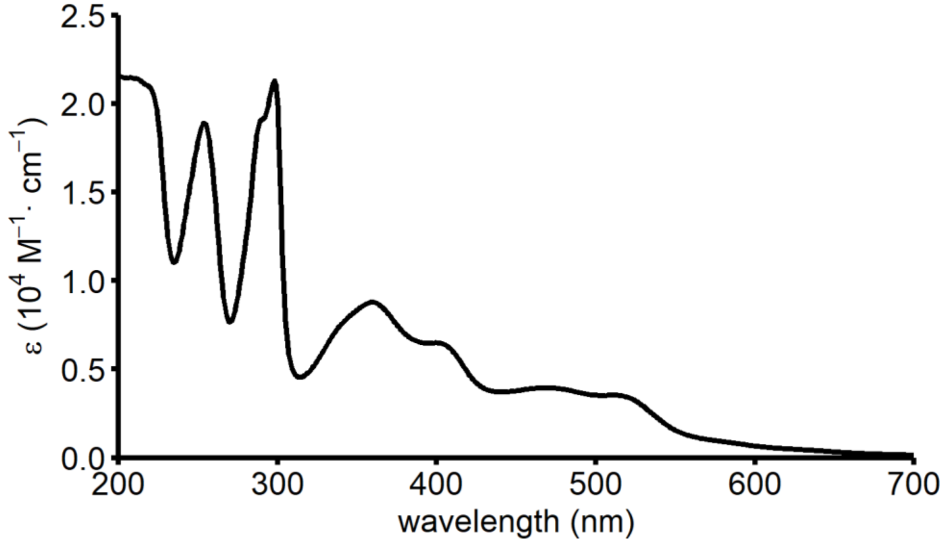
2.5
2.0
1.5
1.0
0.5
0.0
ε (10⁴ M⁻¹· cm⁻¹)
200
300
400
500
600
700
wavelength (nm)

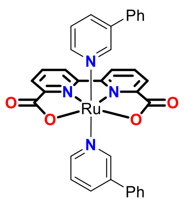

1-*m*Ph: Pyridine ligand = 3-phenylpyridine (10 eq., 1 mmol, 155 mg, 142 µL). After 17.5h reaction time, the mixture was concentrated in vacuo and applied on a column of silica gel. The product was eluted using 5% MeOH in CH_2Cl_2 to 10% MeOH in CH_2Cl_2 as eluent. Fractions containing the product were concentrated in vacuo. The product was resuspended in EtOAc, filtered and washed with Et_2O to obtain the product as a red-brown solid (38.0 mg, 58 µmol, 58%). **^{1}H NMR** (400 MHz, DMSO) δ 8.72 (d, *J* = 7.7 Hz, 2H), 8.23 (s, 2H), 8.02 (d, *J* = 8.1 Hz, 2H), 7.97 (d, *J* = 7.1 Hz, 2H), 7.92 (t, *J* = 7.7 Hz, 2H), 7.57 – 7.41 (m, 12H), 7.33 (dd, *J* = 7.8, 5.8 Hz, 2H). **HRMS:** (TOF-MS ES, m/z) calc. for $C_{34}H_{24}N_4O_4RuH$ $[M+H]^+$: 655.0928, found: 655.0929. **T_d**: 320 °C. **IR** (cm^{-1}) 3050, 1738, 1636, 1598, 1468, 1454, 1409, 1367, 1299, 1285, 1250, 1186, 1175, 1159, 1146, 1106, 1079, 1034, 1022, 903, 846, 834, 821, 781, 772, 755, 697. **UV/Vis:** ε_{370} = 11075 $M^{-1}\cdot cm^{-1}$.

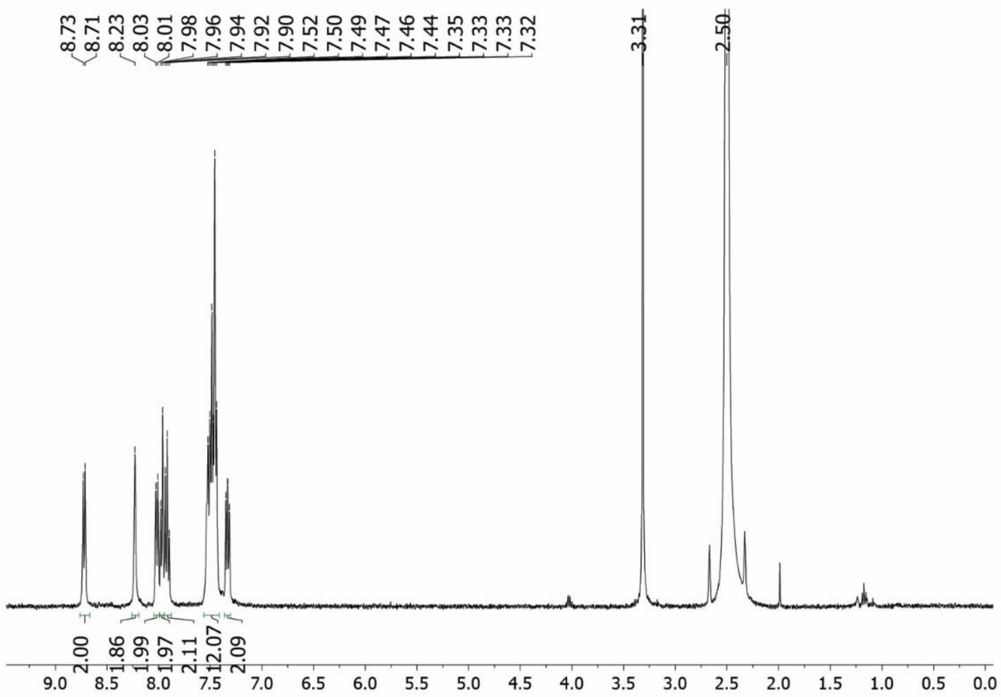

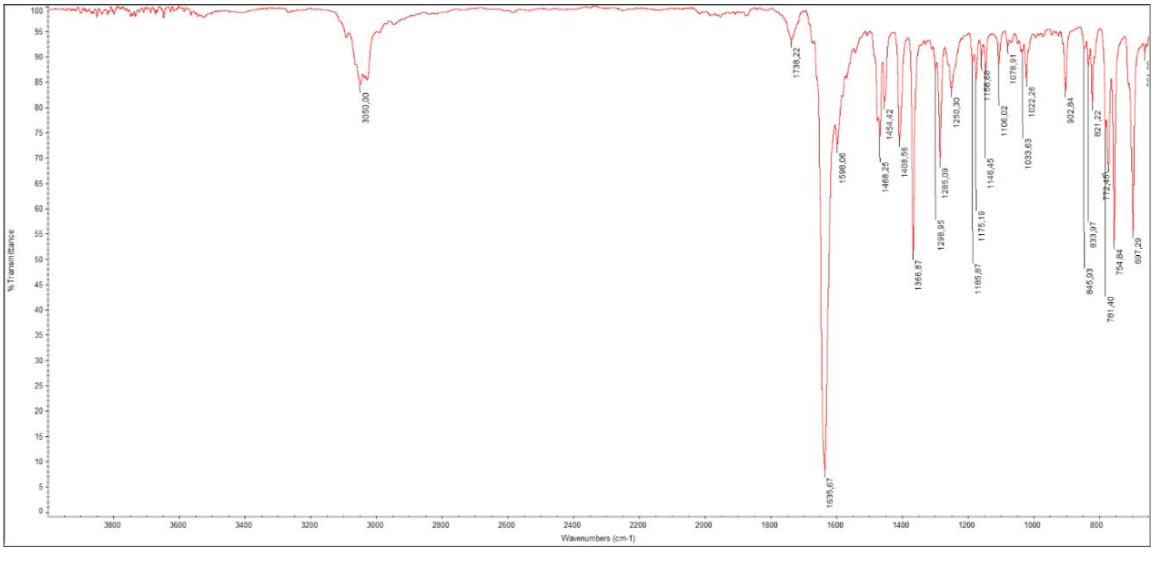

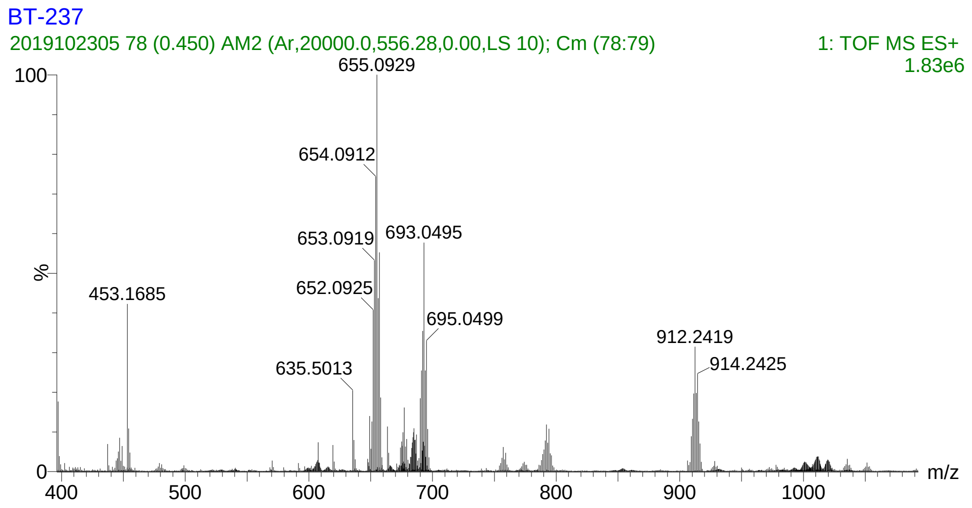
BT-237
2019102305 78 (0.450) AM2 (Ar,20000.0,556.28,0.00,LS 10); Cm (78:79)
1: TOF MS ES+
1.83e6
655.0929
654.0912
653.0919
693.0495
452.1685
652.0925
695.0499
912.2419
914.2425
635.5013
%
m/z

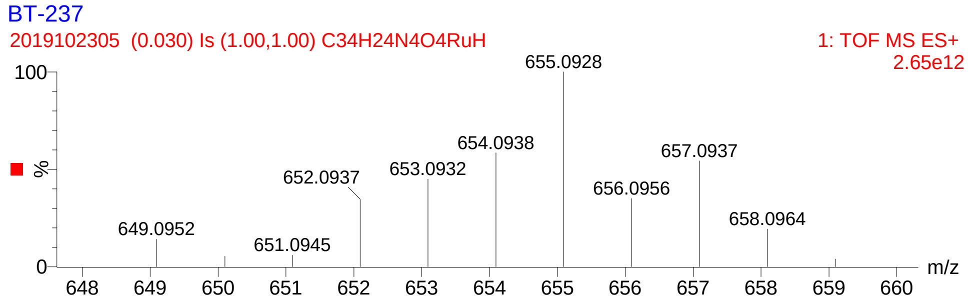
BT-237
2019102305 (0.030) Is (1.00,1.00) C34H24N4O4RuH
1: TOF MS ES+
2.65e12
655.0928
654.0938
657.0937
652.0937
653.0932
656.0956
649.0952
658.0964
651.0945
%
m/z

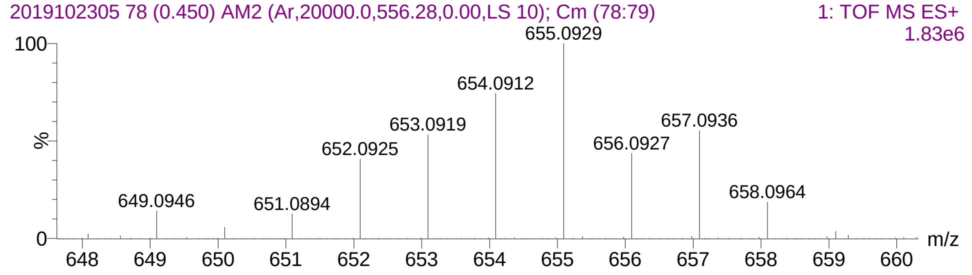
2019102305 78 (0.450) AM2 (Ar,20000.0,556.28,0.00,LS 10); Cm (78:79)
1: TOF MS ES+
1.83e6
655.0929
654.0912
657.0936
653.0919
652.0925
656.0927
649.0946
651.0894
658.0964
%
m/z

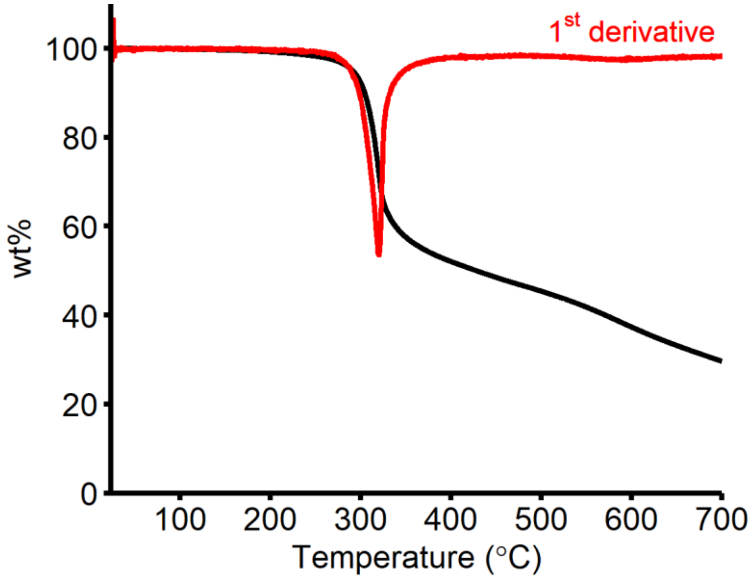
1st derivative
wt%
Temperature (°C)

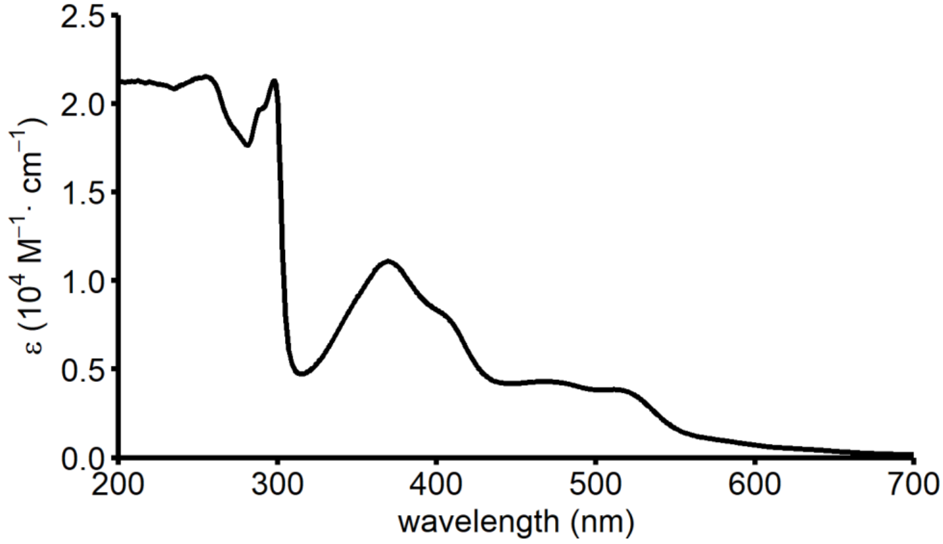

2.5
2.0
1.5
1.0
0.5
0.0
ε (10^4 M^-1 · cm^-1)
200
300
400
500
600
700
wavelength (nm)

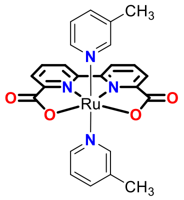

1-*m*Me: Pyridine ligand = 3-picoline (10 eq., 1 mmol, 93 mg, 83 µL). After 20.5h reaction time, the mixture was concentrated in vacuo and applied on a column of silica gel. The product was eluted using 5% MeOH in CH_2Cl_2 to 10% MeOH in CH_2Cl_2 as eluent. Fractions containing the product were concentrated in vacuo. The product was resuspended in EtOAc, filtered and washed with Et_2O to obtain the product as a red-brown solid (23.0 mg, 43 µmol, 43%). **^{1}H NMR** (400 MHz, DMSO) δ 8.70 (d, *J* = 8.1 Hz, 2H), 7.94 – 7.83 (m, 4H), 7.80 (s, 2H), 7.52 (d, *J* = 8.0 Hz, 2H), 7.21 (d, *J* = 5.5 Hz, 2H), 7.09 (dd, *J* = 7.5, 6.0 Hz, 2H), 2.15 (s, 6H). **HRMS:** (TOF-MS ES, m/z) calc. for $C_{24}H_{20}N_4O_4RuH$ $[M+H]^+$: 531.0601, found: 531.0614. **T_d**: 330 °C. **IR** (cm^{-1}) 3075, 2922, 1633, 1575, 1477, 1456, 1397, 1359, 1302, 1286, 1254, 1179, 1168, 1146, 1104, 1044, 1025, 904, 794, 773, 712, 705, 695. **UV/Vis:** ε_{358} = 8911 $M^{-1}\cdot cm^{-1}$.

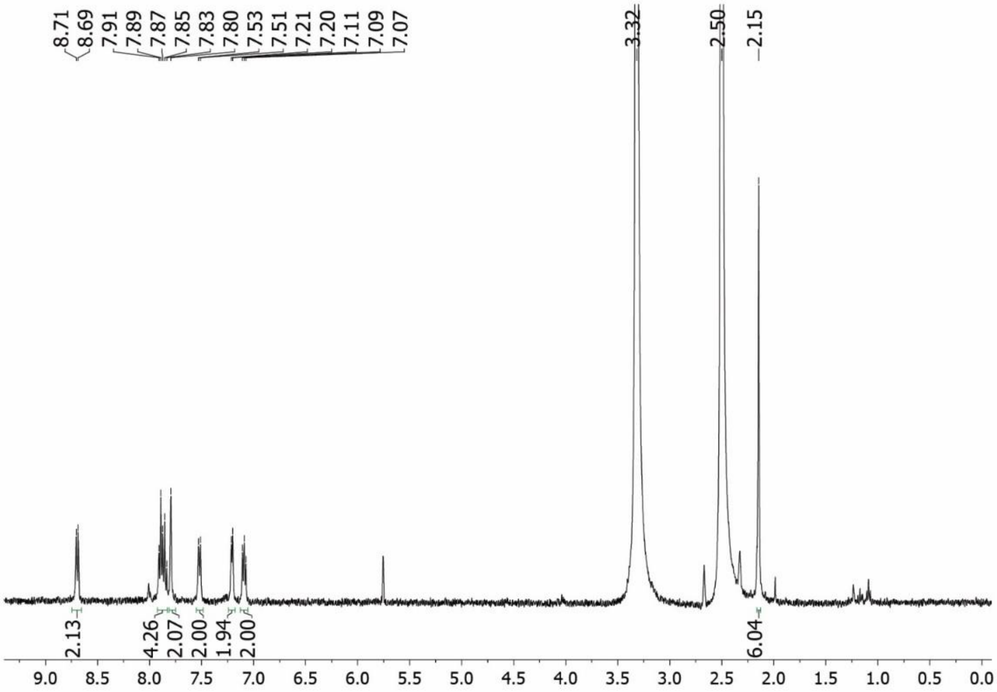

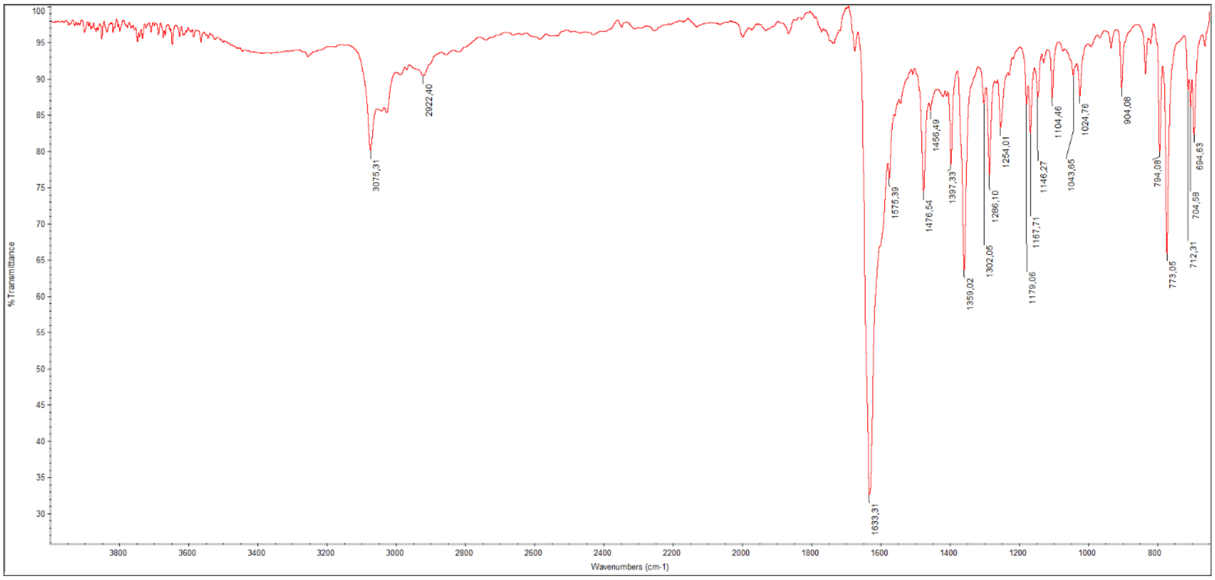

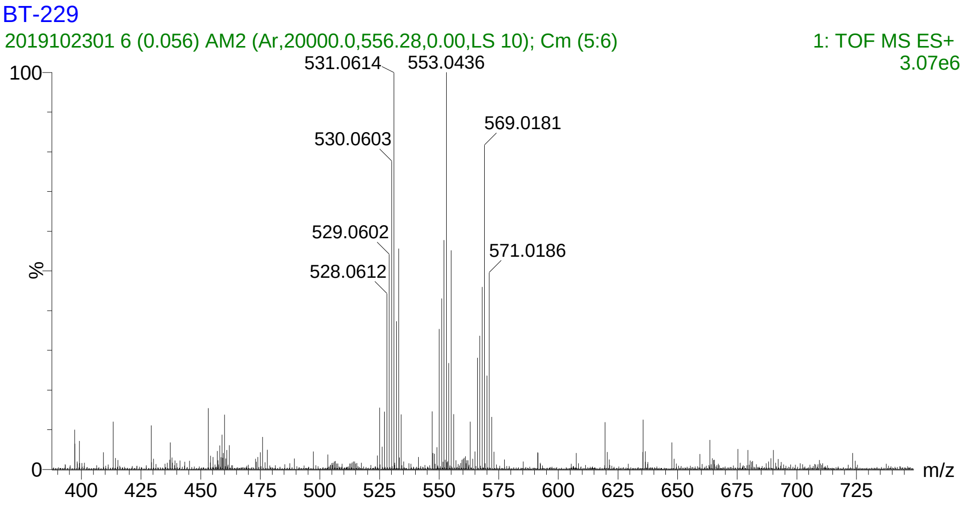
BT-229
2019102301 6 (0.056) AM2 (Ar,20000.0,556.28,0.00,LS 10); Cm (5:6)
1: TOF MS ES+
3.07e6
531.0614
553.0436
530.0603
569.0181
529.0602
571.0186
528.0612
%
m/z

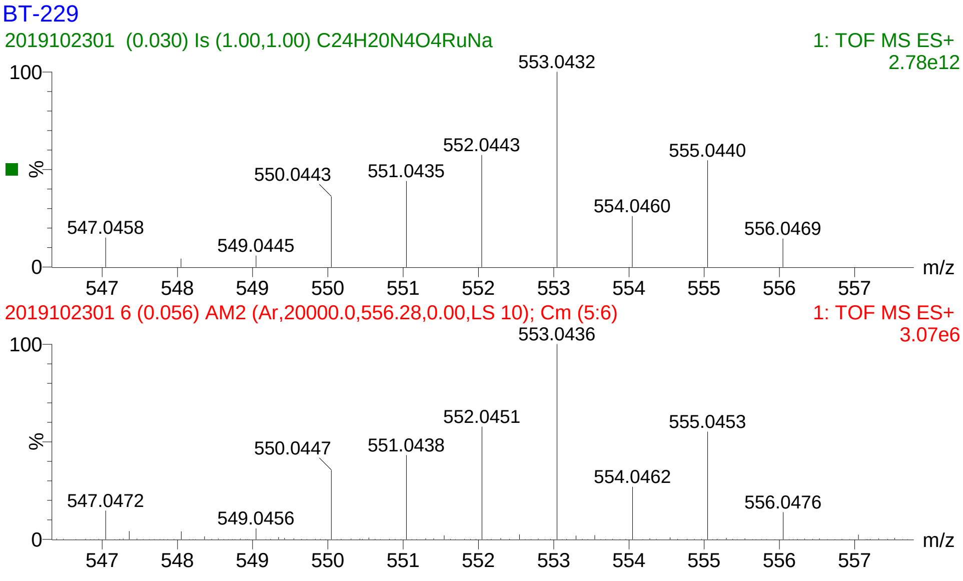
BT-229
2019102301 (0.030) Is (1.00,1.00) C24H20N4O4RuNa
1: TOF MS ES+
2.78e12
553.0432
552.0443
555.0440
550.0443
551.0435
554.0460
547.0458
556.0469
549.0445
%
m/z
2019102301 6 (0.056) AM2 (Ar,20000.0,556.28,0.00,LS 10); Cm (5:6)
1: TOF MS ES+
3.07e6
553.0436
552.0451
555.0453
550.0447
551.0438
554.0462
547.0472
556.0476
549.0456
%
m/z

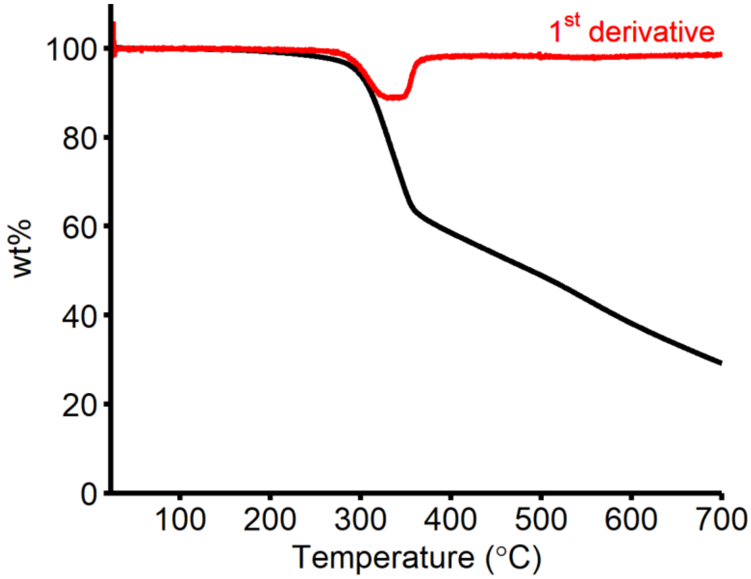
1st derivative
wt%
Temperature (°C)

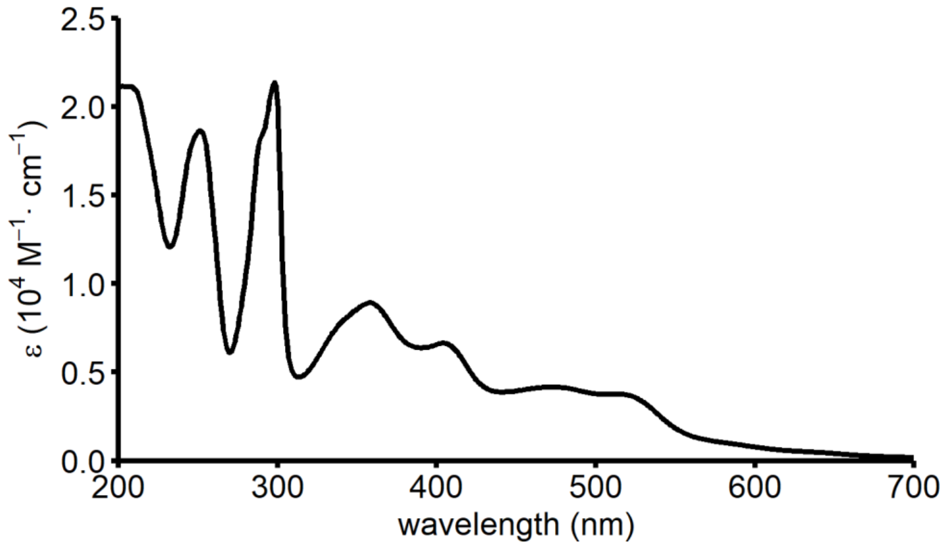
2.5
2.0
1.5
1.0
0.5
0.0
ε (10^4 M^{-1}· cm^{-1})
200
300
400
500
600
700
wavelength (nm)

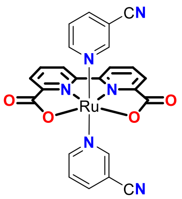

1-*m*CN: Pyridine ligand = 3-pyridinecarbonitrile (2.5 eq., 0.25 mmol, 26 mg). After 16h reaction time the product was filtered off and washed with MeOH, EtOAc and Et_2O to obtain the product as a red-brown solid (9.7 mg, 18 µmol, 18%). **^{1}H NMR** (400 MHz, DMSO) δ 8.75 (d, *J* = 7.3 Hz, 2H), 8.27 – 8.20 (m, 4H), 7.98 – 7.90 (m, 6H), 7.49 – 7.43 (m, 2H). **HRMS:** (TOF-MS ES, m/z) calc. for $C_{24}H_{14}N_6O_4RuK$ $[M+K]^+$: 590.9763, found: 590.9761. **T*d***: 336 °C. **IR** (cm^{-1}) 3052, 3032, 2239, 1635, 1569, 1471, 1418, 1405, 1362, 1301, 1282, 1249, 1212, 1185, 1170, 1144, 1105, 1042, 1022, 903, 838, 830, 817, 772, 706, 687. **UV/Vis:** ε_{401} = 11833 M^{-1}·cm^{-1}.

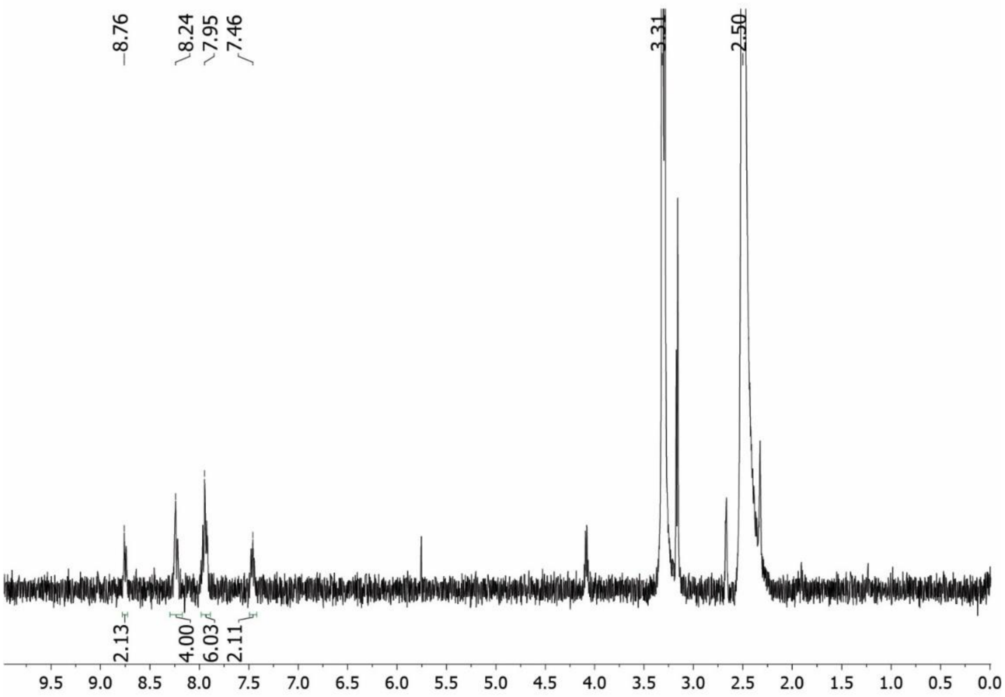

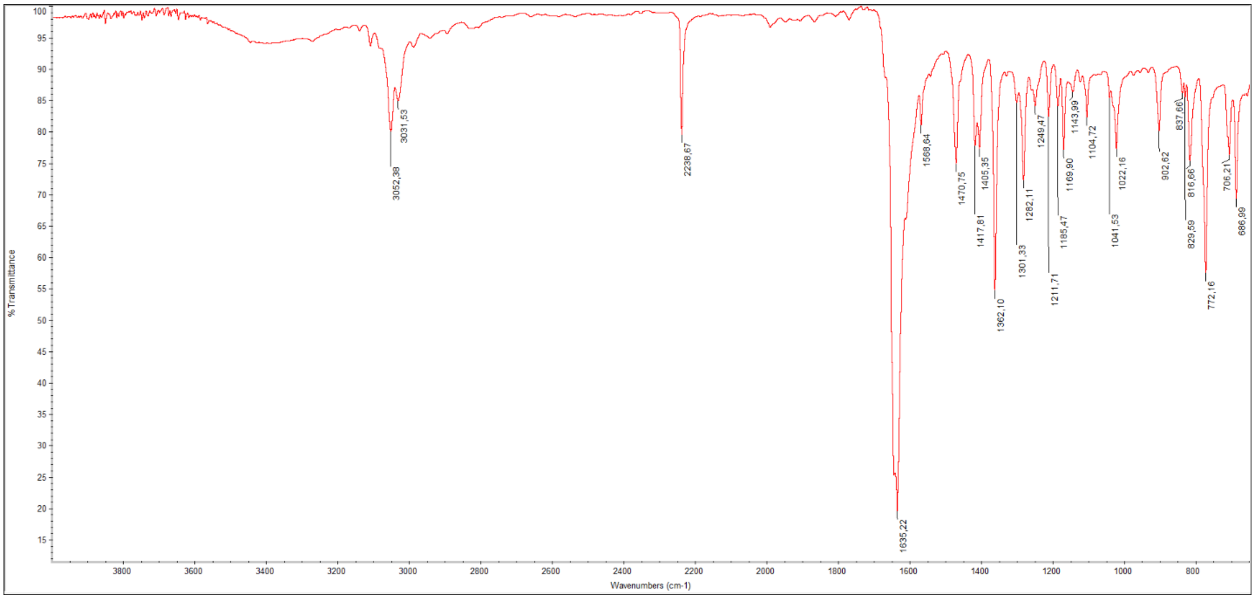

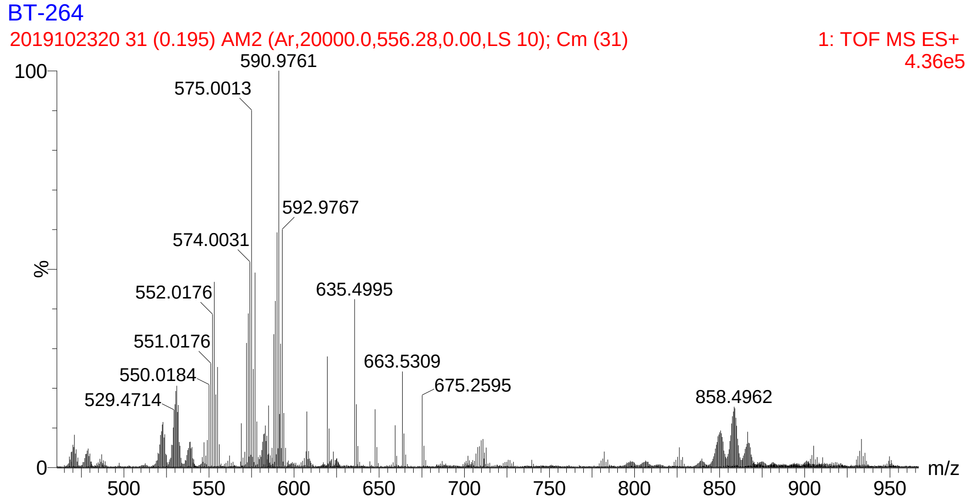
BT-264
2019102320 31 (0.195) AM2 (Ar,20000.0,556.28,0.00,LS 10); Cm (31)
1: TOF MS ES+
4.36e5
590.9761
575.0013
592.9767
574.0031
552.0176
635.4995
551.0176
663.5309
550.0184
675.2595
529.4714
858.4962
%
m/z

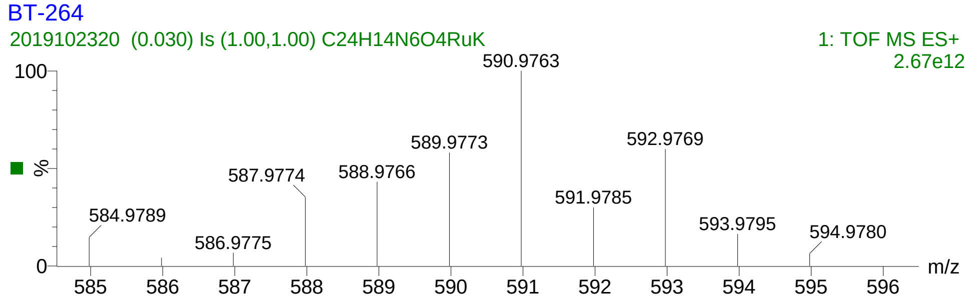
BT-264
2019102320 (0.030) Is (1.00,1.00) C24H14N6O4RuK
1: TOF MS ES+
2.67e12
590.9763
589.9773
592.9769
587.9774
588.9766
591.9785
584.9789
593.9795
586.9775
594.9780
%
m/z

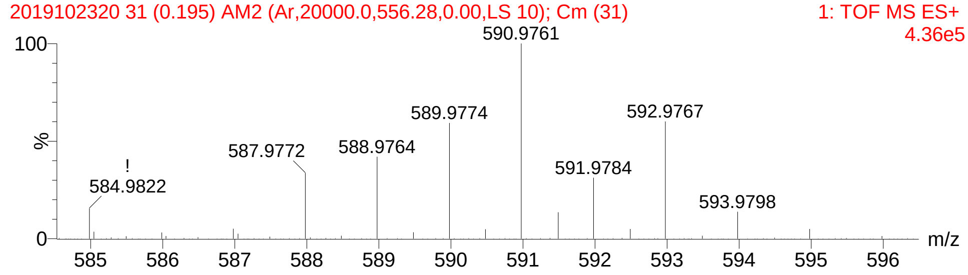
2019102320 31 (0.195) AM2 (Ar,20000.0,556.28,0.00,LS 10); Cm (31)
1: TOF MS ES+
4.36e5
590.9761
589.9774
592.9767
587.9772
588.9764
591.9784
584.9822
593.9798
%
m/z

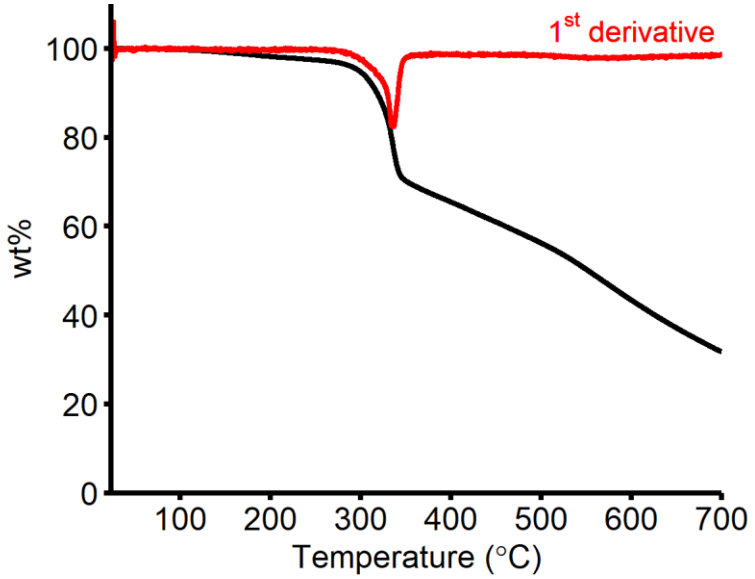
1st derivative
wt%
Temperature (°C)

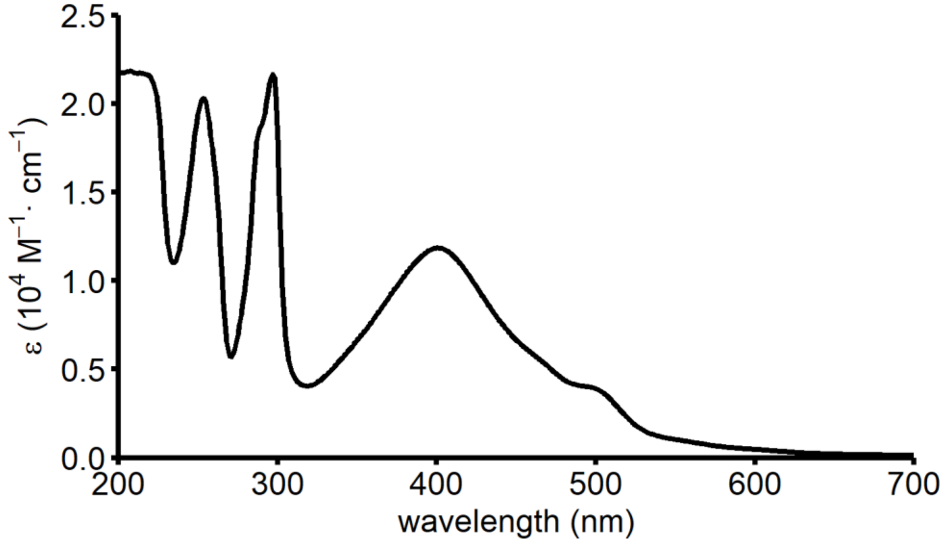
ε (10^4 M^-1 · cm^-1)
2.5
2.0
1.5
1.0
0.5
0.0
200
300
400
500
600
700
wavelength (nm)

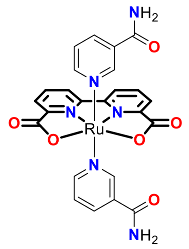

1-*m*CONH₂: Pyridine ligand = nicotinamide (2.5 eq., 0.25 mmol, 31 mg). After 16h reaction time the product was filtered off and washed with MeOH, EtOAc and Et_2O to obtain the product as a red-brown solid (28 mg, 47 µmol, 47%).[1]**H NMR** (400 MHz, DMSO) δ 8.75 (d, *J* = 4.8 Hz, 2H), 8.37 (br. s, 2H), 8.19 (s, 2H), 8.10 (d, *J* = 7.5 Hz, 2H), 7.96 – 7.85 (m, 4H), 7.72 (s, 2H), 7.66 (s, 2H), 7.33 (t, *J* = 6.7 Hz, 2H). **HRMS:** (MALDI TOF-MS, m/z) calc. for $C_{24}H_{18}N_6O_6Ru^+$ $[M]^+$: 588.0326, found: 588.0310. $\mathbf{T_d}$: 314 °C. **IR** (cm^{-1}) 3337, 3128, 3066, 1702, 1658, 1635, 1595, 1577, 1472, 1430, 1414, 1404, 1366, 1287, 1251, 1170, 1119, 1105, 849, 820, 774, 760, 708, 689. **UV/Vis:** ε_{392} = 7262 $M^{-1}\cdot cm^{-1}$.

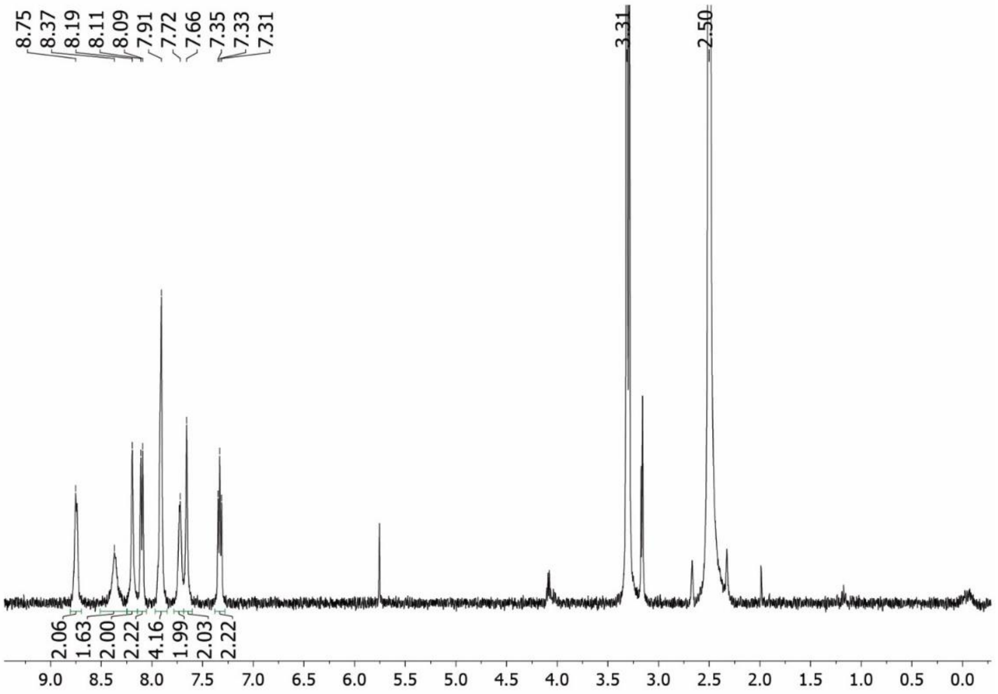

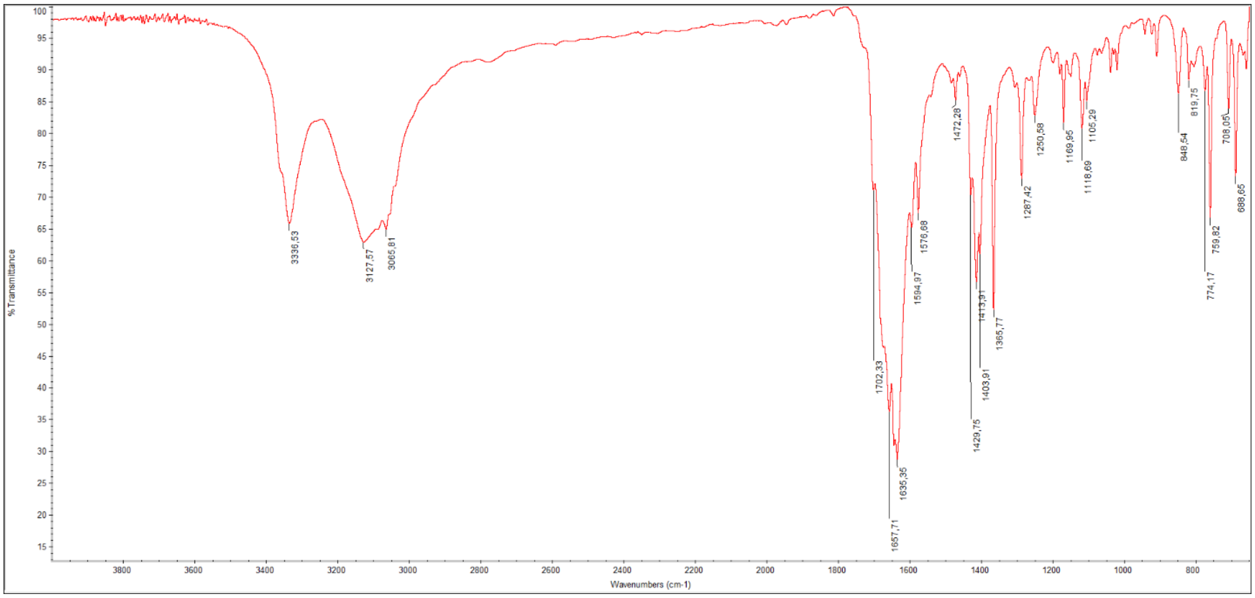

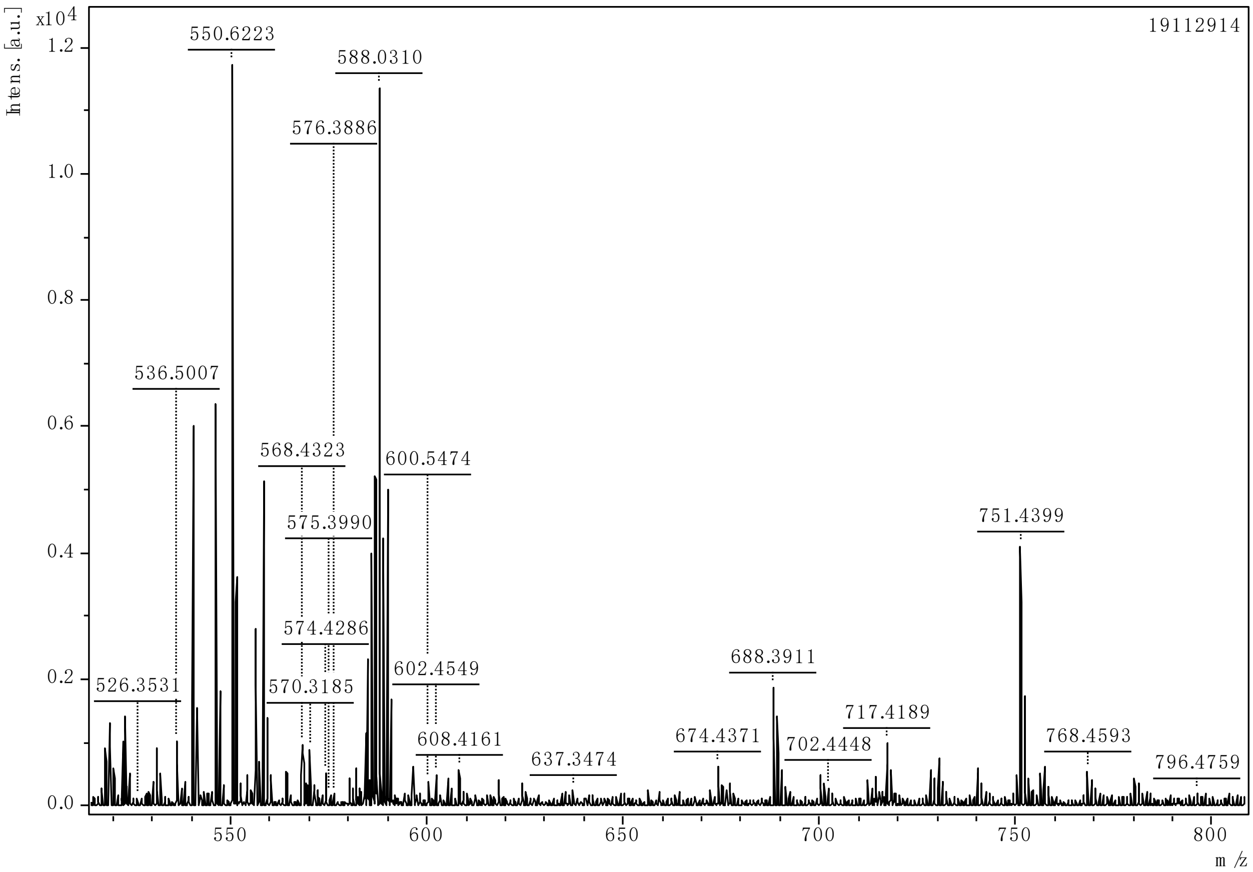

19112914
Intens. [a.u.]
x10^4
550.6223
588.0310
576.3886
536.5007
568.4323
600.5474
575.3990
574.4286
526.3531
570.3185
602.4549
608.4161
637.3474
674.4371
688.3911
702.4448
717.4189
751.4399
768.4593
796.4759
m/z

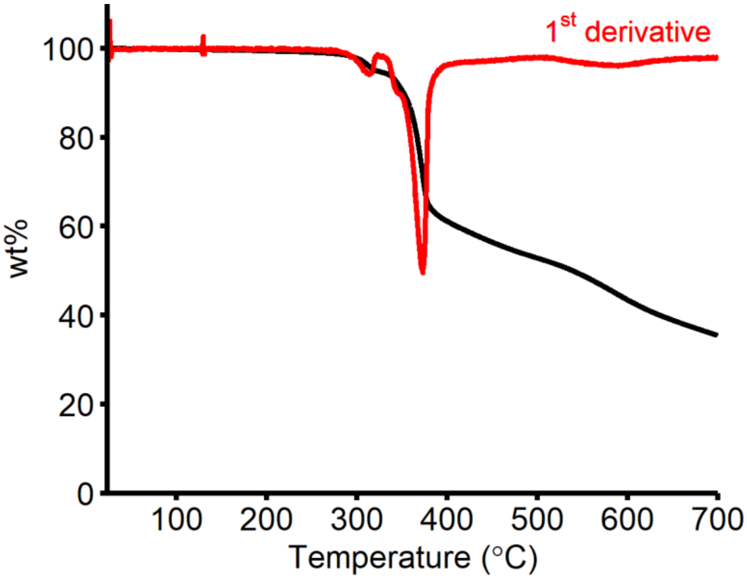

1st derivative
wt%
Temperature (°C)

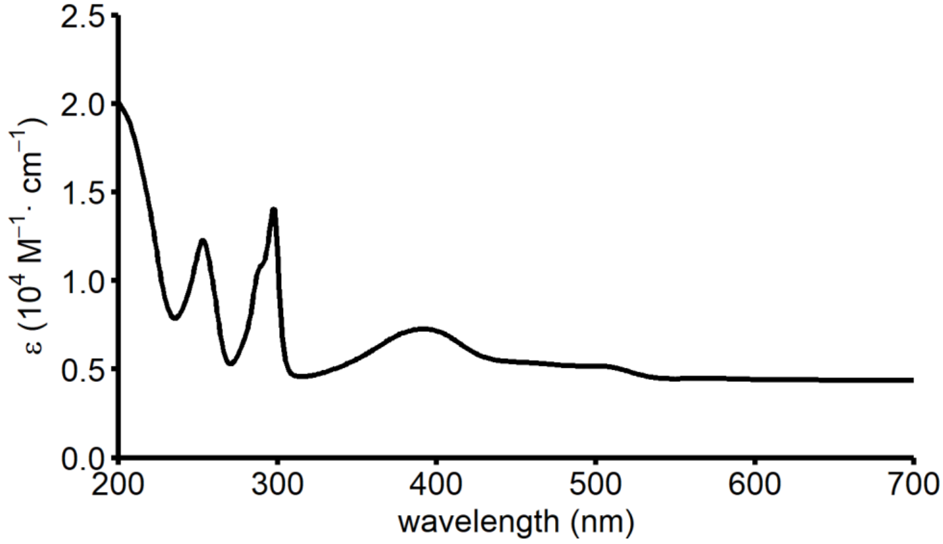

2.5
2.0
1.5
1.0
0.5
0.0
ε (10^4 M^−1· cm^−1)
200
300
400
500
600
700
wavelength (nm)

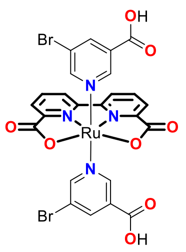

2-*m*CO_2H,*m*Br: Pyridine ligand = 5-bromonicotinic acid (2.5 eq., 0.25 mmol, 51 mg). After 20h reaction time the product was filtered off and washed with MeOH, EtOAc and Et_2O to obtain the product as a red-brown solid (26.4 mg, 35 µmol, 35%).**^{1}H NMR** (400 MHz, DMSO) δ 14.00 (s, 2H), 8.95 – 8.71 (m, 2H), 8.29 (s, 2H), 8.14 (s, 2H), 8.02 – 7.95 (m, 6H). **HRMS:** (TOF-MS ES, m/z) calc. for $C_{24}H_{13}Br_2N_4O_8Ru$ $[M\text{-}H]^-$: 746.8138, found: 746.8135. **T_d**: 265 °C. **IR** (cm^{-1}) 3369, 3111, 3060, 3032, 2936, 2816, 1719, 1624, 1603, 1581, 1433, 1417, 1373, 1296, 1270, 1188, 1167, 1155, 1033, 1025, 774, 679. **UV/Vis:** ε_{401} = 9957 $M^{-1}\cdot cm^{-1}$.

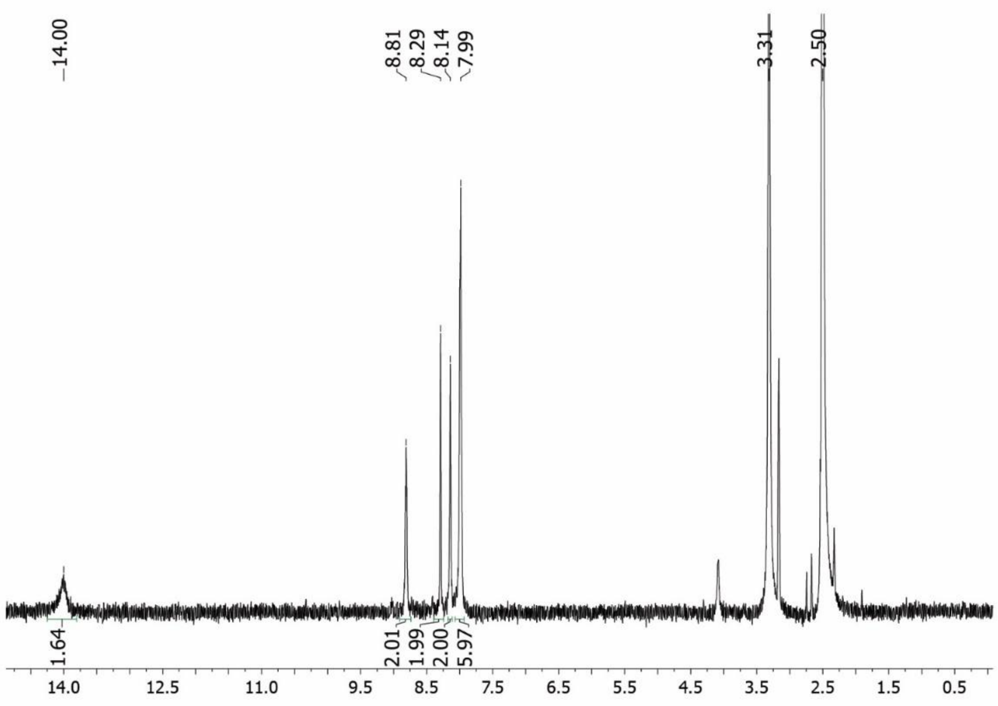

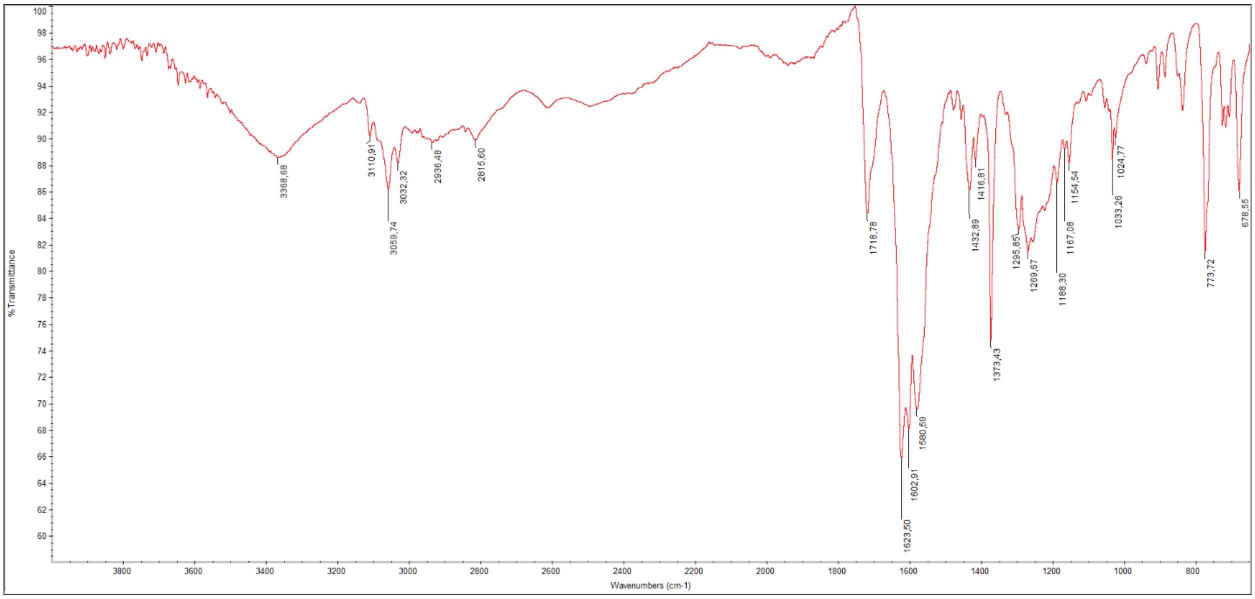

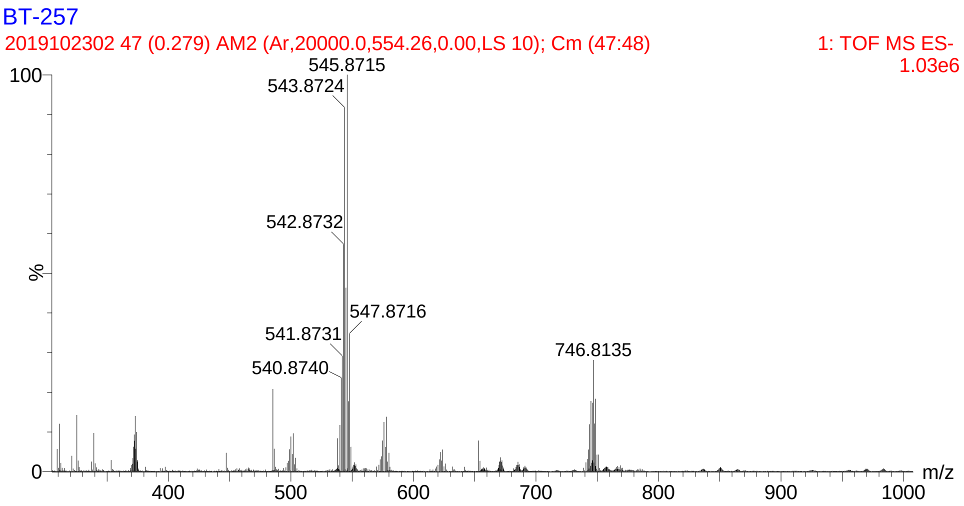
BT-257
2019102302 47 (0.279) AM2 (Ar,20000.0,554.26,0.00,LS 10); Cm (47:48)
1: TOF MS ES-
1.03e6
545.8715
543.8724
542.8732
547.8716
541.8731
540.8740
746.8135
100
%
0
400
500
600
700
800
900
1000
m/z

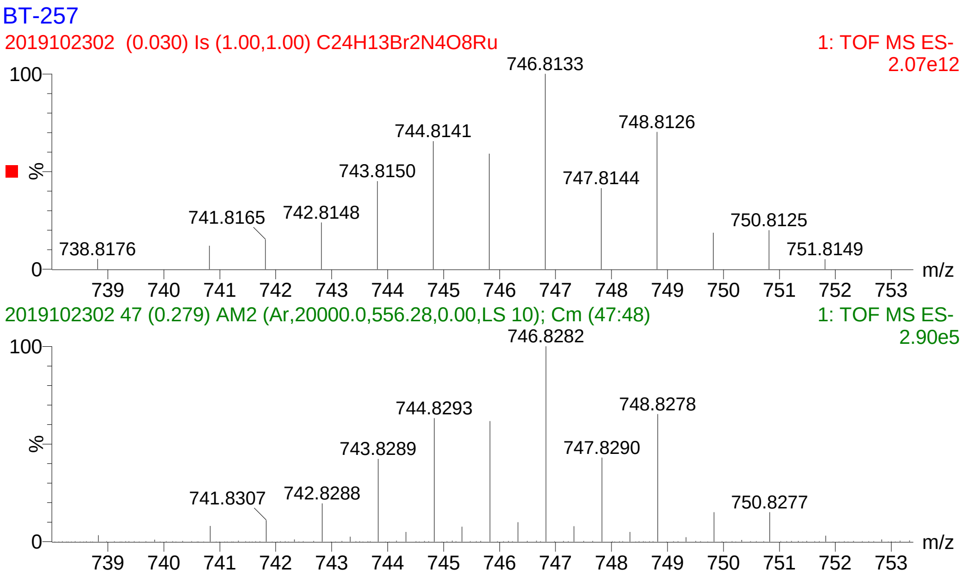
BT-257
2019102302 (0.030) Is (1.00,1.00) C24H13Br2N4O8Ru
1: TOF MS ES-
2.07e12
746.8133
748.8126
744.8141
743.8150
747.8144
741.8165
742.8148
750.8125
738.8176
751.8149
2019102302 47 (0.279) AM2 (Ar,20000.0,556.28,0.00,LS 10); Cm (47:48)
1: TOF MS ES-
2.90e5
746.8282
744.8293
748.8278
743.8289
747.8290
741.8307
742.8288
750.8277
100
%
0
739
740
741
742
743
744
745
746
747
748
749
750
751
752
753
m/z

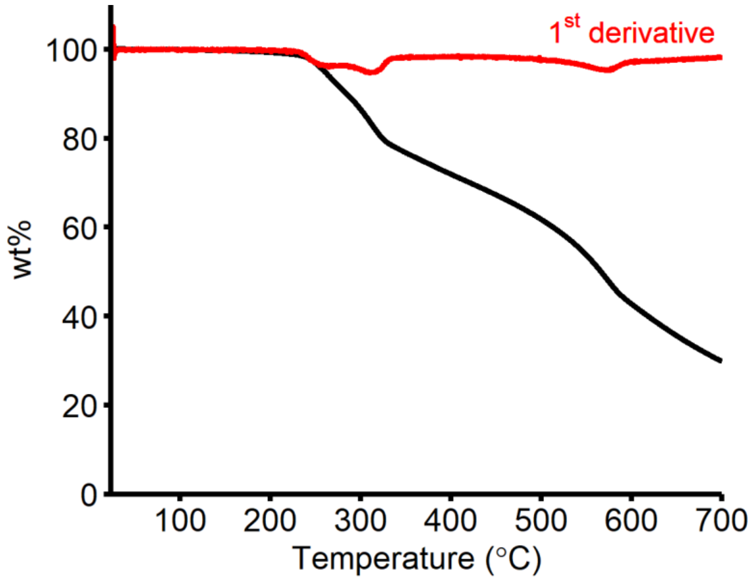
1st derivative
wt%
Temperature (°C)
0
20
40
60
80
100
100
200
300
400
500
600
700

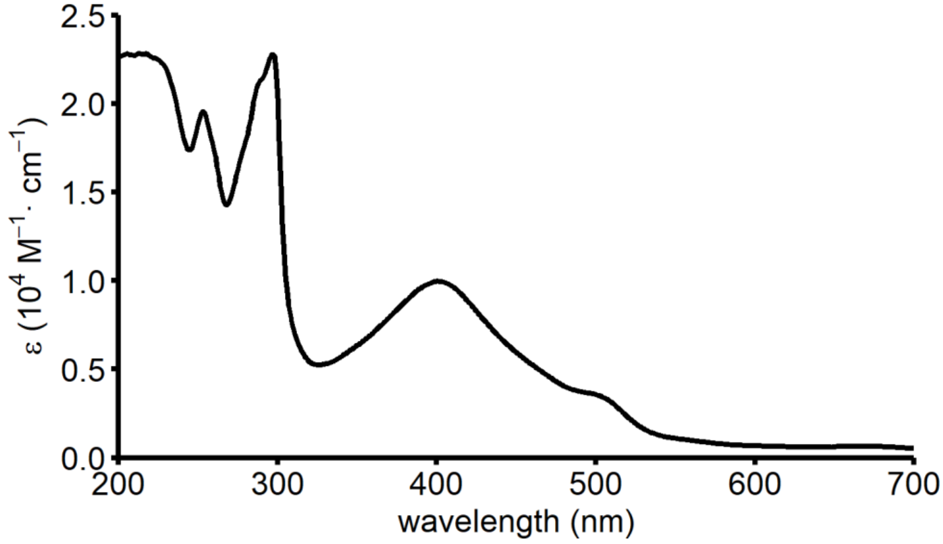
2.5
2.0
1.5
1.0
0.5
0.0
ε (10⁴ M⁻¹· cm⁻¹)
200
300
400
500
600
700
wavelength (nm)

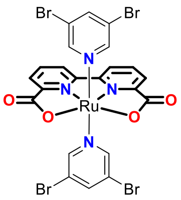

2-*m*Br,*m*Br: Pyridine ligand = 3,5-dibromopyridine (10 eq., 1.0 mmol, 237 mg). After 21h reaction time the product was filtered off and washed with MeOH, EtOAc, CH_2Cl_2, EtOAc and Et_2O to obtain the product as a red-brown solid (32.1 mg, 39 µmol, 39%). **^{1}H NMR** (400 MHz, DMSO) δ 8.77 (t, *J* = 4.3 Hz, 2H), 8.44 (s, 2H), 8.00 (d, *J* = 4.3 Hz, 4H), 7.76 (s, 4H). **HRMS:** (TOF-MS ES, m/z) calc. for $C_{22}H_{12}Br_4N_4O_4RuH$ $[M+H]^+$: 818.6682, found: 818.6680. **T_d**: 272 °C. **IR** (cm^{-1}) 3487, 3087, 3069, 3036, 2973, 1627, 1566, 1536, 1411, 1369, 1301, 1112, 1033, 789, 782, 770, 678. **UV/Vis:** ε_{401} = 12731 $M^{-1}\cdot cm^{-1}$.

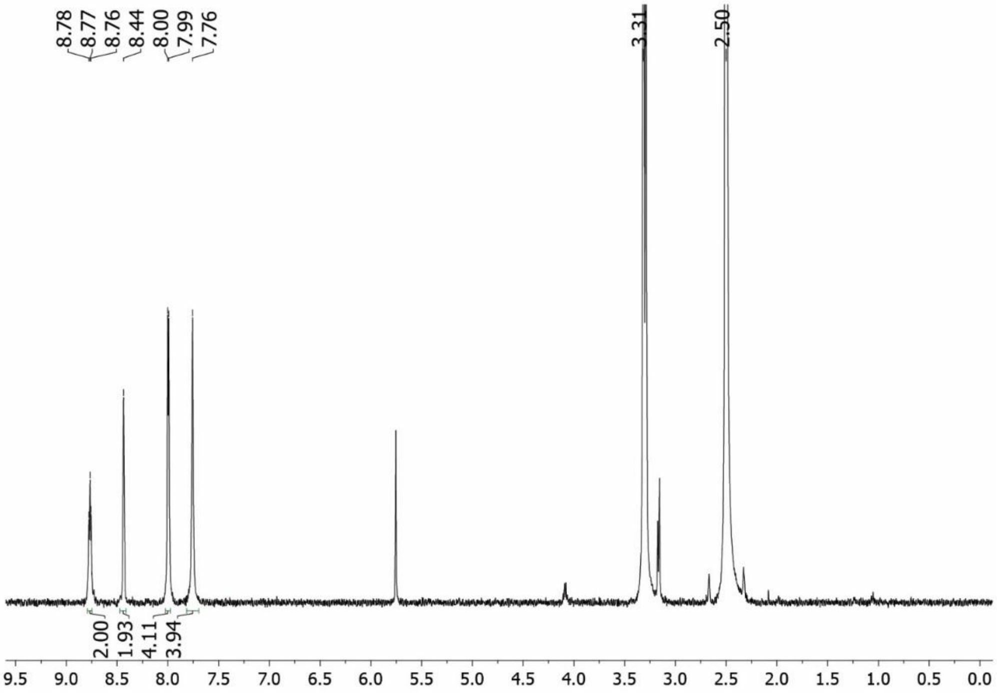

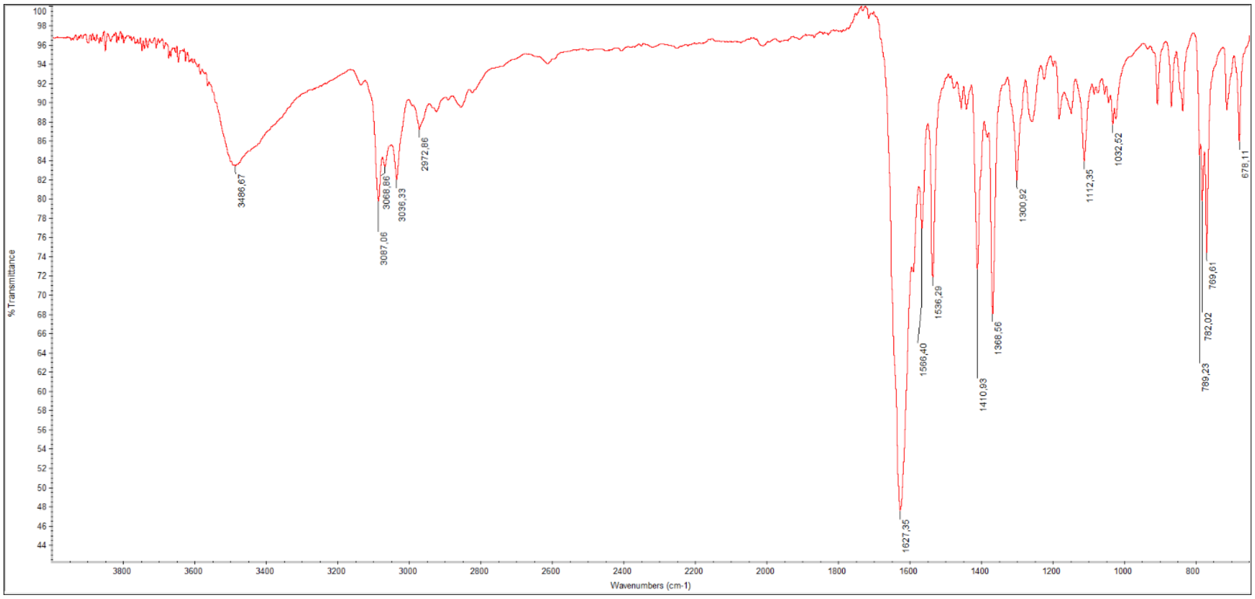

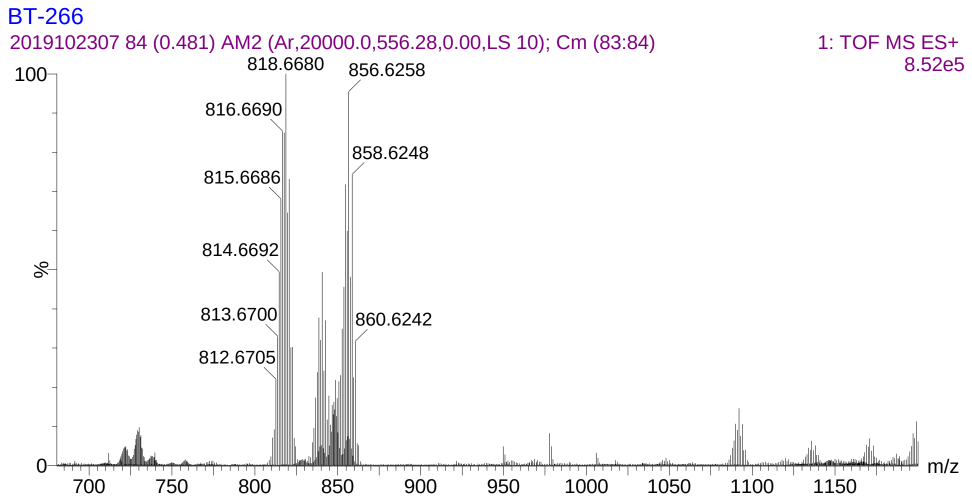
BT-266
2019102307 84 (0.481) AM2 (Ar,20000.0,556.28,0.00,LS 10); Cm (83:84)
1: TOF MS ES+
8.52e5
818.6680
856.6258
816.6690
858.6248
815.6686
814.6692
813.6700
860.6242
812.6705
100
%
0
700
750
800
850
900
950
1000
1050
1100
1150
m/z

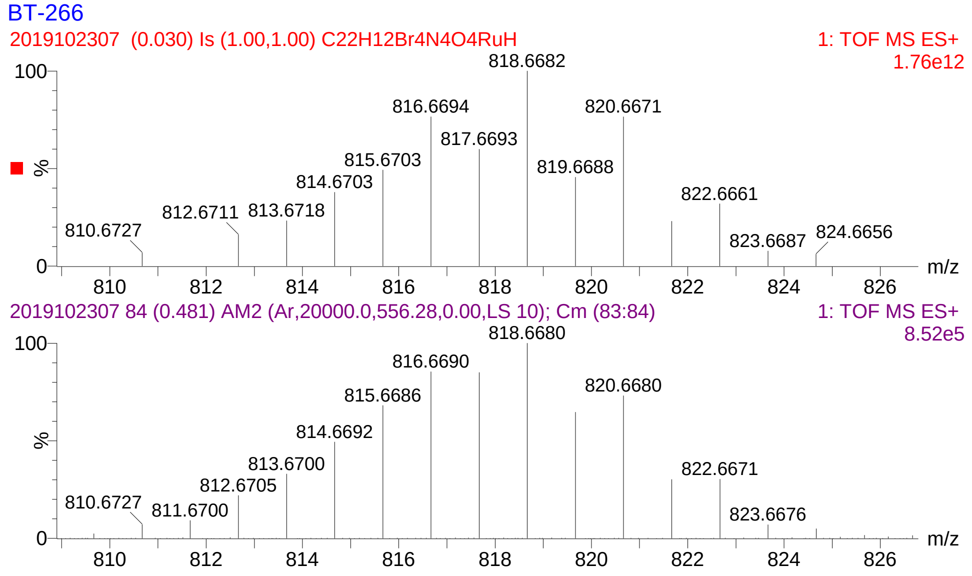
BT-266
2019102307 (0.030) Is (1.00,1.00) C22H12Br4N4O4RuH
1: TOF MS ES+
1.76e12
818.6682
816.6694
820.6671
817.6693
815.6703
819.6688
814.6703
822.6661
812.6711
813.6718
810.6727
824.6656
823.6687
2019102307 84 (0.481) AM2 (Ar,20000.0,556.28,0.00,LS 10); Cm (83:84)
1: TOF MS ES+
8.52e5
818.6680
816.6690
815.6686
820.6680
814.6692
813.6700
822.6671
812.6705
810.6727
811.6700
823.6676
100
%
0
810
812
814
816
818
820
822
824
826
m/z

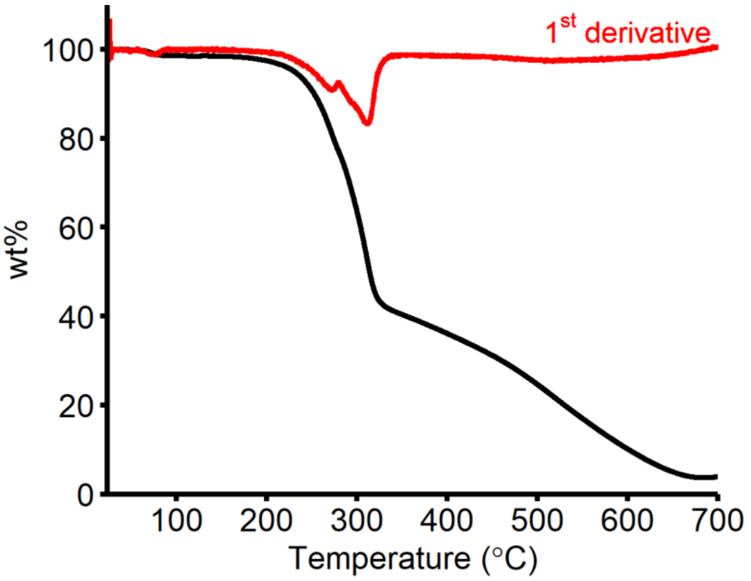
1st derivative
wt%
Temperature (°C)
0
20
40
60
80
100
100
200
300
400
500
600
700

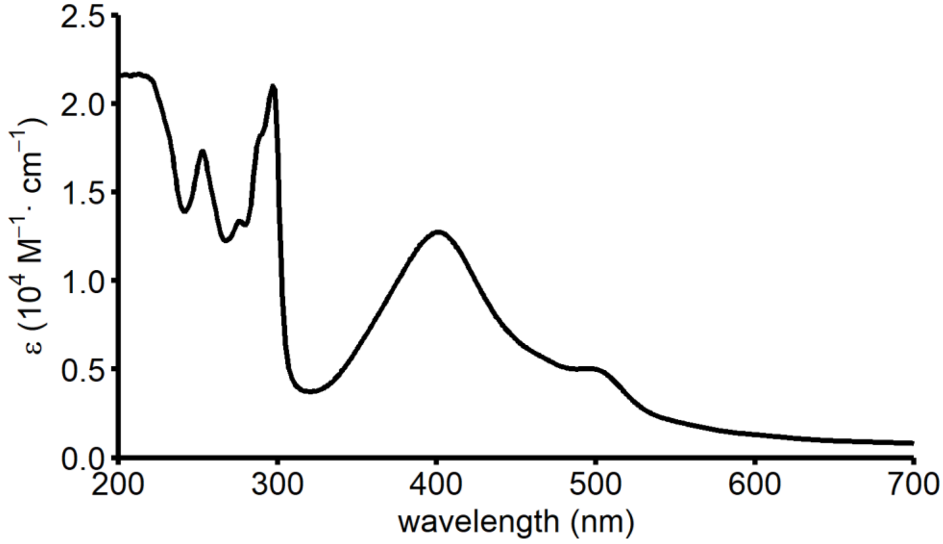

2.5
2.0
1.5
1.0
0.5
0.0
ε (10⁴ M⁻¹· cm⁻¹)
200
300
400
500
600
700
wavelength (nm)

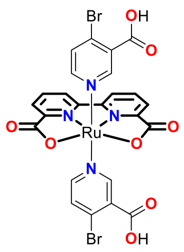

3-*m*CO_2H,*p*Br: Pyridine ligand = 4-bromonicotinic acid (2.5 eq., 0.25 mmol, 51 mg). After 16h reaction time the product was filtered off and washed with MeOH, EtOAc and Et_2O to obtain the product as a red-brown solid (12.6 mg, 17 µmol, 17%). **^{1}H NMR** (400 MHz, DMSO) δ 8.75 (s, 2H), 8.18 (s, 2H), 7.96 (s, 4H), 7.62 (s, 2H), 7.47 (s, 2H). **HRMS:** (TOF-MS ES, m/z) calc. for $C_{24}H_{13}Br_2N_4O_8Ru$ $[M\text{-}H]^-$: 746.8133, found: 746.8131. **T_d**: 267 °C. **IR** (cm^{-1}) 2981, 2938, 1716, 1622, 1584, 1471, 1407, 1367, 1289, 1252, 1175, 1142, 1071, 1033, 761, 703, 670. **UV/Vis:** ε_{410} = 10302 $M^{-1}\cdot cm^{-1}$.

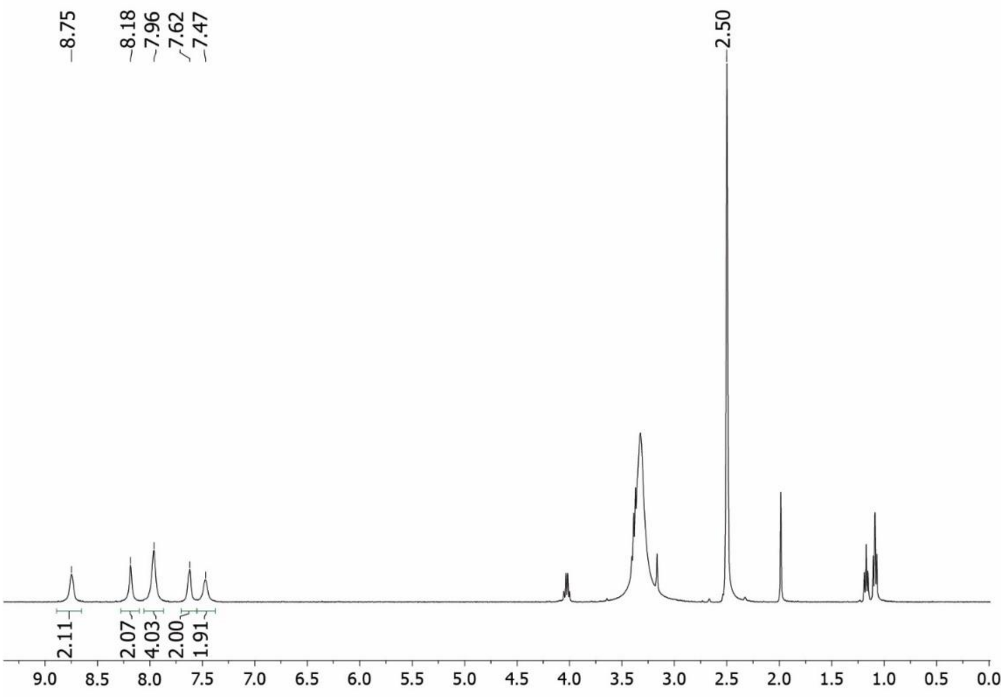

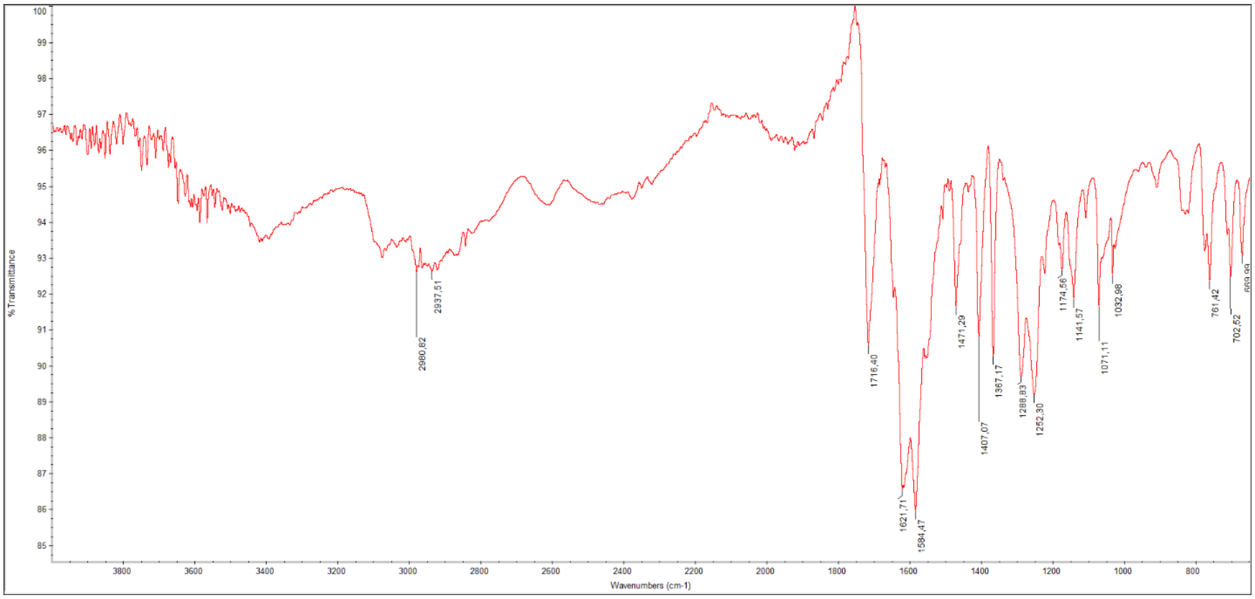

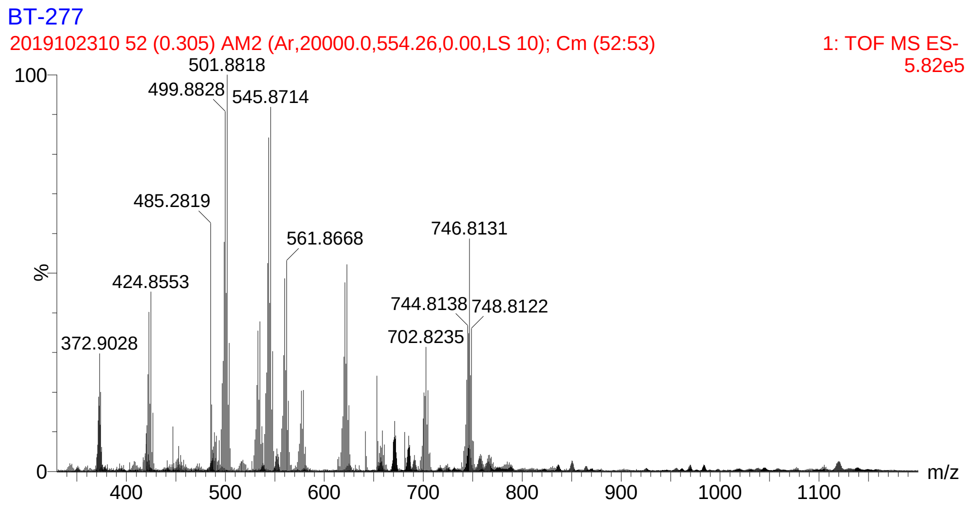
BT-277
2019102310 52 (0.305) AM2 (Ar,20000.0,554.26,0.00,LS 10); Cm (52:53)
1: TOF MS ES-
5.82e5
501.8818
499.8828
545.8714
485.2819
561.8668
746.8131
424.8553
744.8138
748.8122
702.8235
372.9028
100
%
0
m/z
400
500
600
700
800
900
1000
1100

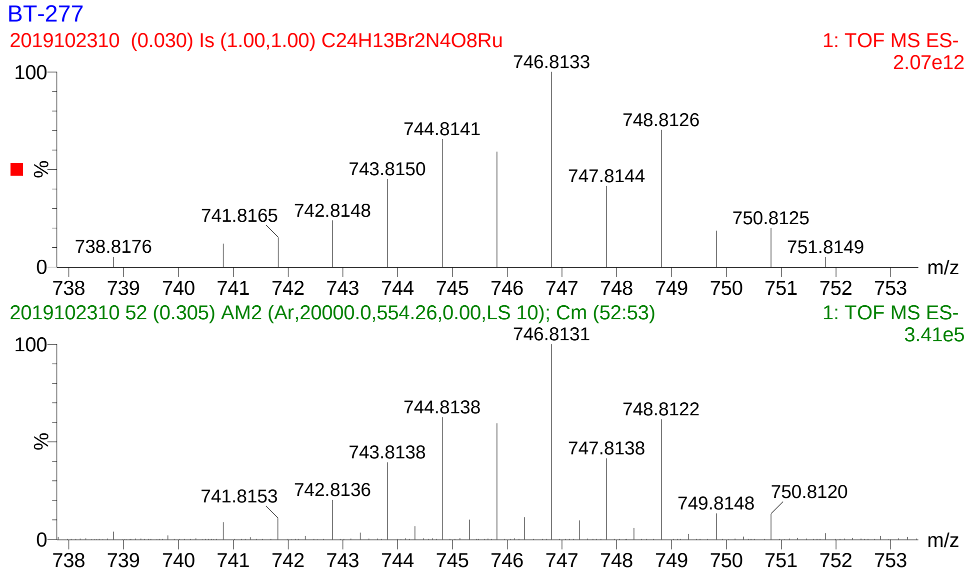
BT-277
2019102310 (0.030) Is (1.00,1.00) C24H13Br2N4O8Ru
1: TOF MS ES-
2.07e12
746.8133
748.8126
744.8141
743.8150
747.8144
741.8165
742.8148
750.8125
738.8176
751.8149
2019102310 52 (0.305) AM2 (Ar,20000.0,554.26,0.00,LS 10); Cm (52:53)
1: TOF MS ES-
3.41e5
746.8131
744.8138
748.8122
743.8138
747.8138
741.8153
742.8136
749.8148
750.8120
100
%
0
m/z
738
739
740
741
742
743
744
745
746
747
748
749
750
751
752
753

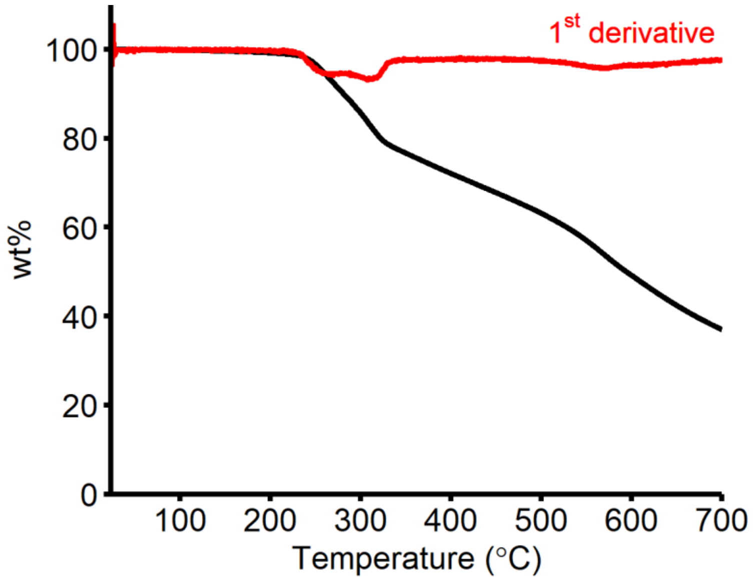
1st derivative
wt%
Temperature (°C)
0
20
40
60
80
100
100
200
300
400
500
600
700

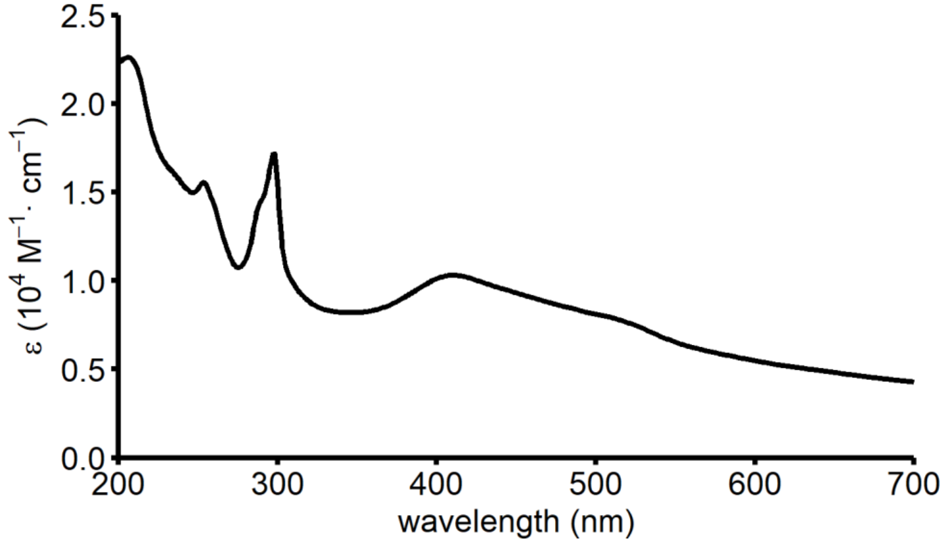

2.5
2.0
1.5
1.0
0.5
0.0
ε (10^4 M^{-1}· cm^{-1})
200
300
400
500
600
700
wavelength (nm)

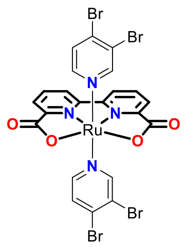

3-*m*Br,*p*Br: Pyridine ligand = 3,4-dibromopyridine (2.5 eq., 0.25 mmol, 59 mg). After 18h reaction time the product was filtered off and washed with MeOH, EtOAc and Et_2O to obtain the product as a red-brown solid (43.0 mg, 53 µmol, 53%). **^{1}H NMR** (400 MHz, DMSO) δ 8.74 (s, 2H), 8.19 (s, 2H), 7.96 (d, *J* = 3.8 Hz, 4H), 7.62 (d, *J* = 5.9 Hz, 2H), 7.21 (d, *J* = 5.3 Hz, 2H). **HRMS:** (TOF-MS ES, m/z) calc. for $C_{22}H_{12}Br_4N_4O_4RuH$ $[M+H]^+$: 818.6682, found: 818.6683. **T_d**: 325 °C. **IR** (cm^{-1}) 3356, 3091, 3058, 3031, 2934, 2815, 1642, 1625, 1568, 1475, 1457, 1443, 1411, 1386, 1367, 1298, 1269, 1178, 1129, 1055, 1043, 1023, 906, 843, 776, 769, 746, 708. **UV/Vis:** ε_{395} = 14667 M^{-1}·cm^{-1}.

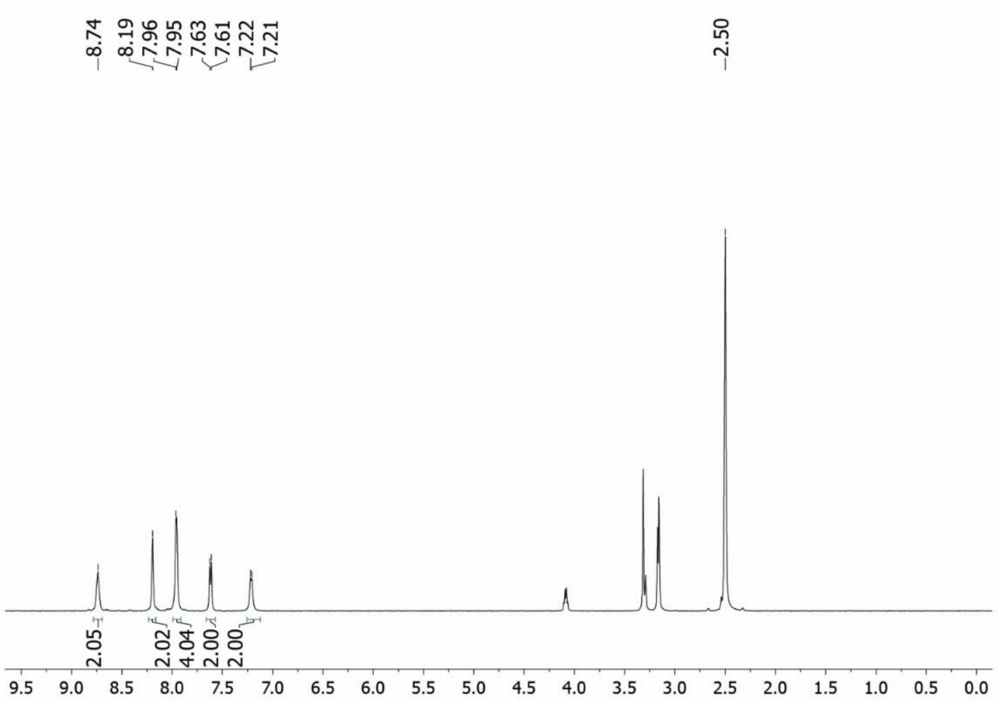

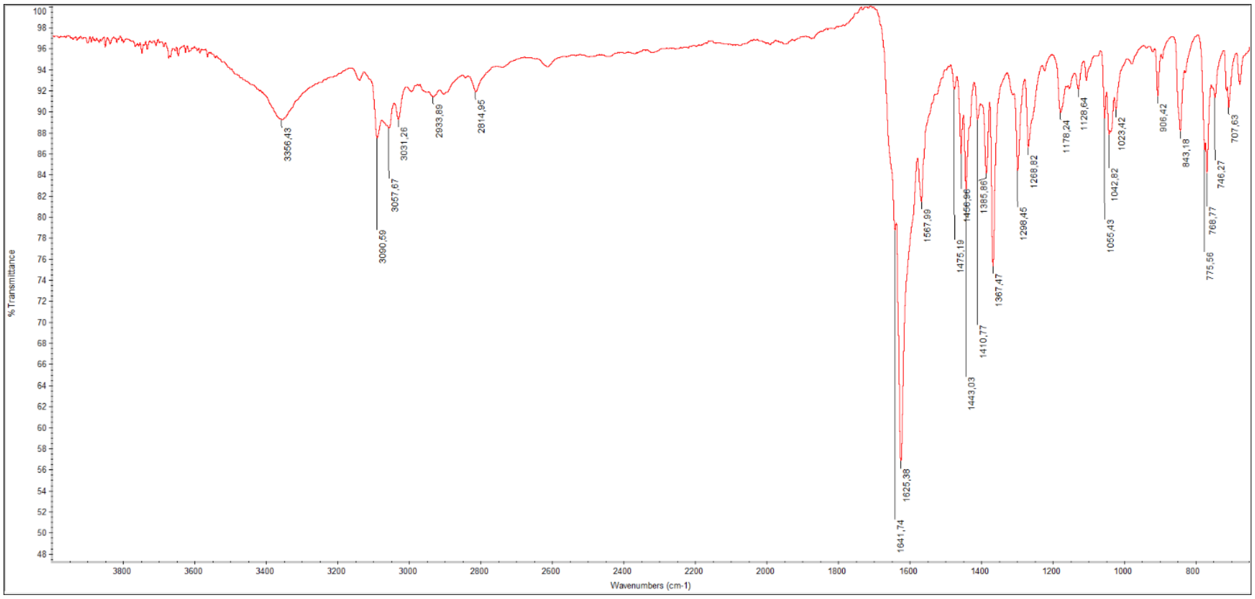

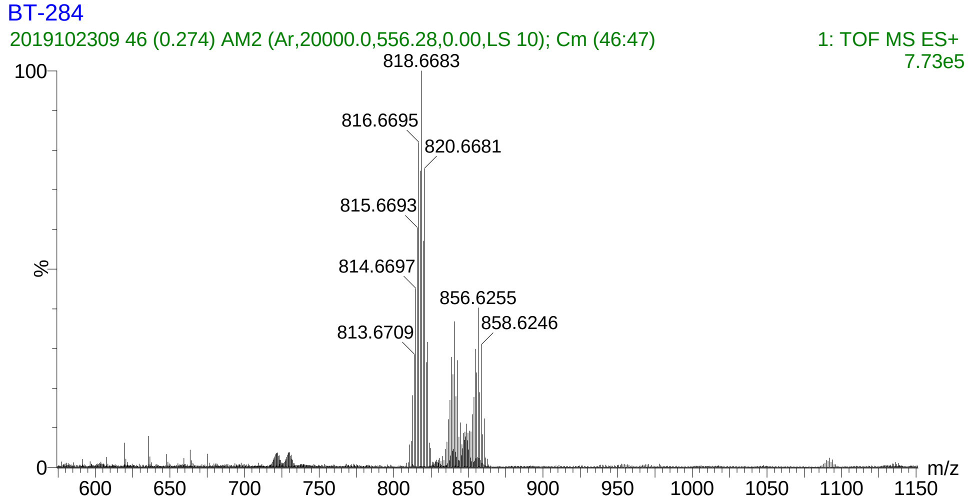
BT-284
2019102309 46 (0.274) AM2 (Ar,20000.0,556.28,0.00,LS 10); Cm (46:47)
1: TOF MS ES+
7.73e5
818.6683
816.6695
820.6681
815.6693
814.6697
856.6255
813.6709
858.6246
%
m/z

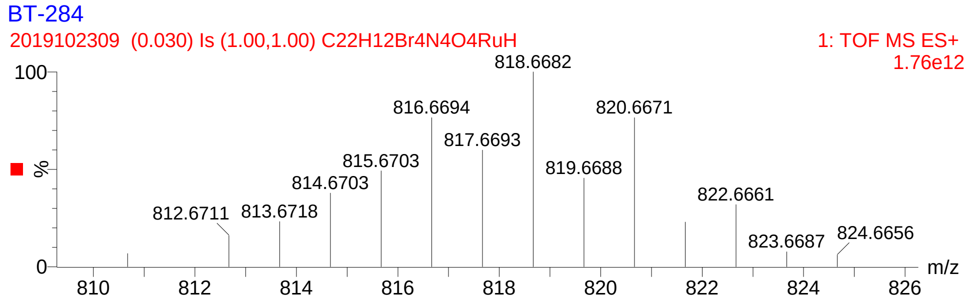
BT-284
2019102309 (0.030) Is (1.00,1.00) C22H12Br4N4O4RuH
1: TOF MS ES+
1.76e12
818.6682
816.6694
820.6671
817.6693
815.6703
819.6688
814.6703
822.6661
812.6711
813.6718
823.6687
824.6656
%
m/z

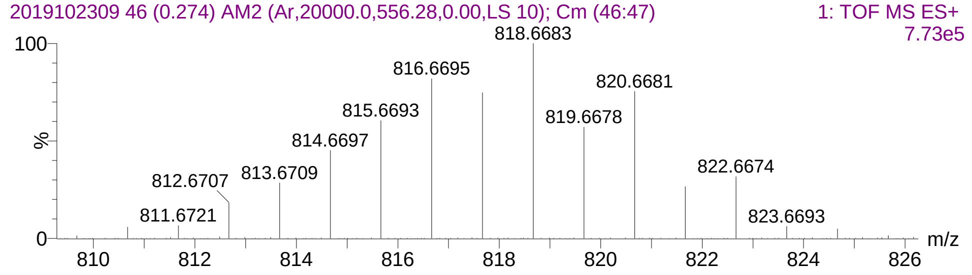
2019102309 46 (0.274) AM2 (Ar,20000.0,556.28,0.00,LS 10); Cm (46:47)
1: TOF MS ES+
7.73e5
818.6683
816.6695
820.6681
815.6693
819.6678
814.6697
813.6709
812.6707
822.6674
811.6721
823.6693
%
m/z

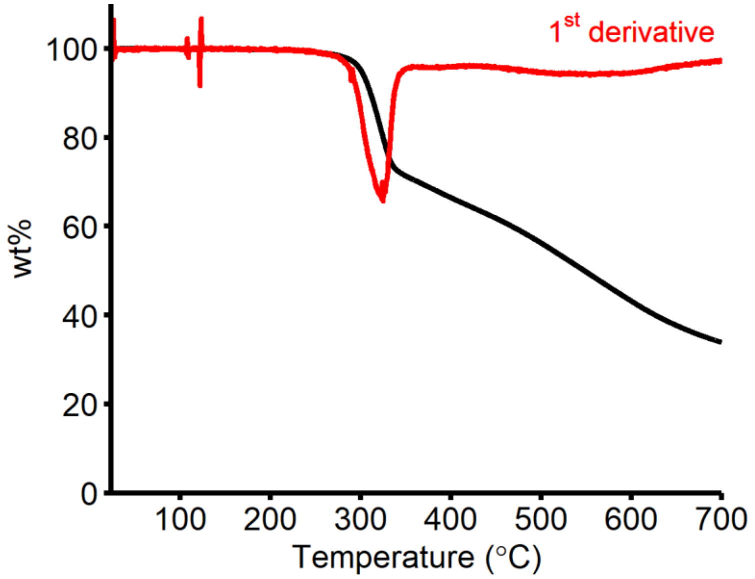
1st derivative
wt%
Temperature (°C)

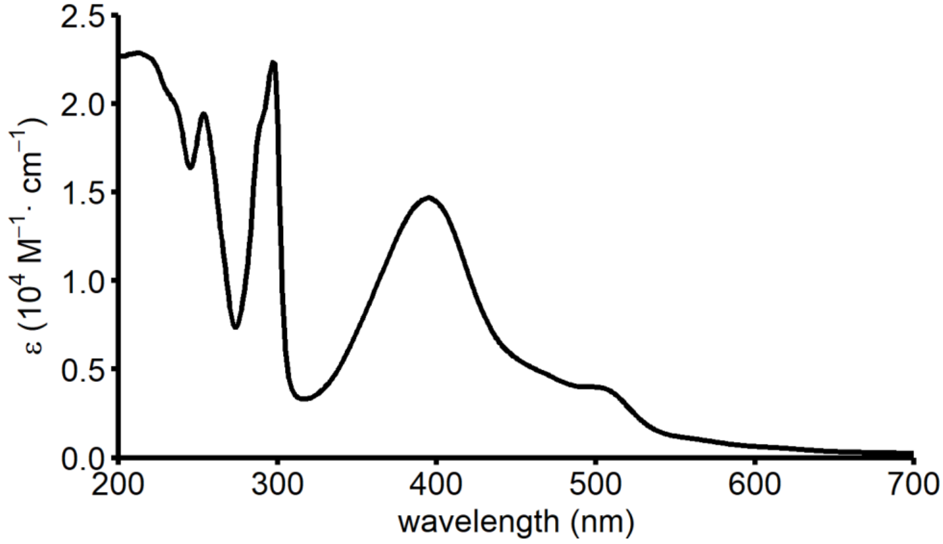

2.5
2.0
1.5
1.0
0.5
0.0
ε (10⁴ M⁻¹· cm⁻¹)
200
300
400
500
600
700
wavelength (nm)

Electrochemical Experiments

General Conditions

The electrochemical experiments were conducted with an Ivium Technologies Vertex Potentiostat (2A) and all data was analyzed using the accompanying software (IviumSoft). For cyclic voltammetry (CV) and differential pulse voltammetry (DPV) measurements, a glassy carbon disk electrode (3mm diameter) was used as the working electrode, a platinum wire was used as the counter electrode, and an Ag/AgCl (satd., 199 mV *vs* NHE) was used as the reference electrode. All potentials reported herein were referenced to NHE. Unless stated otherwise, for both CV and DPV a solution of the catalyst (500 µM) in pH 1 HNO_3 solution containing 20% TFE and a scan rate of 0.1 V/s were used. The last cyclic voltammogram after 5 cycles are provided. For DPV the pulse time was set to 20 ms and the pulse amplitude to 50 mV, with a measurement every 5 mV.

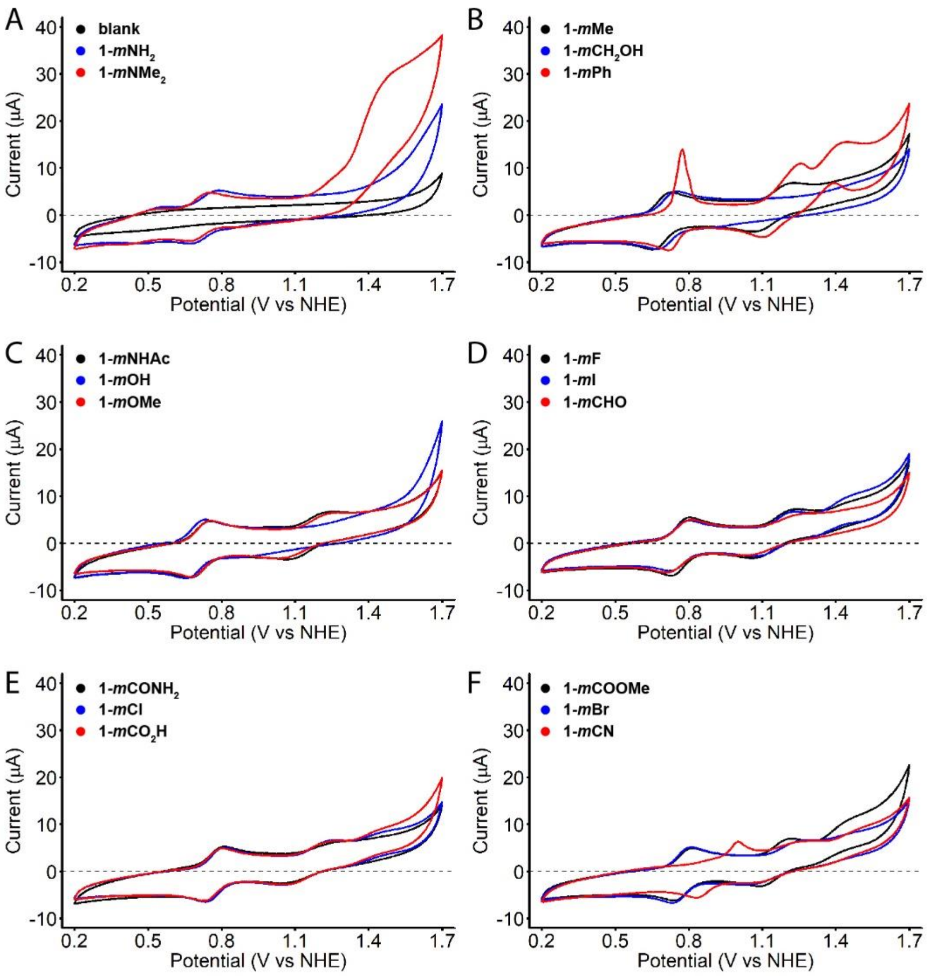

Figure S1. Cyclic voltammograms for a blank solution (20% TFE in pH 1 HNO_3) and the seventeen **1-*m*R** catalysts used in this study.

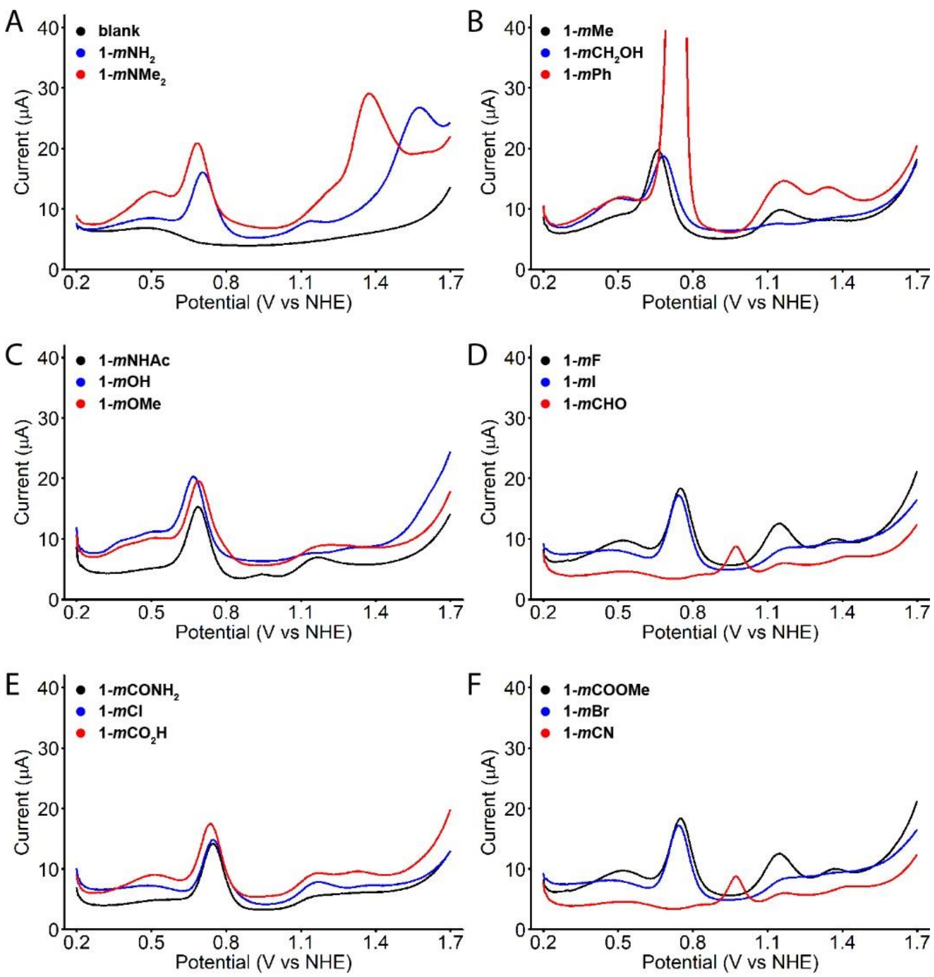

Figure S2. Differential pulse voltammograms for a blank solution (20% TFE in pH 1 HNO_3) and the seventeen **1-*m*R** catalysts used in this study. The peak current observed for **1-*m*Ph** in Figure S2B is 136 μA. The electrochemical data is summarized in table S1.

Table S1. Hammett parameters for the substituents on **1-*m*R** and redox potential for discernible oxidation peaks extracted from DPV and reported in mV *vs* NHE.

Catalyst	σ_m	Ru^{III}/Ru^{II}	Ru^{IV}/Ru^{III}	Ru^{V}/Ru^{IV}
**1-*m*NH₂**	-0.16[a]	705	1125	-
**1-*m*NMe₂**	-0.15[a]	685	1235	1370
**1-*m*Me**	-0.07	660	1150	1350
**1-*m*CH₂OH**	0	680	1120	1340
**1-*m*Ph**	0.06	745	1160	1335
**1-*m*NHAc**	0.07	690	1160	-
**1-*m*OH**	0.12	670	1130	1285
**1-*m*OMe**	0.12	690	1185	-
**1-*m*F**	0.34	740	1145	1370
**1-*m*I**	0.35	735	1150	1345
**1-*m*CHO**	0.35	745	1155	1325
**1-*m*CONH₂**	0.35[b]	750	1160	-
**1-*m*Cl**	0.37	750	1160	1360
**1-*m*CO₂H**	0.37	740	1155	1300
**1-*m* CO₂Me**	0.37	750	1145	1365
**1-*m*Br**	0.39	745	1165	1345
**1-*m*CN**	0.56	975	1160	1410

[a]The σ_m-value displayed is for the non-protonated species, the σ_m-value of the ammonium groups would be ~0.86. [b]The displayed σ_m-value is for the –CONHMe substituted aryl group, most closely related to the substituent used for **1-*m*CONH₂** for which no Hammett data was available.

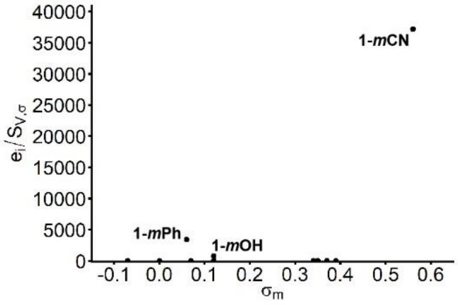

Figure S3. Outlier analysis by plotting the normalized residuals ($e_i/S_{V,\sigma}$) of the Ru^{III}/Ru^{II} redox couple plotted against the Hammett parameter (σ_m) of the substituent in 1-*m*R. Outliers were removed for all other correlation analyses as well.

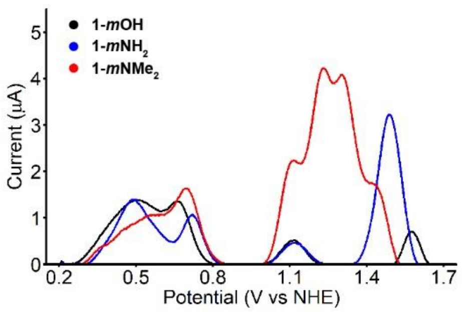

Figure S4. Differential pulse voltammograms for **1-*m*OH**, **1-*m*NH$_2$**, and **1-*m*NMe$_2$** measured at 50 μM catalyst concentration in pH 1 HNO3 containing 10% TFE.

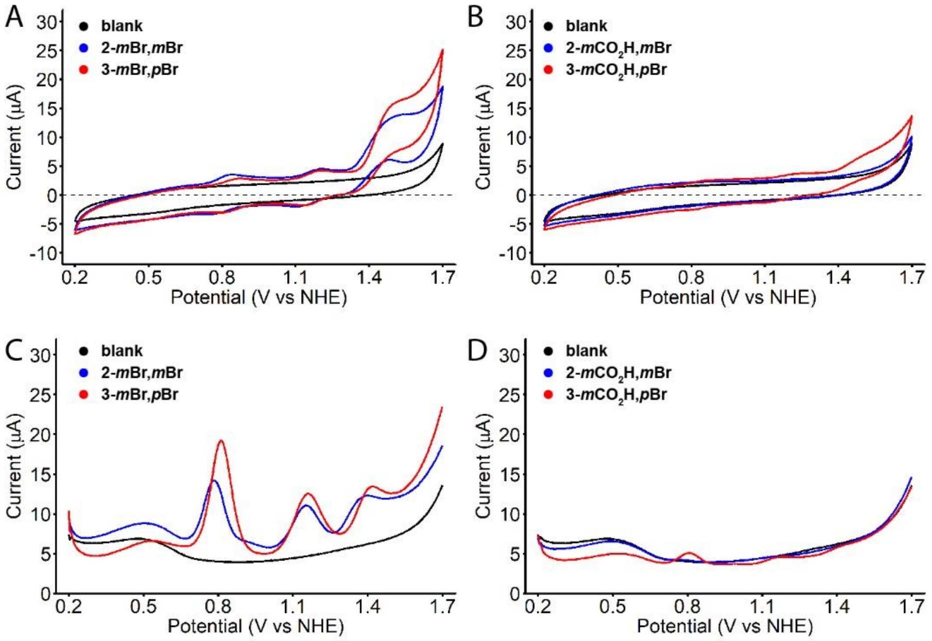

Figure S5. Cyclic voltammograms and differential pulse voltammograms for a blank solution (20% TFE in pH 1 HNO$_3$) and **2-*m*Br,*m*Br**, **3-*m*Br,*p*Br**, **2-*m*CO$_2$H,*m*Br**, **3-*m*CO$_2$H,*p*Br**. Due to low solubility of the carboxylic acid derived catalysts, the electrochemical response was relatively low.

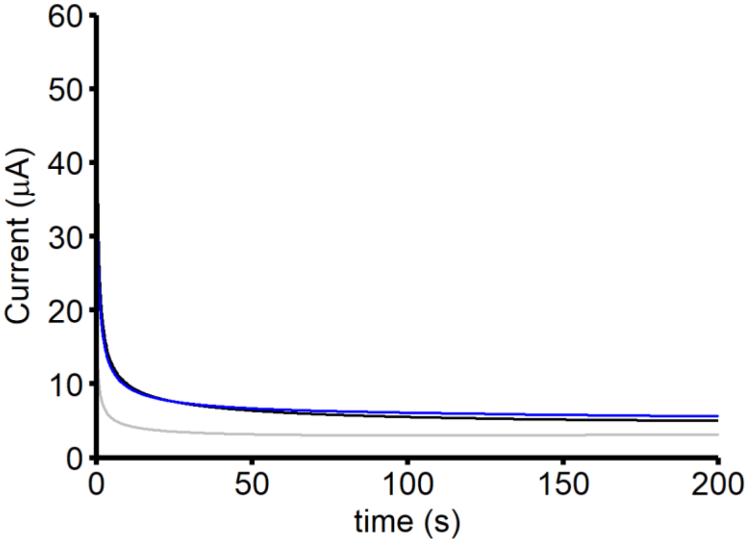

Figure S6. Controlled potential electrolysis at 1.6V using a glassy carbon working electrode in pH 1 triflic acid containing 5% CF_3CH_2OH in the presence of either no added catalyst (gray), **1-*m*Me** (black) or **Ru(bda)(pic)$_2$** (blue).

Pressure Sensor Kinetics

A pressure tube capped with a MIK-P300 Diffused silicon pressure transmitter was charged with 1.8 mL of a solution of cerium(IV) ammonium nitrate (CAN, 0.406M) in pH 1 HNO_3 and vigorously stirred. The 10X catalyst solutions were prepared by dissolving the desired catalyst in 1:1 CF_3CH_2OH and pH 1 HNO_3, for which part of the pH 1 HNO_3 was replaced with the CAN solution to achieve a theoretical 10 turnovers prior to oxygen evolution. This step was performed to increase solubility of the catalyst solution to be injected, to increase reproducibility and accuracy of the measured results. Through a GC-septum inlet 200 μL of the catalyst solutions was quickly injected (final concentration of Ce^{IV} = 0.365M). The pressure transmitter response was converted to TONs based on the maximum theoretically achievable turnovers. Full consumption of the Ce^{IV} could clearly be observed by eye, and full conversion into the desired product O_2 was checked and confirmed by injection of 400 μL of the headspace into a gas chromatograph (GC-2014, Shimadzu). The total amount of formed O_2 was determined by subtraction of the quantity of oxygen in air. For **2-*m*Br,*m*Br**, **3-*m*Br,*p*Br**, **2-*m*CO$_2$H,*m*Br**, **3-*m*CO$_2$H,*p*Br** the chemical oxygen evolution experiments are performed in triplicate and the TOFs are reported with standard deviation.

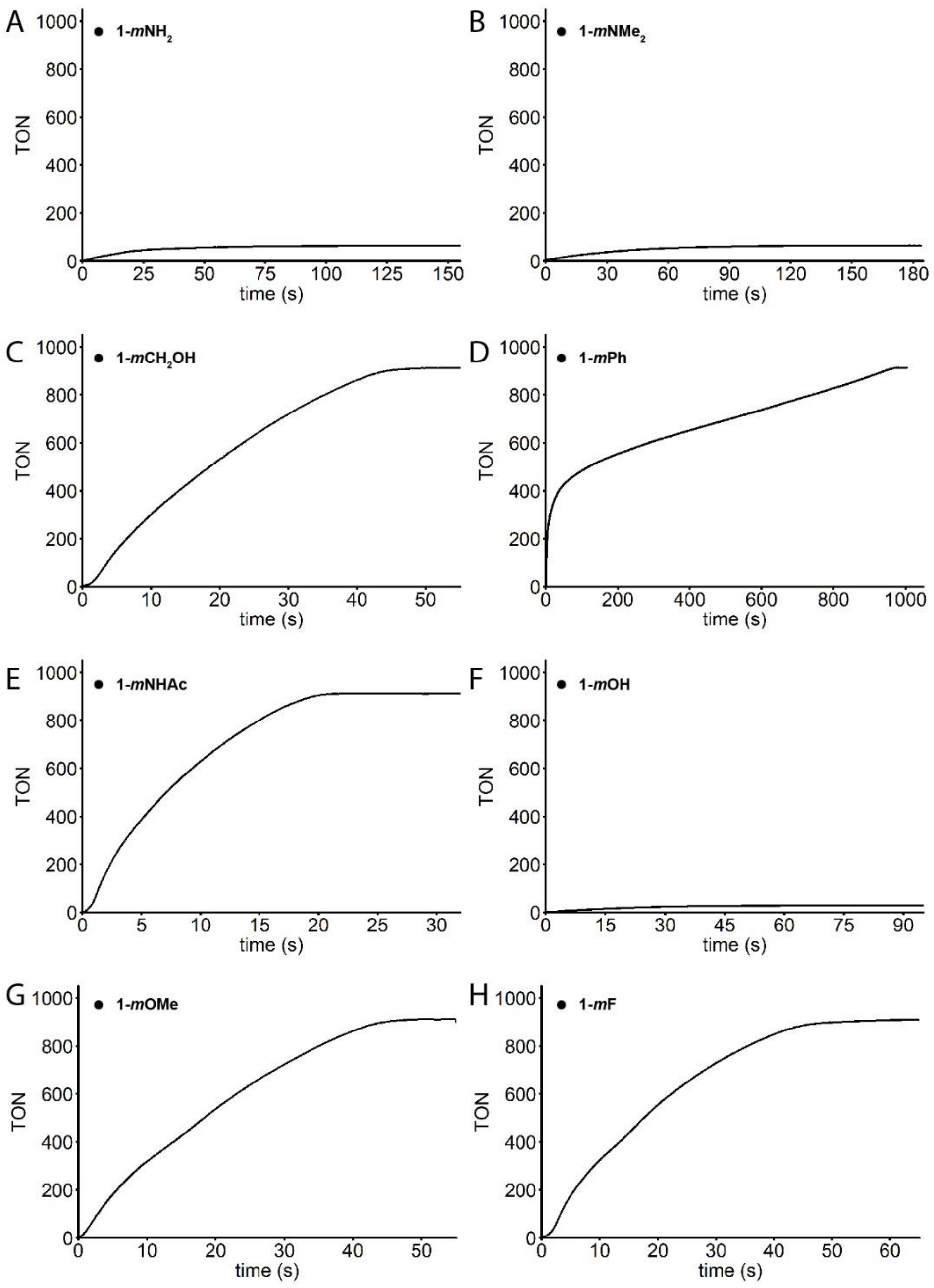

Figure S7. Catalytic performances of part of the **1-*m*R** catalyst series. [Ce^{IV}] = 0.365 M; [**1-*m*R**] = 100 µM, in pH 1 HNO_3 in H_2O containing 5% CF_3CH_2OH. TOF_{init} was calculated by retrieving the maximum from a linear regression analysis connecting five measurement points. TON_{max} is limited by the oxidant to 912.5. **1-*m*NH₂**, **1-*m*OH** and **1-*m*NMe₂** did not achieve full conversion of Ce^{IV} under these conditions.

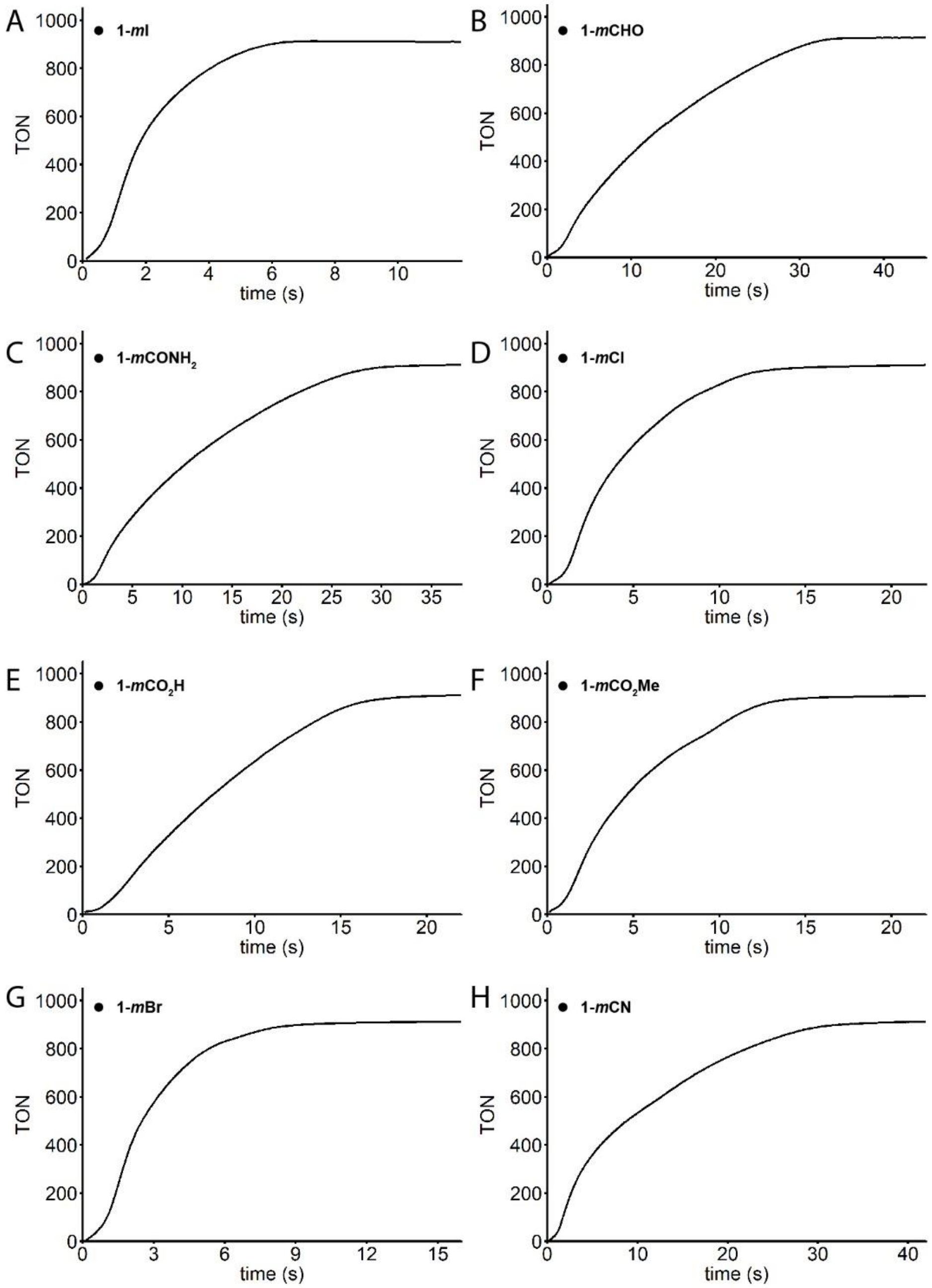

Figure S8. Catalytic performances of part of the **1-*m*R** catalyst series. [Ce^{IV}] = 0.365 M; [**1-*m*R**] = 100 µM, in pH 1 HNO_3 in H_2O containing 5% CF_3CH_2OH. TOF_{init} was calculated by retrieving the maximum from a linear regression analysis connecting five measurement points. TON_{max} is limited by the oxidant to 912.5.

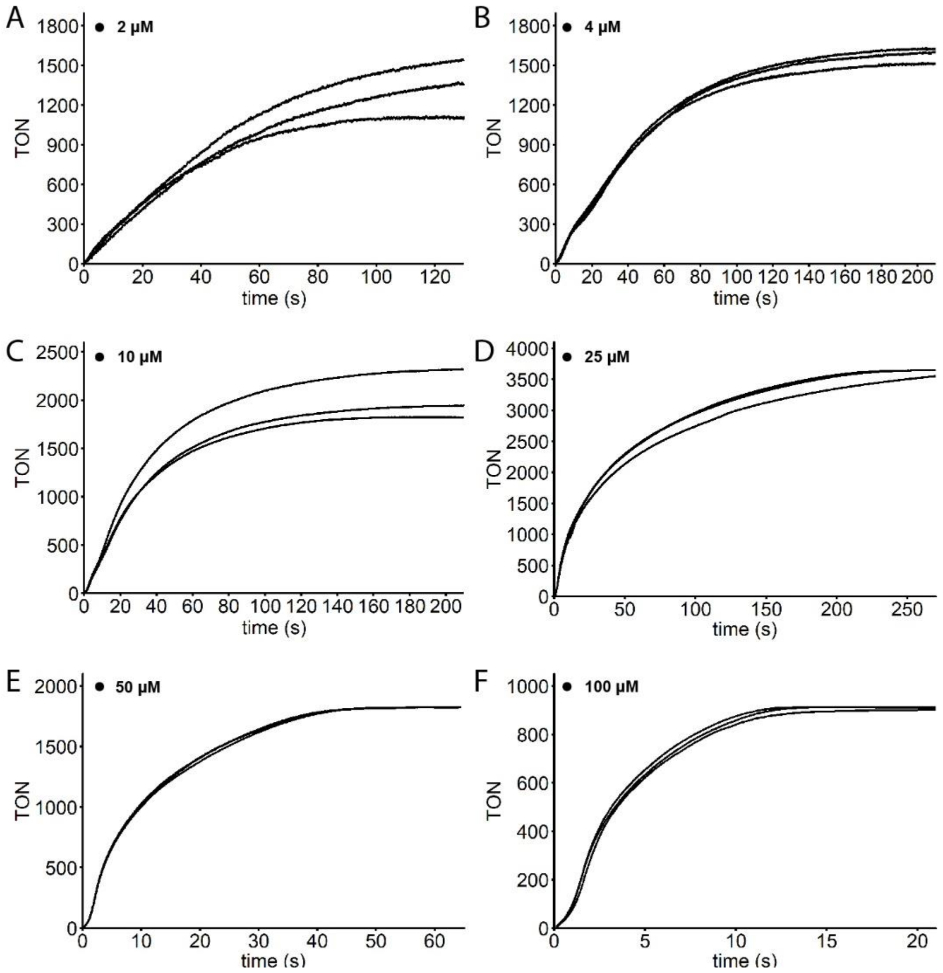

Figure S9. Catalytic performances of **2-*m*Br,*m*Br** catalyst at varying catalyst concentrations. [Ce^{IV}] = 0.365 M in pH 1 HNO_3 in H_2O containing 5% CF_3CH_2OH. TOF_{init} was calculated by retrieving the maximum from a linear regression analysis connecting a minimum of five measurement points. For [**2-*m*Br,*m*Br**] of 2, 4 and 10 µM no full consumption of Ce^{IV} could be achieved.

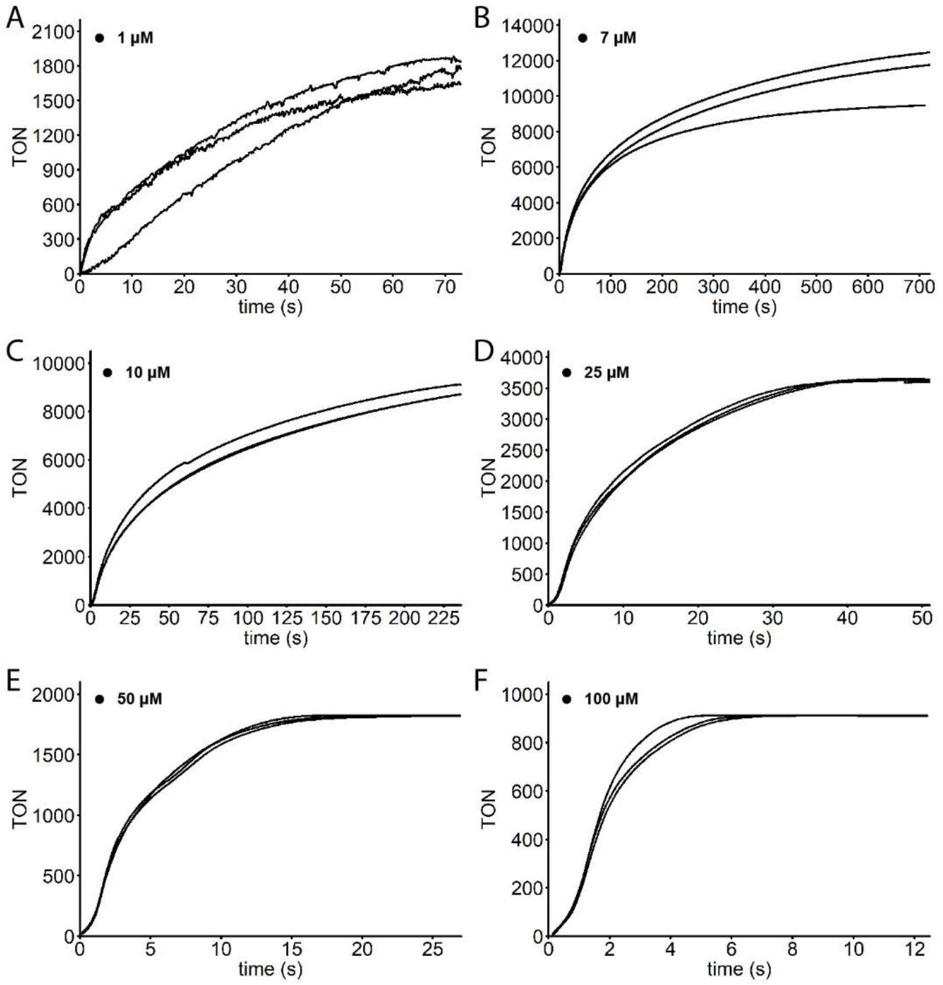

Figure S10. Catalytic performances of **3-*m*Br,*p*Br** catalyst at varying catalyst concentrations. [Ce^{IV}] = 0.365 M in pH 1 HNO_3 in H_2O containing 5% CF_3CH_2OH. TOF_{init} was calculated by retrieving the maximum from a linear regression analysis connecting a minimum of three measurement points. For [**3-*m*Br,*p*Br**] of 1 and 7 µM no full consumption of Ce^{IV} could be achieved. For the lowest concentration (1 µM) the TOF was extracted from the 15-25 second time interval due to the relatively low signal to noise ratio.

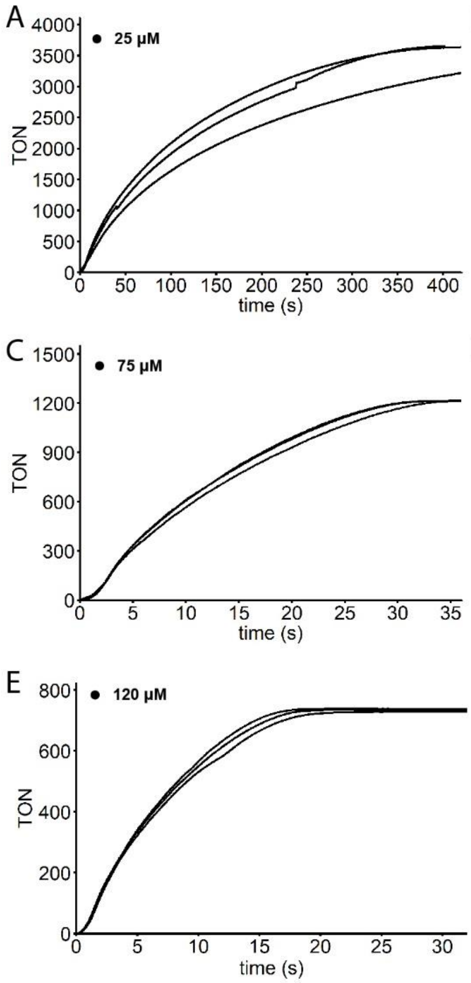

Figure S11. Catalytic performances of **2-*m*CO$_2$H,*m*Br** catalyst at varying catalyst concentrations. [Ce^{IV}] = 0.365 M in pH 1 HNO_3 in H_2O containing 5% CF_3CH_2OH. TOF$_{init}$ was calculated by retrieving the maximum from a linear regression analysis connecting a minimum of five measurement points.

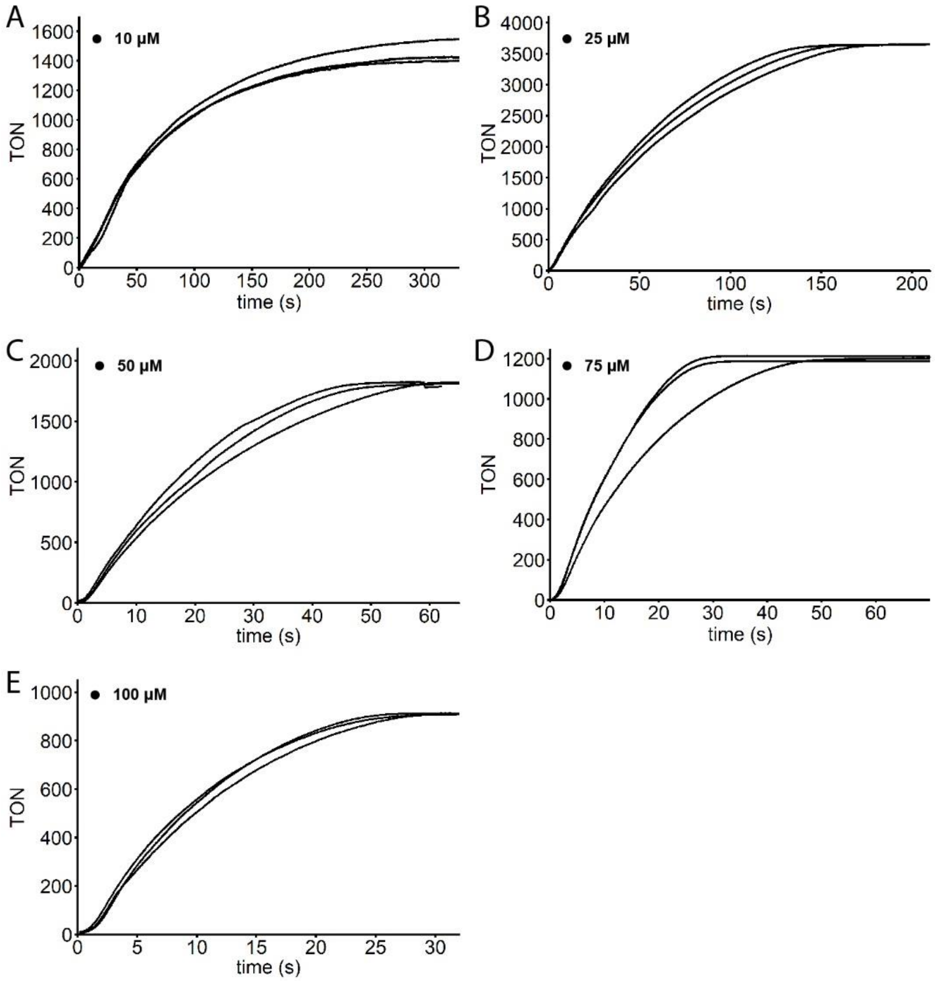

Figure S12. Catalytic performances of **3-*m*CO$_2$H,*p*Br** catalyst at varying catalyst concentrations. [Ce^{IV}] = 0.365 M in pH 1 HNO_3 in H_2O containing 5% CF_3CH_2OH. TOF$_{init}$ was calculated by retrieving the maximum from a linear regression analysis connecting a minimum of five measurement points. For [**3-*m*CO$_2$H,*p*Br**] of 10 µM no full consumption of Ce^{IV} could be achieved.

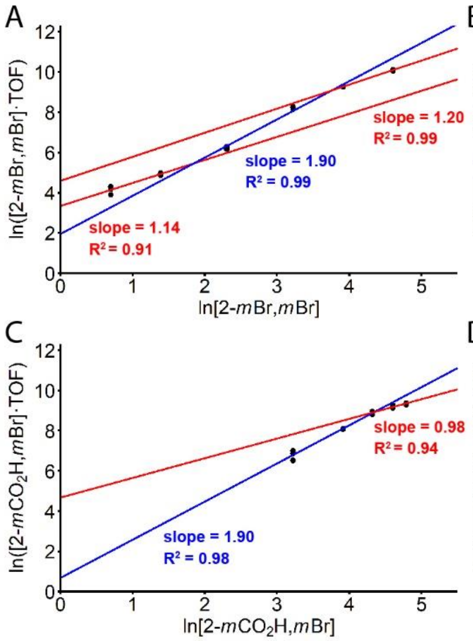

Figure S13. Correlation plots of ln[**Cat**·TOF] *vs* ln[**Cat**] to investigate the order of the disubstituted catalysts in the oxygen evolution reaction at different concentrations. A slope of 2 indicates 2nd order dependence on catalyst concentration, whereas a slope of 1 indicates 1st order dependence on catalyst concentration.

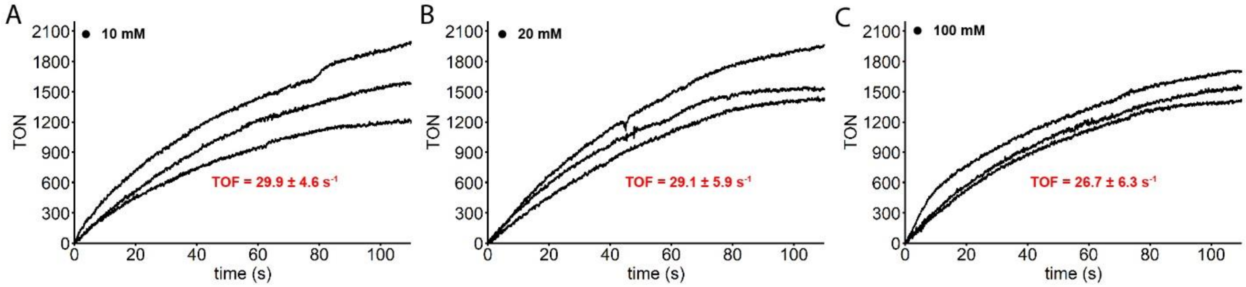

Figure S14. Catalytic performance of 1 μM **3-*m*Br,*p*Br** at varying [Ce^{IV}]. TOFs were extracted from the 5-15 second time interval due to the relatively low signal to noise ratio.

Table S2. Concentrations of catalyst and observed TOFs for the data presented in Figure S13. Datapoints are measured in triplicate and shown as average with accompanying standard deviation.

**Catalyst**	Concentration (μM)	TOF (s^{-1})
**2-*m*Br,*m*Br**	2	31.8 ± 6.2
**2-*m*Br,*m*Br**	4	34.7 ± 2.1
**2-*m*Br,*m*Br**	10	51.7 ± 3.6
**2-*m*Br,*m*Br**	25	152.6 ± 6.4
**2-*m*Br,*m*Br**	50	213.5 ± 3.9
**2-*m*Br,*m*Br**	100	244.6 ± 9.7

**Catalyst**	Concentration (μM)	TOF (s^{-1})
**3-*m*Br,*p*Br**	1	31.4 ± 2.5
**3-*m*Br,*p*Br**	7	206.1 ± 5.6
**3-*m*Br,*p*Br**	10	288.2 ± 21.9
**3-*m*Br,*p*Br**	25	429.1 ± 19.0
**3-*m*Br,*p*Br**	50	458.7 ± 6.2
**3-*m*Br,*p*Br**	100	460.1 ± 32.4

**Catalyst**	Concentration (μM)	TOF (s^{-1})
**2-*m*CO_2H,*m*Br**	25	36.7 ± 8.5
**2-*m*CO_2H,*m*Br**	50	65.2 ± 0.7
**2-*m*CO_2H,*m*Br**	75	96.9 ± 6.0
**2-*m*CO_2H,*m*Br**	100	99.2 ± 6.4
**2-*m*CO_2H,*m*Br**	120	95.3 ± 4.0

**Catalyst**	Concentration (μM)	TOF (s^{-1})
**3-*m*CO_2H,*p*Br**	10	18.3 ± 1.4
**3-*m*CO_2H,*p*Br**	25	57.3 ± 3.1
**3-*m*CO_2H,*p*Br**	50	76.2 ± 6.6
**3-*m*CO_2H,*p*Br**	75	75.0 ± 11.2
**3-*m*CO_2H,*p*Br**	100	83.2 ± 1.3

UV-Vis Stability Check

To determine if the difference in reactivity between **Ru(bda)(pic)$_2$** and **1-*m*Me** was a result of a difference in their respective stability, an catalyst recovery experiment was run for both catalysts using their distinctive UV/Vis absorption pattern. A 1 cm cuvette was loaded with either 200 µL of a 1 mM catalyst solution and 1800 µL pH 1 aqueous HNO_3, or 200 µL of a 1 mM catalyst solution and 1800 µL of 0.406M CAN in pH 1 HNO_3. After completion of the oxygen evolution in the 2nd cuvette the catalyst was reduced back to the catalyst precursor by addition of a total of 25 and 250 µL 10 mM ascorbic acid. Excess ascorbic acid was necessary to fully reduce any remaining catalyst back to the Ru^{II} state. The average ratio between the characteristic and stable UV/Vis absorption from 450 to 500 nm was found to be 0.74 (26% decomposition) vs 0.80 (20% decomposition) for **Ru(bda)(pic)$_2$** and **1-*m*Me**, respectively. The observed 6% difference in decomposition of both catalysts during the oxidation reaction rules out that difference in catalyst stability is the major contributor for the 2.5-fold difference observed in catalyst activity.

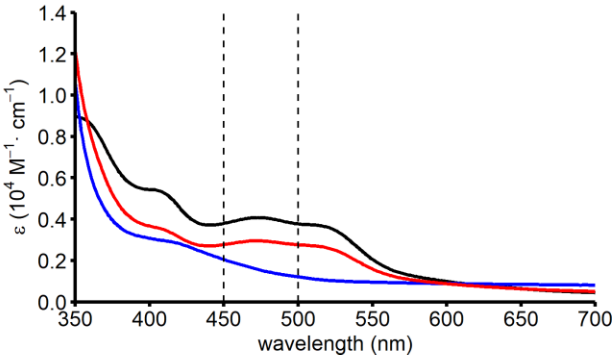

Figure S15. UV/Vis absorption for: a) 200 µL 1 mM **Ru(bda)(pic)$_2$** in 1800 µL pH 1 aqueous HNO_3 (black), b) 200 µL 1 mM **Ru(bda)(pic)$_2$** in 1800 µL of 0.406M CAN in pH 1 HNO_3 treated with 25 µL 10 mM ascorbic acid (blue) and c) 200 µL 1 mM **Ru(bda)(pic)$_2$** in 1800 µL of 0.406M CAN in pH 1 HNO_3 treated with 250 µL 10 mM ascorbic acid (red).

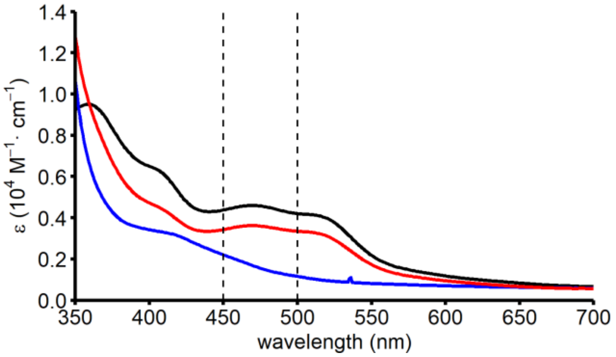

Figure S16. UV/Vis absorption for: a) 200 µL 1 mM **1-*m*Me** in 1800 µL pH 1 aqueous HNO_3 (black), b) 200 µL 1 mM **1-*m*Me** in 1800 µL of 0.406M CAN in pH 1 HNO_3 treated with 25 µL 10 mM ascorbic acid (blue) and c) 200 µL 1 mM **1-*m*Me** in 1800 µL of 0.406M CAN in pH 1 HNO_3 treated with 250 µL 10 mM ascorbic acid (red).

Computational details

DFT calculations were performed in Gaussian 16 Rev C.01.[1] M06 hybrid exchange-correlation meta-functional[2] with LANL2DZ basis set[3] for Ru and 6-31G(d) basis set[4] for all other atoms was used in all calculations. For Ru, a LANL08 effective core potential[5] was employed. All peroxide computations were performed with solvation (water) using a polarizable continuum model.[6] Intermolecular interaction energies were estimated using counterpoise feature with basis set superposition error correction, as implemented in Gaussian 16. Hessian matrix was calculated for each optimized geometry to ensure no imaginary frequencies were present, and local potential energy minimum was reached. Thermochemistry corrections at 300 K were obtained from frequency calculations. Solvent-accessible surface area (SASA) for the axial ligand pairs of was determined with VMD package,[7] using a spherical probe with 1.4 Å radius. SASA of peroxo-dimer of **Ru(bda)(pic)$_2$** was determined to be 384.5 $Å^2$, while areas of individual 4-picoline and 3-picoline ligands were, in average, 247.3 $Å^2$ and 249.4 $Å^2$, respectively.

Table S3. Comparison of computer parameters of various configurations of peroxo-dimers of ***1-mMe***.

	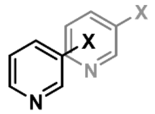 ***m*A**	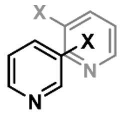 ***mB***	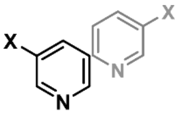 ***m*C**	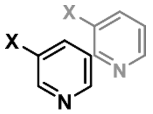 ***m*D**
Electronic energy + thermal Gibbs energy correction	-3228.301761 a.u.	-3228.301059 a.u.	-3228.295365 a.u.	-3228.295046 a.u.
Axial ligands complexation energies	-2.20 kcal/mol<br>-2.20 kcal/mol	-2.62 kcal/mol<br>-2.68 kcal/mol	-0.92 kcal/mol<br>-0.89 kcal/mol	-1.13 kcal/mol<br>-1.12 kcal/mol
∠(Ru-O-O-Ru)	164.2°	164.9°	138.1°	134.1°
Ru-Ru distance	4.54 Å	4.54 Å	4.42 Å	4.39 Å
Axial ligands solvent-accessible surface areas	379.86 $Å^2$<br>380.85 $Å^2$	366.36 $Å^2$<br>372.08 $Å^2$	395.79 $Å^2$<br>394.14 $Å^2$	381.09 $Å^2$<br>385.33 $Å^2$

References

1. Frisch, M. J.; Trucks, G. W.; Schlegel, H. B.; Scuseria, G. E.; Robb, M. A.; Cheeseman, J. R.; Scalmani, G.; Barone, V.; Petersson, G. A.; Nakatsuji, H.; Li, X.; Caricato, M.; Marenich, A. V.; Bloino, J.; Janesko, B. G.; Gomperts, R.; Mennucci, B.; Hratchian, H. P.; Ortiz, J. V.; Izmaylov, A. F.; Sonnenberg, J. L.; Williams; Ding, F.; Lipparini, F.; Egidi, F.; Goings, J.; Peng, B.; Petrone, A.; Henderson, T.; Ranasinghe, D.; Zakrzewski, V. G.; Gao, J.; Rega, N.; Zheng, G.; Liang, W.; Hada, M.; Ehara, M.; Toyota, K.; Fukuda, R.; Hasegawa, J.; Ishida, M.; Nakajima, T.; Honda, Y.; Kitao, O.; Nakai, H.; Vreven, T.; Throssell, K.; Montgomery Jr., J. A.; Peralta, J. E.; Ogliaro, F.; Bearpark, M. J.; Heyd, J. J.; Brothers, E. N.; Kudin, K. N.; Staroverov, V. N.; Keith, T. A.; Kobayashi, R.; Normand, J.; Raghavachari, K.; Rendell, A. P.; Burant, J. C.; Iyengar, S. S.; Tomasi, J.; Cossi, M.; Millam, J. M.; Klene, M.; Adamo, C.; Cammi, R.; Ochterski, J. W.; Martin, R. L.; Morokuma, K.; Farkas, O.; Foresman, J. B.; Fox, D. J. *Gaussian 16 Rev. C.01*, Wallingford, CT, 2016.
2. Zhao, Y.; Truhlar, D. G., The M06 suite of density functionals for main group thermochemistry, thermochemical kinetics, noncovalent interactions, excited states, and transition elements: two new functionals and systematic testing of four M06-class functionals and 12 other functionals. *Theor. Chem. Acc.* **2008,** *120* (1), 215-241.
3. Dunning, T. H.; Hay, P. J., Gaussian Basis Sets for Molecular Calculations. In *Methods of Electronic Structure Theory*, Schaefer, H. F., Ed. Springer US: Boston, MA, 1977; pp 1-27.
4. Hehre, W. J.; Ditchfield, R.; Pople, J. A., Self-Consistent Molecular Orbital Methods. XII. Further Extensions of Gaussian—Type Basis Sets for Use in Molecular Orbital Studies of Organic Molecules. *J. Chem. Phys.* **1972,** *56* (5), 2257-2261.
5. Hay, P. J.; Wadt, W. R., Ab initio effective core potentials for molecular calculations. Potentials for K to Au including the outermost core orbitals. *J. Chem. Phys.* **1985,** *82* (1), 299-310.
6. Cancès, E.; Mennucci, B.; Tomasi, J., A new integral equation formalism for the polarizable continuum model: Theoretical background and applications to isotropic and anisotropic dielectrics. *J. Chem. Phys.* **1997,** *107* (8), 3032-3041.
7. Humphrey, W.; Dalke, A.; Schulten, K., VMD: Visual molecular dynamics. *Journal of Molecular Graphics* **1996,** *14* (1), 33-38.
